# Greater bullying and social exclusion are linked to higher internalising symptoms from ages 10 to 21

**DOI:** 10.1038/s44271-026-00492-x

**Published:** 2026-07-08

**Authors:** Kathryn. E. Bates, Theresa Cheng, Erin C. Dunn, Rogier A. Kievit, Delia Fuhrmann

**Affiliations:** 1 Department of Psychology, Institute of Psychiatry, Psychology and Neuroscience, King’s College London, London, UK; 2 Harvard University, Cambridge, MA USA; 3 Department of Psychology, Stanford University, Stanford, CA USA; 4 Massachusetts General Hospital, Boston, MA USA; 5 Department of Sociology, College of Liberal Arts, Purdue University, West Lafayette, IN USA; 6 Cognitive Neuroscience Department, Donders Institute for Brain, Cognition and Behaviour, Radboud University Medical Center, Nijmegen, The Netherlands

**Keywords:** Signs and symptoms, Development studies

## Abstract

Adolescence has been proposed as a second sensitive period of development with heightened sensitivity to socio-cultural processing. However, age-specific relationships between adverse social experiences and internalising symptoms are unclear. In this study, we used self-reported data at yearly intervals from age 10 to 21 years from the UK Household Longitudinal (UKHLS) Study. We investigated individual differences in how internalising symptoms change with age and whether there are differences in sensitivity to adverse social experiences (bullying and social exclusion). We captured the relationship between bullying and internalising symptoms from age 10 to 15 years (N = 10,285) and social exclusion and internalising symptoms from age 16 to 21 years (N = 11,623) by leveraging latent growth curve models with time-varying covariates. More bullying was associated with greater internalising symptoms, and there was evidence for age-specific sensitivity at yearly intervals from age 10-15 years (estimates = .541-.714, standardised estimates: .270-.400, *p*s < .001, *FDR corrected ps* < .05). From ages 16-21, higher feelings of social exclusion were associated with greater internalising symptoms (est = .312, *p* < .001), and the strength of this relationship remained stable over time. This evidence shows that vulnerability to adverse social experiences varies over age, and more so between age 10-15 years. The findings highlight: 1) the role of adverse social experiences in internalising symptoms with yearly precision, 2) the relevance of age-specific vulnerabilities to adverse social experiences, 3) and the importance of considering sensitivities beyond age 18 years.

## Abstract (web summary)

This study used latent growth models on UK cohort data to examine age-specific sensitivity between internalising symptoms and adverse social experiences. Sensitivity to bullying varied across ages 10 to 15, associations with social exclusion were stable from 16 to 21 years.

## Introduction

Internalising symptoms, including depression, anxiety and worry, are a common experience in adolescence (age 10–24 years)^1–4^. Data from the World Health Organisation reports that at least one in five adolescents globally are found to have depression symptoms^2^, and for around 14% of adolescents, symptoms tip over into diagnoses of mental health conditions according to the Global Burden of Disease Study^1^. A 2022 meta-analysis identified the peak age of onset of mental health conditions as 14.5 years, and the onset of disorders linked to internalising symptoms ranged from 14.5 years for social anxiety to an average of 20.5 years for mood disorders. The meta-analysis also found that nearly half of mental health disorders were first diagnosed before age 18 and 62.5% before 25 years^4^. Research identifying the type and timing of risk factors is key to understanding the origins of the onset and persistence of internalising symptoms in adolescence.

Identifying developmental windows of sensitivity to exposure to adverse experiences has been influential in establishing prevention and intervention efforts for children. Adverse experiences are potentially traumatic events, such as abuse and neglect. Developmental timing of exposure can be captured using a sensitive periods model of development. The sensitive periods model posits that the brain and behaviour may be particularly vulnerable to environmental experiences at specific stages of development^5^. Much of the research around sensitive periods has focused on the early years (e.g. birth to 3 years)^6,7^. This research has made substantial contributions to developmental science, as well as policy and practice. For example, evidence has shown that exposure to adverse experiences in the early years can have lasting effects on neurodevelopment^8^, mental health^9–11^ and physical health^12^. This research has led to the implementation of preventative policies and legislation to protect children from adverse experiences during specific times in development^13–15^. This research shows the theoretical and policy implications of understanding the impact of both the type and timing of adverse experiences in development on the onset and persistence of mental health problems.

Adolescence is a period of extensive structural and functional brain development^16–18^ and heightened sensitivity to socio-cultural processing^19,20^. Characterised by the onset of puberty and development of secondary sex characteristics^21,22^, adolescence is a unique period of development in social^23^, cognitive^24^ and behavioural^25^ domains. As adolescents gain independence, their social lives expand from their immediate family and friends to wider social networks, including peers^26^, partners^27^ and within the community^28^. Adolescence has therefore been conceptualised as a sensitive period for socio-cultural processing^19,20^. At the same time, specific adverse experiences of a social nature become more prominent, including bullying^29^ and social exclusion^30^. For a comprehensive understanding of the link between adversity exposure and mental health problems, research must consider the specific timing and types of adversities most salient in adolescence.

Variability in the timing of adversity exposure during adolescence has been linked to fluctuations in internalising and externalising symptoms^31–36^. Indications of developmental sensitivity are mixed: some have shown adversity occurring between age 10 and 18 years predicted adult psychosocial maladjustment^37^, whereas others have shown the age of first exposure to interpersonal violence was not significantly associated with psychopathology^31^. Studies commonly assess timing effects by comparing relatively broad age groups, rarely assessing exposure at the more fine-grained measurement of individual years. A study published in 2025, however, assessed nine time points between the ages of 4 and 18 years. They tested fluctuations in the relationship between adversity and internalising symptoms, where adverse childhood experiences (ACEs), including emotional abuse and parent divorce, formed a composite score of adversity. Time-varying estimation models testing fluctuations revealed coefficients at age 15 years were three times that of the coefficients at age 5^34^. This research demonstrates the value of a fine-grained approach to testing the timing of adversity exposure in adolescence. However, work is needed to (1) understand the effect of timing of adversity beyond age 18 years and (2) to test how adverse *social* experiences might relate to internalising symptoms in adolescence, during which time individuals may be particularly vulnerable^20,38^.

Adverse social experiences may be more prominent in adolescence. Here, we define adverse social experiences as potentially traumatic events at the interpersonal level, i.e. arising from relationships with others, including peers^38^. Emerging evidence suggests a high prevalence of adverse social experience in adolescence, with around 18–22% reporting experiencing multiple social adversities, including peer relationship problems, sibling bullying and school issues across two cohorts of nearly 15,000 adolescents^39^. This is higher than the reported rate of adverse childhood experiences, which sits around 8–10%^40^. In the present research, two types of adverse social experiences were studied: bullying and social exclusion. Bullying is defined as repeated abuse where the individual is not able to defend themselves^41^. Rates of bullying are high among adolescents: a survey of almost 300,000 participants found that 30% of 12–17-year-olds reported experiencing at least one instance of bullying in the past 30 days^29^. A 2023 meta-analysis showed that children and adolescents who were bullied were 2.77 times more likely to suffer from depression than those who were not bullied^42^. Social exclusion is considered an indirect form of victimisation and is defined as being purposefully rejected or left out^43,44^. It can be a form of bullying; many national surveys have included feeling excluded in their questions about bullying^45^. In a sample aged 11–19 years, social exclusion was found to be negatively associated with wellbeing^46^. Greater internalising symptoms were found in mid-adolescents reporting social exclusion compared to non-excluded adolescents, and the socially excluded group also demonstrated heightened activation in social brain regions (including the dorsal anterior cingulate cortex, subgenual anterior cingulate cortex, and anterior insula)^47^. Models examining timing of adversity exposure, as in ACEs research^34^, should be applied to adverse social experiences in adolescence to clarify age-specific sensitivity and to direct future prevention efforts.

In the current study, we investigated whether there is evidence for developmental sensitivity in the relationship between adverse social experiences (bullying and social exclusion) and internalising symptoms in adolescence. We expected there to be differences in sensitivity at different ages. To test this hypothesis, we used latent growth curve models of internalising symptoms and time-varying covariates capturing adverse social experiences in longitudinal data. Data was used from the UK Household Longitudinal Survey at yearly intervals from age 10 to 21 years (10–15 years: *N* = 10,285, 16–21 years: *N* = 11,623). This modelling approach allowed us to test the timing of adverse social experiences by comparing *stable sensitivity* models to *developmental sensitivity models;* the latter capturing age-specific effects. To test timing effects, it was important to include adolescents beyond age 18. Sufficient bullying data was not available from the age of 16 years, and internalising symptoms were measured with different questionnaires from 10–15 years to 16–21 years. We therefore chose to include a measure social exclusion for age 16–21 years, as this has some conceptual overlap with bullying and both are common in adolescence^43,44^. As a result, we modelled bullying and internalising symptoms from age 10 to 15 years, and social exclusion and internalising symptoms from age 16 to 21 years. Due to this change in measurement, we also focus on comparing effects within each of the 10–15-year sample and the 16–21-year sample, rather than making inferences about differences between samples.

## Methods

### Study design and cohort characteristics

This study used data from the UK Household Longitudinal Survey (UKHLS)^48^ ; a large-scale panel survey collecting data on family life, income, education, employment and well-being and designed to be representative of the UK population. We refer to the variable as 'sex', in line with UKHLS terminology. UKHLS records responses to the question 'What is your sex?' with two options of 'male' or 'female'. UKHLS records participants’ sex as 'inconsistent' if the information is not consistent between waves, and forenames do not indicate whether they were male or female (*N* = 2). Over 100,000 participants have been enrolled in the Understanding Society study so far. Understanding Society was designed as a large, equal-probability sample of the general population of the UK. Ethnic minority and immigrant households were oversampled for meaningful analysis. Understanding Society report close population estimates between the sample and Office for National Statistics data^49^. The University of Essex Ethics Committee has approved all data collection on the Understanding Society main study, COVID-19 surveys and innovation panel waves, including asking consent for all data linkages except to health records. The full ethics statement from Understanding Society is reported in the supplementary materials (Supplementary Method 1). Additional ethical approval for secondary data analysis was not required by King’s College London.

Data from waves 1 to 12 in the UKHLS cohort were downloaded in February 2023. Different measures are used to capture social exclusion and internalising symptoms between ages 10–15 and 16–21; therefore, we fit separate models to ages 10–15 (*N* = 10,285) and ages 16–21 (*N* = 11,623). The main samples reported here consist of 80% of the full sample: we randomly selected 20% of the sample as the exploratory sample to write the analysis scripts and test model convergence, and we then replicated the analysis in the confirmatory sample (the remaining 80%). This approach is recommended in secondary data analysis to control for researcher degrees of freedom when pre-registration is not feasible due to model complexity^50^. The study was not preregistered. The confirmatory sample descriptive statistics and results are reported in this manuscript. The results from the exploratory sample and our main analysis script are available on the Open Science Framework (https://osf.io/mkwhz/overview) and at the bottom of the Supplementary Materials. Some years of age were excluded due to limited repeated measures; however, the pattern of results did not differ between the exploratory and confirmatory samples.

Sex and ethnicity data were retrieved from the stable characteristics file, which is recorded when the participant is first interviewed and verified at each follow-up interview. Demographics are presented in Table 1.

**Table 1:** Sex and ethnicity information for the age 10–15 years and 16–21 years confirmatory samples

	Age 10–15 years		Age 16–21 years	
	*N*	%	*N*	%
**Sex**				
Female	5149	49.9	6117	52.6
Male	5160	50.0	5503	47.3
**Ethnicity**				
White	7412	72.18	8428	72.52
Asian	1519	14.79	1810	15.58
Black	648	6.31	741	6.38
Mixed	584	5.69	490	4.22
Other	84	0.82	140	1.2
Missing	22	0.21	–	–

Values of 10 and below have been removed to prevent statistical disclosure. Sex and ethnicity data were retrieved from the stable characteristics file generated by UKHLS. 'White' included responses to: British/English/Scottish/Welsh/Northern Irish, Irish, Gypsy or Irish traveller, any other white background. 'Asian' includes responses to: Indian, Pakistani, Bangladeshi, Chinese and any other Asian background. 'Black' includes responses to: Caribbean, African and any other Black background. 'Other' includes responses to: Arab, any other ethnic group. 'Missing' refers to missing responses.

### Measures

#### Measures completed by the age 10–15 years sample

To capture internalising symptoms, we used the youth-reported emotional problems scale from the strengths and difficulties questionnaire (SDQ)^51^, which has shown good internal consistency (omega values: 0.73–0.85)^52^. This scale consists of five items measuring emotional problems, including 'I am often unhappy, downhearted or tearful' and 'I worry a lot'. Response options were 'not true', 'somewhat true' and 'certainly true' and scored as 0, 1 and 2, respectively.

Bullying was captured by three items for adolescents aged 10–15 years. The first two items were 'How often do you get physically bullied at school?' and 'How often do you get bullied in other ways at school?'. Response options for these two items were 'never', 'not much (1–3 times in the last 6 months)', 'quite a lot (more than 4 times in the last 6 months' and 'a lot (a few times every week', which were scored as 1, 2, 3 and 4, respectively). The third bullying item was extracted from the SDQ peer problems subscale (a different subscale to the emotional symptoms subscale referred to above), where participants were asked to rate the item according to their experiences in the past 6 months: 'other children or young people pick on me or bully me'. Response options were 'not true', 'somewhat true' and 'certainly true' and scored as 0, 1 and 2, respectively. The bullying score was calculated as the mean across items after individual items were scaled from 0 to 100. Cronbach’s alpha for the bullying items by age in years ranged from 0.74 to 0.80, indicating good internal consistency for each age.

#### Measures completed by the age 16–21 years sample

The SDQ was administered up to age 15 in the UKHLS cohort; therefore, we used the GHQ-12 general health questionnaire^53^ to capture internalising symptoms in the sample aged 16–21 years. SDQ emotional symptoms at age 15 showed strong correlations (>0.310) with GHQ-12 measures between ages 16 and 21 (see Supplementary Fig. S2). The scale consists of 12 questions to indicate how participants have been 'feeling over the last few weeks'. UKHLS recoded the scale (from 1 to 4 in the original, to 0 to 3 in UKHLS), then summed the items, resulting in a score from 0 (least distressed) to 36 (most distressed). The scale includes items such as 'have you been feeling unhappy or depressed?', 'Have you been thinking of yourself as a worthless person?' and 'Have you recently lost much sleep over worry?' Previous studies have shown that the GHQ-12 shows high internal consistency (Cronbach’s alpha: 0.83–0.89) and accurately detects those with minor or major depressive symptoms^54^.

Social exclusion was captured using the 'How often do you feel left out?' item from the UCLA-3 loneliness scale, which has been found to have high internal consistency (Cronbach’s alpha: 0.89–0.94)^55^. Response options were 'hardly ever or never', 'some of the time' and 'often' and scored as 1, 2 and 3, respectively. The bullying items from the youth survey (age 10–15 years) were asked for participants age 16+ at alternate waves from 3 to 13. However, if participants were rated 'not still at school', the item was coded as 'inapplicable' by the data owners. This was the case for 98% of the age 16+ sample. Therefore, there was not sufficient data for bullying to be included in the age 16–21 years sample.

### Data processing and missing data

**Fig. 1:**
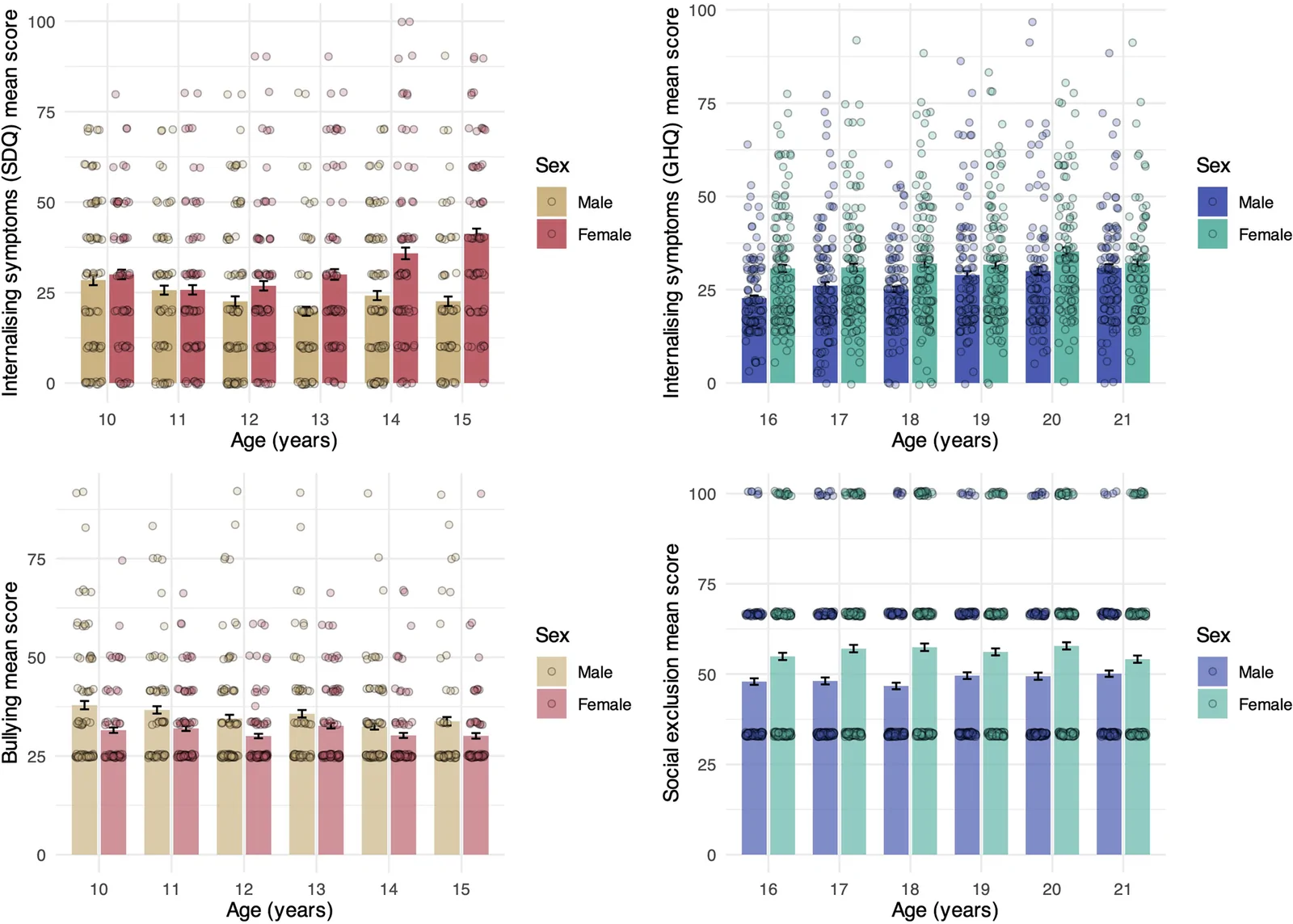
Mean, standard errors and individual data points for all variables split by sex. Internalising symptoms are plotted in the top panel, bullying and social exclusion in the bottom panel. A randomly selected sample of 500 participants is visualised in each plot for visual clarity.

Access to the full dataset can be obtained from the UK Data Service: https://www.understandingsociety.ac.uk/documentation/access-data/. Before modelling, all dependent variables were scaled to values of 0–100 to aid model convergence and interpretation of results. Descriptive statistics for all variables are presented in Fig. 1.

We treated z-scores greater than 5 and less than −5 as missing in order to exclude outliers due to human error as opposed to natural variation in the data^24^. Z-scores were calculated only to identify outliers. This resulted in coding one score in bullying at age 14 and two scores in bullying at age 15 as missing in the age 10–15 sample. No scores were excluded from the sample age 16–21 years. Data for internalising symptoms and bullying were collected in alternate years for ages 10–15. Internalising symptoms were collected in alternate years for ages 16–21, and social exclusion was introduced in wave 9 and collected at yearly intervals. Data retrieved for each measure for each sample and wave are presented in Supplementary Table S1.

The percentage of missing data per observed variable ranged from 52.99 to 90.64% (see Supplementary Table S2). This level of missingness was expected as we organised data by age rather than the year in which the data was collected (wave), because we were interested in developmental trajectories over age. For example, in the age 10–15 years sample, 812 participants had both bullying and internalising symptoms data at ages 10, 12 and 14 years, a further 673 participants had data at age 12 and 14 years and so on (see Fig. S1A, B). Participants with internalising symptom data for at least one time point were included. Out of a possible 6 timepoints, participants in the 10–15 years sample had data for an average of 1.7 timepoints (range = 1–4) for internalising symptoms and 1.7 timepoints for bullying (range = 1–4). Participants in the 16–21 years sample had data for an average of 2.6 timepoints (range = 1–6) for internalising symptoms and 2 timepoints (range = 1–4) for social exclusion. We used full information maximum likelihood (FIML) to handle missing data. The advantage of FIML in longitudinal models is that it uses all available information without imputing data to estimate the model, which maximises statistical power and reduces bias compared to listwise or pairwise deletion and multiple imputation^56,57^.

### Latent growth curve models

We modelled longitudinal trajectories of internalising symptoms and examined heightened sensitivity to adverse social experiences at yearly intervals. Models were fit using the *lavaan* package (version: 0.6-20.2283)^58^. Latent growth curve models allowed us to estimate individual differences at baseline and within-person change over time^59^. We modelled intercepts, slopes and time-varying covariates to capture age-specific strength in the relationship between social adversity and internalising symptoms^60^. Intercepts represent the starting point of a trajectory; in this case, the model-predicted average of internalising symptoms at age 10 in the first model and at age 16 in the second model. The slope is the model-predicted change over time. We fit linear, basis and quadratic slopes and compared the models to investigate which slope best explained internalising symptom trajectories. A significant linear slope represents either an increase or a decrease in symptoms over time. A latent basis slope allows us to freely estimate change between the first time point and the last time point, which could highlight non-linear changes^61^. A quadratic slope also represents non-linear change, but the shape is defined. A positive quadratic slope suggests a U-shape, and a negative slope suggests an inverted U-shape. Time was coded with standard loadings (starting at 0), such that the quadratic term reflects curvature in internalising symptom change across the observed age range rather than a full U-shape trajectory. Bullying and social exclusion are conceptualised as episodic exogenous stressors, rather than a developmental process, and therefore modelled as time-varying covariates. Time-varying covariates (bullying and social exclusion, in this case) allow us to examine time-specific associations between adverse social experiences and internalising symptoms at each age, after accounting for individual differences in the internalising symptom trajectory.

**Fig. 2:**
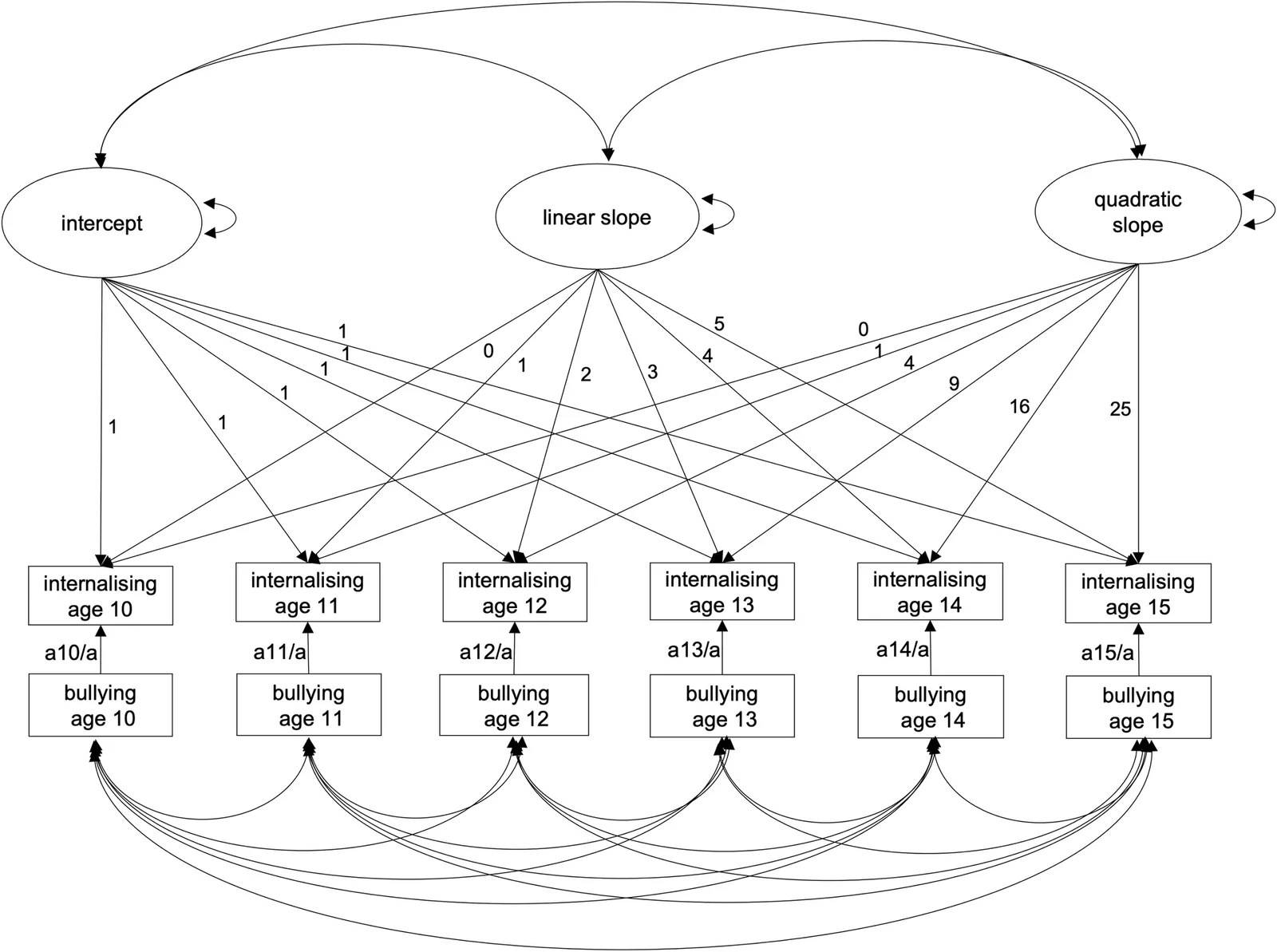
Path schematic for latent growth models. The schematic represents the latent growth models reported in the manuscript (same model fitted to samples aged 10–15 years and 16–21 years,  bullying for the former and social exclusion for the latter). The model fitting the basis slope did not converge and is not visualised here. The parameters 'a10', 'a11' and 'a12', etc. represent the *developmental sensitivity* model where the effect of bullying on internalising symptoms was allowed to vary per age. The parameter 'a' represents the *stable sensitivity* model where the regression effect was constrained to be equal over age.

To identify possible developmental sensitivity, we fit two models (Fig. 2). We first fit the *developmental sensitivity model,* where the regression parameter between internalising symptoms and adverse social experiences was allowed to vary at each age. Second, we fit a *stable sensitivity model* where the regression effect between internalising symptoms and adverse social experiences was constrained to be equal across ages. Covariance parameters were included between the intercept and each slope, between the slope terms and between observed adverse social experiences at each age.

We then compared the model fit of the two models using the chi-square difference test. A significant test indicates that the developmental sensitivity model (with more freely estimated parameters and fewer degrees of freedom) fits the data better than the stable sensitivity model (with fewer parameters and more degrees of freedom). A non-significant chi-squared difference test indicates that the models fit equally well, in which case the more parsimonious model (the stable sensitivity model) would be preferred^62^. Good fit was determined by the following indices^63^: the *χ*^2^ test, the comparative fit index (CFI; good fit = > 0.97, acceptable fit = 0.95–0.97), the standardised root mean square residual (SRMR; good fit = < 0.05, acceptable fit = 0.05–0.10), and the root mean square error of approximation (RMSEA; good fit = < 0.05, acceptable fit = 0.05–0.08). Models were fit using maximum likelihood estimation with robust Huber-White standard errors and scaled test statistic. We have focused on unstandardised estimates of time-varying covariates, bullying and social exclusion, to facilitate interpretation and retain meaningful metrics^64^. We have reported the standardised estimates as well for transparency. Following guidance in the literature, 0.1 is considered small, 0.2 medium and 0.3 large^65^.

To test for sex differences, we fit a multi-group model where parameters of interest were allowed to vary between males and females and a second model where the parameters of interest were constrained to be equal across males and females. We then conducted a chi-squared difference test: if the model without equality constraints fit better, this suggested that there were differences in the parameters between males and females; if the model with equality constraints fit better or if there was no significant difference, this suggested there was no statistically significant evidence for sex differences. Addressing multiple comparisons in structural equation modelling is challenging^66^. To balance Type I and Type II error control, we applied the Benjamini–Hochberg procedure to control the false discovery rate (FDR) for the focal regression parameters within each age group. Most *p*-values were <0.001 and thus negligibly affected by FDR adjustment; adjusted values are reported for the remaining parameters, there were no differences in level of significance for parameters of interest after FDR correction.

### Exploratory analyses

It is possible that adverse social experiences in previous years influenced internalising symptoms in later years, given previous evidence for longitudinal associations between bullying and internalising symptoms^42^. Where there was evidence for the developmental sensitivity model, we next fit a model accounting for adverse social experiences in the year prior as well as the contemporaneous effect. We fit a model where the lagged effect (previous year of social adversity) was constrained to be equal across years and a model where the strength of the lagged effect was allowed to vary per year (Supplementary Fig. S3). We compared the fit of these two models to the developmental sensitivity model.

## Results

### Non-linear trajectories of internalising symptoms between 10 and 15 years

**Fig. 3:**
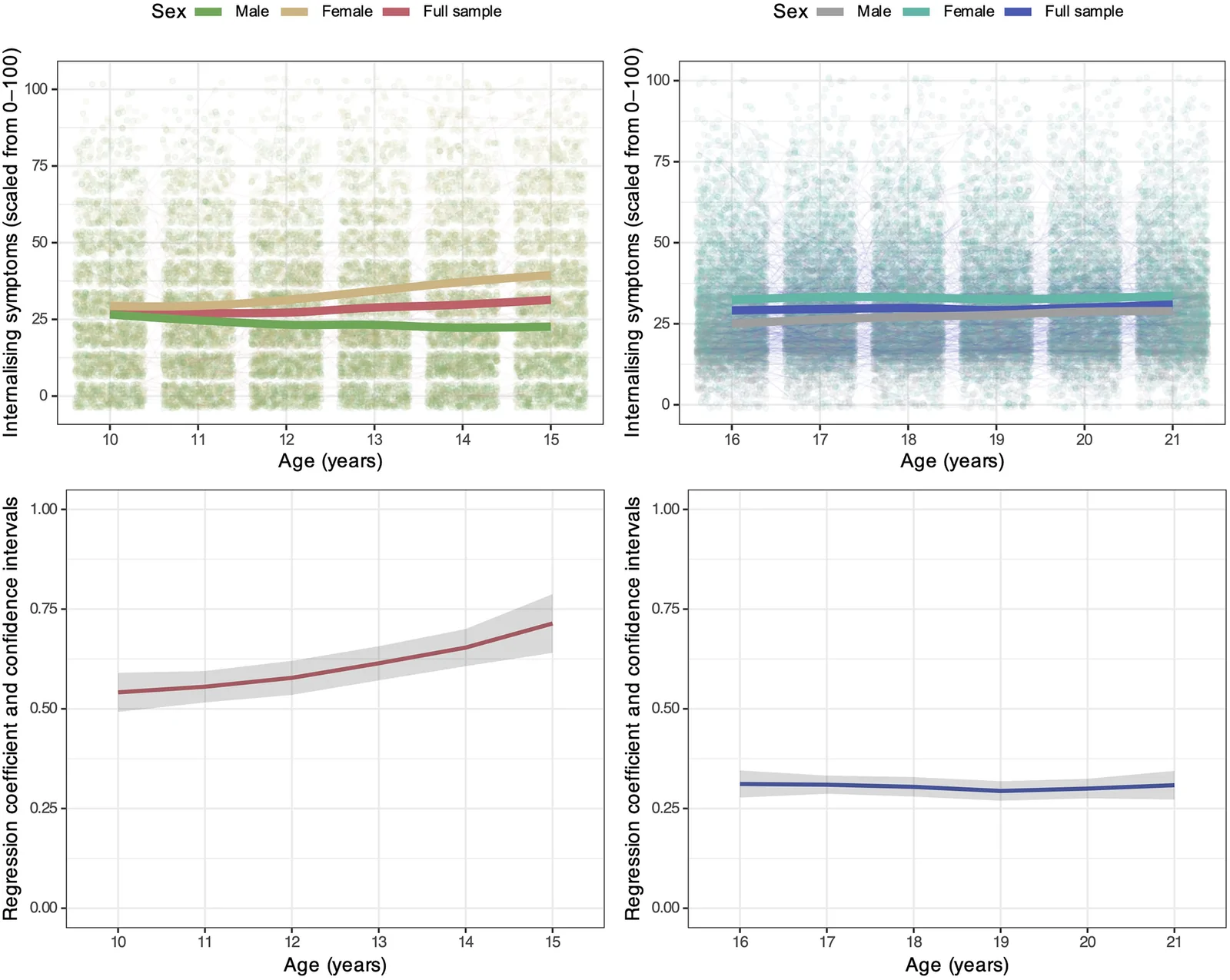
Trajectories of internalising symptoms and relationship between internalising symptoms and adverse social experiences. Mean trajectories of internalising symptoms from ages 10 to 15 (top left) and from ages 16 to 21 (top right) are visualised as spaghetti plots of raw participants' scores (scaled from 0 to 100) for the full sample, males and females. The bottom panels visualise regression coefficients and 95% confidence intervals for the relationship between bullying and internalising symptoms between 10 and 15 years (bottom left) and for the relationship between social exclusion and internalising symptoms between 16 and 21 years (bottom right). The full sample of 10–15-year-olds are visualised (top left). For visualisation purposes, a randomly selected sample of 500 is visualised in the 16–21-year plot (top right), and the regression coefficients from the unconstrained model are presented for both samples.

To determine the trajectory shape of internalising symptoms over time, we fit linear, basis and quadratic growth models. The individual data points and mean trajectories are presented in Fig. 3. We found the latent basis slope was not suitable due to negative variances, and a model, including a linear and quadratic slope fit better than the linear model (Δ*χ*^2^(4) = 43.32, *p* < 0.001; model fit for quadratic and linear model: *χ*^2^(12) = 18.18, *p* = 0.110, RMSEA = 0.007 [0.000, 0.013], CFI = 0.997 and SRMR = 0.054). Model fit statistics are reported in Supplementary Table S3. The intercept indicates that internalising symptom trajectories started around a score of 27.52 (SE = 0.35; *p* < 0.001, 95% CI [26.83, 28.20]). The intercept variance shows that there were significant individual differences in this starting point (est = 214.61, SE = 69.14, *p* = 0.002, 95% CI [79.09, 350.12]). Internalising symptoms declined slightly at first, however, there was an increase in symptoms later between the ages of 13 and 15 years, as evidenced by a significant negative linear slope (est = − 0.811, SE = 0.29, *p* < 0.001, standardised estimate = − 0.130, 95% CI [−0.23, −0.13]) and a significant positive quadratic slope (est = 0.334, SE = 0.06, *p* < 0.001, standardised estimate = 0.232, 95% CI [0.45, 0.23]). There was no evidence for individual differences in the shape of trajectories (linear slope variance: est = 38.92, SE = 43.09, *p* = 0.366, 95% CI [−45.54, 123.38]; quadratic slope variance: est = 2.08, SE = 1.49, *p* = 0.164, 95% CI [−0.85, 5.02]) or the covariance between the intercept and slopes (intercept and linear slope covariance: est = − 1.19, SE = 49.96, *p* = 0.981, 95% CI [−99.12, 96.74]; intercept and quadratic slope covariance: est = − 7.25, SE = 7.72, *p* = 0.348, 95% CI [−22.38, 7.88]).

To test for sex differences, we fit a multi-group model where the slopes and intercepts were allowed to vary between males and females and a second model where slopes and intercepts were constrained to be equal across groups. The model where slopes and intercepts were allowed to vary fit best (Δ*χ*^2^(3) = 795.43, *p* < 0.001; model fit: *χ*^2^(24) = 37.29, *p* = 0.041, RMSEA = 0.010 [0.002, 0.017], CFI = 0.992, SRMR = 0.074), indicating that trajectories of internalising symptoms differed between males and females. Trajectories started at a similar baseline level and then diverged over time (see Fig. 3). Males showed an overall decreasing trend in internalising symptoms (significant negative linear slope: est = − 2.13, SE = 0.40, *p* < 0.001, standardised estimate = − 0.433, 95% CI [−1.34, −0.43]) and this decrease in symptoms decelerated later in development (significant positive quadratic slope: est = 0.266, SE = 0.08, *p* < 0.001, standardised estimate = 0.219, 95% CI [0.41, 0.22]). For females, only the quadratic slope was significant, and this was positive (est = 0.351, SE = 0.08, *p* < 0.001, standardised estimate = 0.222, 95% CI [0.51, 0.22]), suggesting an initial decrease before an increase in internalising symptoms. It should be noted here that due to limitations in the model, we cannot determine whether these differences reflect differences in trajectories of internalising symptoms or differences in the experience of or reporting of internalising symptoms, a property known as measurement invariance. Trajectories for males, females and the full sample are visualised in Fig. 3.

### Evidence for developmental sensitivity in the relationship between bullying and internalising symptoms between 10 and 15 years

We next investigated whether there was evidence for *developmental sensitivity* in the relationship between bullying and internalising symptoms at yearly increments from age 10 to 15 years. In the developmental sensitivity model, we allowed the regression effect of bullying and internalising symptoms to vary across different ages, testing the hypothesis that in different years, the relationship between bullying and internalising symptoms will be more pronounced than in other years. In the stable sensitivity model, we impose equality constraints such that the relationship between bullying and internalising symptoms is the same at different years of age (see Fig. 2 for a schematic of the models). There was a significant difference in fit, indicating that the developmental sensitivity model fit the data better than the stable sensitivity model (Δ*χ*^2^(5) = 17.83, *p* = 0.003), with good absolute fit for the developmental sensitivity model: (*χ*^2^(42) = 136.39, *p* < 0.001, RMSEA = 0.015 [0.012, 0.018], CFI = 0.984, SRMR = 0.057). Higher experience of bullying was associated with higher internalising symptoms at each yearly interval between ages 10 and 15 years with moderate to large estimates (see Table 2). The parameter estimates in Table 2 highlight two key findings: first, the relationship between bullying and internalising symptoms is sizable and statistically significant at each age. Second, a one-unit increase in bullying at age 10 was associated with a 0.541 increase in internalising symptoms, and a 0.714 increase in internalising symptoms at age 15 years.

**Table 2:** Relationship between bullying and internalising symptoms in the sample aged 10–15 years

Age (years)	Estimate	SE	95% CI		*p*	Standardised estimates
			Upper	Lower		
10	0.541	0.03	0.49	0.59	<0.001	0.378
11	0.555	0.02	0.52	0.59	<0.001	0.339
12	0.578	0.02	0.54	0.62	<0.001	0.350
13	0.614	0.02	0.57	0.66	<0.001	0.357
14	0.654	0.02	0.61	0.70	<0.001	0.314
15	0.714	0.04	0.64	0.79	<0.001	0.323

We found evidence for sex differences in the developmental sensitivity model, such that the model where the regression parameters were allowed to vary between males and females showed a good fit and fit better than the constrained model (Δ*χ*^2^(6) = 15.59, *p* = 0.016; *χ*^2^(84) = 192.98, *p* < 0.001, RMSEA = 0.016 [0.013, 0.019], CFI = 0.981, SRMR = 0.076). The coefficients between bullying and internalising symptoms were smaller for males compared to females descriptively. As in the full sample, the coefficients increased with age for both males and females. These effects should be interpreted with caution because we were not able to assess measurement invariance. To outline how these variables behave across males and females, we conducted t-tests and corrected for multiple comparisons, reported in Supplementary Tables S4 and S5. The parameter estimates for males and females from the developmental sensitivity model are presented in Supplementary Table S6.

### Evidence for sensitivity remains after controlling for bullying in previous years

As an exploratory analysis (detailed in Methods, 'Exploratory analyses'), we tested whether there was a significant contemporaneous effect of bullying when the effect of bullying in the previous year was controlled for. We compared the fit of the original developmental sensitivity model to a model where bullying in the previous year was constrained to be equal over age and a model where bullying in the previous year was allowed to vary over age. The chi-squared test suggested the developmental sensitivity model, where the effect of bullying from the previous year was allowed to vary per age fit the data best: (Δ*χ*^2^(4) = 33.31, *p* < 0.001; *χ*^2^(37) = 72.96, *p* < 0.001, RMSEA = 0.010 [0.07, 0.014], CFI = 0.993, SRMR = 0.056). The contemporaneous relationship between bullying and internalising symptoms remained significant at each timepoint from age 11 to age 15 years, after accounting for bullying at the previous time point (*ps* < 0.05, estimates = 0.406–0.600, standardised estimates = 0.194–0.304). The effect of bullying in the previous year was also significant at each time point from age 11 to age 14 years (*ps* < 0.05, estimates = 0.288–0.435, standardised estimates = 0.185–0.241). However, bullying at age 14 was not significantly associated with internalising symptoms at age 15 years (*p* = 0.092). This supports evidence for developmental sensitivity such that bullying in previous years played a role in the subsequent year from age 11 to 14, except at age 15, where bullying occurring in the same year was more strongly associated with higher internalising symptoms. Second, the contemporaneous relationship at each year remained significant after accounting for the previous year. Statistics are reported in Supplementary Table S7.

### Individual differences in the trajectories of internalising symptoms between ages 16 and 21 years

We next examined the relationship between social exclusion and internalising symptoms between 16 and 21 years, following the same data analysis procedure as in the 10–15-year-old sample. Individual data points and the mean trajectory are visualised in Fig. 3. We first assessed the shape of trajectories before testing the developmental sensitivity and stable sensitivity models.

Participants’ internalising symptoms started around 28.54 (SE = 0.195, *p* < 0.001, 95% CI [28.15, 28.92]), and the starting point of the trajectories varied between individuals (est = 149.17, SE = 9.37, *p* < 0.001, 95% CI [130.80, 167.55]). A significant positive linear slope (est = 0.83, SE = 0.161, *p* < 0.001, standardised estimate = 0.144, 95% CI [0.51, 1.15]) suggested an overall increase in internalising symptoms over time. The quadratic slope was not significant (est = − 0.045, SE = 0.031, *p* = 0.154, standardised estimate = − 0.044, 95% CI [−0.106, 0.02]). Although the model including a linear and quadratic slope fit better than the linear model (Δ*χ*^2^(4) = 45.29, *p* < 0.001; quadratic model fit: *χ*^2^(12) = 51.41, *p* < 0.001, RMSEA = 0.017 [0.012, 0.022], CFI = 0.992, SRMR = 0.018), this evidence suggests that, on average, there was no statistically significant evidence for a robust non-linear trend in the sample. Individuals with higher initial internalising symptoms tended to show a slower linear increase in symptoms over time, and those who started with lower internalising symptoms showed a faster increase (negative covariance between intercept and linear slope: est = − 0.27.41, SE = 7.12, standardised effect size = 0.390, 95% CI [−41.36, −13.45]). Together, the results show significant increases in internalised symptoms over time from age 16 to 21 years, with individual differences in the starting point and acceleration of trajectories.

There was evidence for sex differences in the slopes and intercepts such that a model where these parameters were allowed to vary between males and females fit excellently and better than a model where the parameters were constrained across males and females (Δ*χ*^2^(3) = 528.59, *p* < 0.001; *χ*^2^(24) = 69.82, *p* < 0.001, RMSEA = 0.018 [0.013, 0.023], CFI = 0.990, SRMR = 0.023). Males showed an initial increase in symptoms followed by a deceleration of that increase as evidenced by a significant positive linear slope (est = 1.33, SE = 0.22, *p* < 0.001, standardised effect size = 0.207, 95% CI [0.89, 1.75]) and a significant negative quadratic slope (est = − 0.09, SE = 0.04, *p* = 0.031, standardised effect size = 0.079, 95% CI [−0.176, −0.008]). On the other hand, neither the linear slope (est = 0.338, SE = 0.232, *p* = 0.146, standardised effect size = 0.069, 95% CI [−0.12, 0.794]) nor the quadratic slope (est = 0.000, SE = 0.045, *p* = 0.992, standardised effect size = 0.001, 95% CI [−0.09, 0.09]) were significant for females, suggesting no changes in internalising symptoms over time for this group (Fig. 3).

### Strong and stable relationship between social exclusion and internalising symptoms between 16 and 21 years

There was a significant relationship between social exclusion and internalising symptoms, suggesting that higher feelings of social exclusion were linked to higher internalising symptoms (est = 0.305, SE = 0.009, *p* < 0.001); see Fig. 3. Next, we compared a developmental sensitivity model to a stable sensitivity model, where the developmental sensitivity model allowed the regression effect of social exclusion and internalising symptoms to vary between ages. In the stable sensitivity model, we imposed equality constraints such that the regression effect of social exclusion on internalising symptoms was the same at different ages. There was no significant difference between the developmental sensitivity model and the constrained, stable sensitivity model (Δ*χ*^2^(5) = 3.98, *p* = 0.552), indicating that the more parsimonious model with fewer parameters, the stable sensitivity model, should be accepted. Thus, while there was evidence for a strong relationship between social exclusion and internalising symptoms for 16–21-year-olds, there was no evidence that this relationship varied with age.

We then compared a stable sensitivity model, where the regression parameter was allowed to vary between males and females, to a model where the regression parameter was constrained to be equal across males and females. The model that was allowed to vary showed excellent fit and fit better than the constrained model (Δ*χ*^2^(1) = 5.84, *p* = 0.016; *χ*^2^(94) = 355.99, *p* < 0.001, RMSEA = 0.022 [0.022, 0.024], CFI = 0.964, SRMR = 0.083). The regression coefficient was larger in females (est = 0.318 [0.295, 0.341], SE = 0.012, *p* < 0.001, 95% CI [0.29, 0.34]) compared to males (est = 0.276 [0.249, 0.302], SE = 0.014, *p* < 0.001, 95% CI [0.25, 0.30]), but this comparison should be interpreted cautiously given the uncertainties around measurement invariance.

## Discussion

This study assessed whether sensitivity to adverse social experiences in relation to internalising symptoms differed across ages 10–15 years (*N* = 10,285) and 16–21 years (*N* = 11,623) using data from the UK Household Longitudinal Survey cohort. Higher bullying was associated with higher internalising symptoms at each year from age 10 to 15. We found evidence for age-specific sensitivity between bullying and internalising symptoms, such that the model where this relationship was allowed to vary by year fit the data better. Moreover, contemporaneous relationships at each year remained after controlling for bullying in the previous year. In the 16–21-year-old cohort, the social adversity tested was social exclusion. We found evidence for a strong relationship, such that feeling more socially excluded was associated with higher internalising symptoms. This relationship was stable over time, and there was no evidence for age-specific sensitivity between internalising symptoms and social exclusion from age 16 to 21 years. Internalising symptom levels and trajectories showed individual differences, and, for young adolescents, non-linearities. Sex differences were also observed: in the younger sample (age 10–15 years), internalising symptoms increased in females and decreased in males. In later adolescence (16–21 years), symptoms showed there was no evidence for significant change in females. The evidence highlights sensitivity at yearly increments between bullying and internalising symptoms from age 10 to 15 years. The relationship between social exclusion and internalising symptoms from age 16 to 21 did not vary statistically between years. Previous literature has argued adolescents are particularly sensitive to social cues; we extend this to adverse social experiences. This evidence should provide a basis for exploring age-specific antecedents to mental health problems in adolescence.

Research testing age-specific sensitivities between ACEs (caregiver and home-based adversities) and mental health symptoms found evidence for sensitivity at age 15 years^34^. While the estimates descriptively increase to age 15 here, there are overlaps in confidence intervals, so caution is warranted. Nevertheless, we find evidence for age-specific associations at yearly increments, which demonstrates the importance of yearly precision when designing anti-bullying interventions. Heightened sensitivity towards age 15 appears to occur in parallel to sensitivities in other domains at this age. First, research has identified rapid cortical thinning—a neurodevelopmental feature of adolescence—at age 14–15 years^16,18^, which could potentially underpin changes in cognition and psychopathology. In mental health research, prevalence estimates from 2022 suggest the peak onset of mental health disorders is 14.5 years^4^. It is possible that sensitivity to bullying increases beyond age 15 years; however, this was not tested in the present study due to limited measures in the cohort data; further research is needed to test this hypothesis. Building on this evidence, research should test these candidate mechanisms of structural brain development and environmental exposure to better understand how they drive mental health problems.

The stable relationship between higher social exclusion and higher internalising symptoms in the age 16–21 years sample is also notable. Adolescents in the UK, where this cohort is recruited from, can go through significant life transitions at this age, often leaving school, leaving home and starting work or university. This age range can be overlooked in developmental research as they reach the end of adolescence; however, there is some evidence from studies of undergraduate student mental health (age 18+) to draw from. For example, a 2022 study found that social exclusion was positively associated with depression in undergraduate students, and this relationship was mediated by rejection sensitivity (e.g. feeling rejected by peers) and social self-efficacy (e.g. feeling self-conscious about talking to someone new)^67^. Our findings are in line with this research. It is currently unclear, however, whether the relationship between social exclusion and internalising symptoms continues beyond 21 years. The evidence presented in this study demonstrates that this stage of late adolescence should not be overlooked in research seeking to identify risk factors for mental health problems.

We found evidence for sex differences in trajectories of internalising symptoms in both early and late adolescence, and the sensitivity in the relationship between bullying and internalising symptoms in early adolescence (aged 10–15 years). Males showed a decrease in symptoms from age 10 to 15 years, which decelerated towards 15 years. For females at this age, there was an initial decrease followed by an increase up to 15 years. In the 16–21-year cohort, males showed an increase and then a deceleration in that increasing trajectory, and there was no significant change for females. Previous evidence has suggested higher internalising symptoms in females compared to males^22^. This study extends previous evidence by highlighting non-linear change into later adolescence. Concerning the developmental sensitivity model, we found some evidence for a stronger relationship between social adversity and internalising symptoms for females compared to males; however, this comparison should be interpreted with caution. Due to the limitations of the model, we cannot determine whether these sex differences reflect differences in the relationship between internalising symptoms and bullying and/or social exclusion or differences in the experiences or measurement of the constructs modelled here. Research has found that males underreport being bullied: in a previous study, males reported a similar number of occurrences of bullying actions, e.g. being hit or threatened by peers, but were less likely to report being bullied^68^. This could explain the weaker effect of developmental sensitivity in males compared to females found in our study. While the evidence presented here is in line with previous research identifying sex differences between males and females, future research should examine externalising and internalising symptoms in conjunction with social adversity in both males and females to tease apart these relationships.

Our research has theoretical implications for the conceptualisation of adolescence as a period of heightened sensitivity to social processing and extends this to provide evidence for sensitivity to adverse social experiences. The findings fit with the recent conceptualisation of adolescent adverse experiences; this is the notion that adolescents may be more likely to experience and may be more vulnerable to specific adversities, including social adversities of bullying and social exclusion, in this developmental period^38^. Latent growth models with time-varying covariates^60^, as used here, are advantageous in examining the relationship between adversity and mental health in observational data, where randomised control trials are not feasible. The latent growth models fit here handle missing data where models are estimated from all available data at each wave, without the need for imputation, reducing bias^56^. These models could therefore be applied across various longitudinal datasets to further explore the effects of both the type and timing of adverse social experiences across the lifespan. In future research, these models could be leveraged to examine sensitivity to social adversities within the adolescent adverse experiences framework across different levels, e.g. community (school issues, neighbourhood factors) and structural (discrimination, marginalisation) levels.

The findings presented here have practical implications for the development of bullying and social exclusion interventions. Anti-bullying interventions currently often lack developmental sensitivity. This could be one possible reason why we see small effect sizes in intervention studies^69^. A recent review posited that to identify a true sensitive period in development, studies need to test long-term effects or the closure of the sensitive period^20^. Future research should therefore involve participants up to age 24 years (conceptual end of adolescence)^3^; testing the contemporaneous effects of bullying at each year as we have here, and testing how heightened sensitivity in particular years predicts internalising symptoms later in the lifespan. Identifying a potential sensitive period of social adversity and internalising symptoms would allow for the timely administration of both intervention and prevention measures.

### Limitations

We used a large sample representative of the UK general population with a broad age range (10–21 years) and including over 21,000 participants. The use of latent growth models allowed us to capture individual differences in the shape and starting point of trajectories, with the ability to measure non-linear change. The use of time-varying covariates allowed us to capture evidence for developmental sensitivity to social exclusion and bullying. There are some limitations to acknowledge when interpreting the results. First, a limitation of this study is that potential confounding variables, such as socio-economic status, were not accounted for in the models. As a result, caution should be applied in the interpretation of results. Aligning data by age allowed us to model developmental patterns more precisely; however, this approach may also introduce cohort effects, as differences may reflect changes in adolescent mental health across assessment years. While our models allow us to, e.g. control for differences in internalising symptoms at baseline, and to account for exposure in previous years, caution must be applied here in interpreting the causality of effects in the associations reported. Measures of adverse social experiences and internalising symptoms were different between ages 10–15 years and 16–21 years in the UKHLS cohort; this hampers comparability between the models. We need to develop scales that are applicable across adolescence, from ages 10 to 24 years. Cohort data owners may then want to prioritise retaining sensitive measures throughout waves to facilitate research of longitudinal development. Other measurement issues need to be noted here, too. Social exclusion was measured with a single item capturing the feeling left out due to the availability of measures in the UKHLS cohort. This measure may not capture social exclusion fully. There is no consensus on the measurement of social exclusion in the literature. Some studies define social exclusion as whether an individual feels excluded from a social group^46^, and others argue that measures should encapsulate social support and material deprivation^70,71^. Future research should invest in developing a social exclusion measure that can capture this experience with sensitivity across adolescence. While cumulative exposure models provide a valuable lens on social adversity^72^, the available measures and missing data structure in this cohort preclude a robust estimation of cumulative bullying exposure. Future research using repeated, harmonised measures of bullying across adolescence are required to distinguish between cumulative risk and potential age-specific sensitivity effects.

## Conclusion

This study provides evidence that adolescents show variation in the strength of the relationship between adverse social experiences (bullying and social exclusion, in this instance) and internalising symptoms. Higher bullying was associated with higher internalising symptoms at each year of age from 10 to 15 years, even after accounting for bullying experiences in the year prior, and the relationship varied between years of age. Higher social exclusion was associated with higher internalising symptoms at each year of age from 16 to 21 years, and this relationship was stable over time. These findings are in line with evidence that adolescence may be a second sensitive period to socio-cultural processing and highlight that not all adverse social experiences carry equal weight across adolescence. Future studies should identify mechanisms of age-specific sensitivities, apply causal modelling to research questions, and test the closure of sensitive periods where lifespan data is available.

## Supplementary information

Transparent Peer Review File

Supplementary Material

## Data Availability

Data are available via the UK Data Service: https://ukdataservice.ac.uk. Data from the main survey was downloaded: Understanding Society: Waves 1–15, 2009–2024 and Harmonised BHPS: Waves 1–18, 1991–2009.

## Code availability

Our main analysis script is published on the Open Science Framework: https://osf.io/mkwhz/overview
