# Supplementary material for: Greater bullying and social exclusion are linked to higher internalising symptoms from ages 10 to 21
Source: Commun Psychol. 2026 Jul 8;4:151. doi: 10.1038/s44271-026-00492-x (PMC13724274; doi:10.1038/s44271-026-00492-x)
Supplement: Supplementary file 1 [file 44271_2026_492_MOESM1_ESM.pdf]

nature portfolio

# Peer Review File

# Greater bullying and social exclusion are linked to higher internalising symptoms from ages 10 to 21

Corresponding Author: Dr Kathryn Bates

**This file contains all editorial decision letters in order by version, followed by all author rebuttals in order by version.**

Version 0:

Decision Letter:

** Please ensure you delete the link to your author homepage in this e-mail if you wish to forward it to your coauthors **

Dear Dr Bates,

Thank you for your patience during the peer-review process. Your manuscript titled "Internalising Symptoms Show Differential Developmental Sensitivity to Social Exclusion between Ages 10-21" has now been seen by 3 reviewers, whose comments are appended below. You will see that they find your work of some potential interest. However, they have raised quite substantial concerns that must be addressed. In light of these comments, we cannot accept the manuscript for publication, but would be interested in considering a revised version that fully addresses these serious concerns.

We hope you will find the Reviewers' comments useful as you decide how to proceed. Should additional work allow you to address these criticisms, we would be happy to look at a substantially revised manuscript. If you choose to take up this option, please highlight all changes in the manuscript text file, and provide a detailed point-by-point reply to the reviewers.

Please bear in mind that we will be reluctant to approach the reviewers again in the absence of substantial revisions.

Editorially, we consider it important that the revised manuscript address the conceptual and methodological concerns related to the measurement and modeling of bullying vs. social exclusion raised by all reviewers. Please implement the modeling suggestions raised by Reviewer 2.

I am attaching a checklist that details critical reporting requirements for the revised manuscript. Please attend to each item and ensure your manuscript is fully compliant. We are requesting that your manuscript aligns with these requirements as this facilitates the evaluation of your manuscript, reducing delays in re-review and potential future acceptance. If your revised manuscript is not aligned with these requests on major issues, such as those concerning statistics, it may be returned to you for further revisions without re-review. Additional information can be found in our style and formatting guide <a href="https://www.nature.com/documents/commspsychol-style-formatting-guide-accept.pdf">Communications Psychology formatting guide</a>.

If the revision process takes significantly longer than five months, we will be happy to reconsider your paper at a later date, provided it still presents a significant contribution to the literature at that stage.

We would appreciate it if you could keep us informed about an estimated timescale for resubmission, to facilitate our planning.

We are committed to providing a fair and constructive peer-review process. Please do not hesitate to contact us if you wish to discuss the revision in more detail.

Please use the following link to submit your
- revised manuscript,
- point-by-point response to the referees' comments,
- cover letter (as a separate document),
- the Reporting Summary (see below), and
- the completed Editorial Request Table (attached):

Link Redacted

** This url links to your confidential home page and associated information about manuscripts you may have submitted or be reviewing for us. If you wish to forward this email to co-authors, please delete the link to your homepage first **

Thank you for the opportunity to review your work.

Best regards,

Jennifer Bellingtier

Jennifer Bellingtier, PhD
Senior Editor
Communications Psychology

Yana Fandakova
External Editor
Communications Psychology

REVIEWER EXPERTISE:
Reviewer #1 adolescent social development
Reviewer #2 latent growth curve modeling
Reviewer #3 adolescent social development

REVIEWER REPORTS:

Reviewer #1 (Remarks to the Author):

Internalising Symptoms Show Differential Developmental Sensitivity to Social Exclusion between Ages 10-21

This manuscript presents a longitudinal investigation into the developmental sensitivity of adolescents' internalising symptoms in response to bullying and social exclusion across ages 10 to 21. The large, population-based sample and use of latent growth curve modelling represent major strengths. The paper is well-structured and addresses an important topic. However, I have several conceptual, methodological, and presentation-related comments on the manuscript in its current form.

Major
1. The manuscript lacks a coherent and consistent conceptual framework for distinguishing bullying from social exclusion. In the abstract and introduction, bullying is implied to be a form of social exclusion, but later the language implies they are treated as separate constructs. There are also no references given for some of these statemetns (eg.page 2 in the introduction: “Bullying is a common form of social exclusion.” - lacks a reference) Especially since for the age ranges 10-15 years only bullying is measured while for 16-21 years only social exclusion is measured and the findings also show different results (one being time-variant the other being stable) it is important to be clear around the definition and distinction of these two constructs. One could argue that the researchers in fact measured two different things with bullying being a different, more severe and harsh aversive social experience, compared to social exclusion and that this explain the difference in resutls. I recommend that the authors revise the introduction to be very clear around conceptual definitions of these constructs and if/how they overlap (including references to existing literature on this) and then also include a discussion of this in the discussion section. Currently there is a discussion that mainly focuses on measurement consistency but does not touch upon the conceptual issues. Relatedly, at the moment, the authors state that bullying was in fact collected in the age ranges 16-21 but that almost all participants answered “inapplicable”. It is unclear what that means and should be explained in more detail
2. Interpretation of Age-Specific Effects. The paper claims “age-specific sensitivity” to bullying at age 15, but the evidence may reflect cumulative exposure rather than a true developmental window of sensitivity. This is an important distinction that is never mentioned. I wonder if it is possible to conduct sensitivity analyses to test whether the duration of how much bullying has been experienced in the past years rather than sensitivity at any specific age drives the results? I think this might be possible given how I understand the datasets used, so I would recommend the authors should implement such an analysis to help with interpretation of the effects.
3. The claim that the approach in the current paper is “novel” may be overstated. Prior work has used longitudinal modelling to examine sensitivity to social adversity. The authors should clarify what exaclty is novel and how.
4. Altogether, including the models for gender differences, the authors compared 4 different models for each age group (10-15,16-21) – this should be stated somewhere explicitly and I think warrants a statement regarding correction for multiple comparisons. Is this required here? If not, why not?
5. Figures: 5.1 Figure1 lacks clarity. It only includes 2 of the 3 slopes tested which is confusing and it is unclear to me why the authors chose to omit the basis slope from the figure. In addition, the difference between the two models (developmental

sensitivity and stable sensitivity) is pretty straightforward and well explained in the text, so I would consider merging the two figures into one which could have a10/a as the labels connecting the bullying and internalising measures at each age and the figure caption could explain that these represent the two different models. And finally, I think the current visual depiction of the different slopes is not optimal and looks somewhat confusing. I wonder if the authors could visualize the different slopes in a clearer way? 5.2 Figures depicting sex differences (i.e. trajectory plots) are more informative than those showing aggregate data (given the singificant sex differences) and should be moved into the main text. Figure S3 in particular is valuable and should be included in the main text. I would even consider moving Figure 3 into the supplement instead – but alternatively the authors could also merge figures 3 and S3 into one figure included in the main text if they prefer.
6. Interpretation of results: the authors state that effect size increases with age for the 10-15 years data and then later (in the discussion) state that age 15 had the highest effect. What is this based on? The estimates? If that's true, I'm not sure this strong interpretation is warranted. Also, in that case shouldn't the authors instead interpret the standardised estimates? This needs to be explained better. Similarly, the authors state that there was a "strong" effect in the model for the 16-21 years data. What is this based on? The effect size? Please clarify.

As a side note, please add page numbers and line numbers to help with revision.

Minor
• It wasn't clear to me why pre-registration was not feasible after the exploratory phase. Can the authors clarify?
• The conclusion currently reiterates the method and results. It should instead focus on implications, future research directions, and policy relevance.
• References. Several statements in the text were lacking references. For example, the very first statement " Internalising symptoms, including depression, anxiety, and worry, are a common experience in adolescence" only includes a reference for the cited age range but not for the actual statement. Or, page 1 of the introduction " Identifying developmental windows of sensitivity to exposure to adverse experiences has been influential in establishing prevention and intervention efforts for ill-health in children." No reference was given here. Ensure all statements are appropriately sourced.
• In my view, the title does not accurately capture the study's findings or design. The study measured bullying in ages 10-15 and not social exclusion. As stated before, the conceptualisation of bullying as a form of social exclusion was not done convincingly or consistently in the manuscript.
• The abstract could be revised to help with clarity. It currently lacks clarity in framing what previous literature lacks ("developmental specificity and methodology" is vague).
• At the beginning in the introduction there are a few statements on prevalence of internalising symptoms and it should be made clear where these numbers come from (UK?).
• A statement in the introduction states that it is unclear why internalising symptoms arise in adolescents. The authors make it sound like there is a complete lack of knowledge and I think the introduction would benefit strongly if the authors added at least some of the existing research here stating more explicitly what has been shown so far and what is missing rather than simply stating this is unclear. Similarly, they later state "We know less about developmental sensitivty beyond childhood, with a gap in evidence for adolescence in particular". Again, this completely omits any existing research on developmental sensitivity in adolescence and should be revised.
• I think the statement in the introduction on "years lost" could be removed as it isn't really clear what is meant here and in any case I don't think there is a need for justification for why mental health disorders in young people are a problem.
• Sex vs. Gender Terminology. The manuscript uses "sex" throughout, but it is unclear why it refers to biological sex rather than gender identity given the lack of biological measures. At the very least a statement explaining this focus should be included although the authors might reconsider using gender instead of sex.
• The long section detailing when linkage for the different waves was approved could be moved to the supplement.
• Figure 1: why not show all data instead of a subsample? At the very least this choice needs to be explained.

Reviewer #2 (Remarks to the Author):

The manuscript "Internalising Symptoms Show Differential Developmental Sensitivity to SocialExclusion between Ages 10-21" presents the hypothesis that there may be relationships between internalizing problems and bullying (social exclusion) using two periods of age 10-15 and 16-21. The relationship between mental health development and social exclusion is well defined by prior research and provides good background for undertaking this analysis.

The use of a large scale behavioral study to assess these trends and covariances over the developmental period 10-21 is good. However there is the issue of different measures between early and late adolescence.

Methods:
1. Was the bullying item for 10-15 from SDQ removed from the SDQ score and only counted in Bullying? This is not clear from the text.
2. Social exclusion in 16-21 is a single item but the chronbach alpha is given for all items of UCLA-3. It is unclear whether being left out is due to intentional behavior or circumstantial. What can be said about being lonely/depressed and lonely/bullied by this measure?
3. Figure 1 is a good opportunity to show spaghetti plots of a random sample of 500 participants to provide reasoning for the latent trajectory shape. I'd expect to see 500 lines from 10-15 or 16-21 and an average line of all of these.
Results:
1. A table showing the model fits of linear, lin+quad, and latent basis would be good to compare fits.

2. Time starts at 0 according to the SEM figure provided. This would mean only half of the U shape is modeled and because females in 3.1. have no linear slope and only a quadratic growth, indicating that only increasing internalizing symptoms - not an initial decrease as reported.
3. Could measurement invariance be addressed by examining the item responses between males and females?
4. In 3.4 interpretations should be adjusted to account for t=0 as bottom of the U shape.That is, if linear slope is positive and quad is negative, then there is a decreasing amount of growth experienced over age. It may start positive but grow more negative as time goes on.

Comments on Modeling:
Is there a reason that bullying/loneliness is treated as a time varying covariate? Could this not be some construct informed by previous years and should be modeled longitudinally? Could Internalizing problems influence bullying/loneliness? This would be a good candidate for a bivariate latent change score model.

Comments on Bullying/Loneliness:
I believe this could be 2 separate analyses. I am not convinced these are similar and could be two unique processes related to internalizing behaviors. Combining them as one process doesn't make sense without more work to equate these two outcomes.

Reviewer #3 (Remarks to the Author):

This manuscript examines the developmental course of internalising symptoms across adolescence in a UK cohort and examines whether social exclusion is associated with internalizing symptoms. It also examines whether the association of social exclusion and internalizing symptoms changes across adolescence. Considering historical increases in internalizing symptoms in recent cohorts of adolescents and some evidence of increasing levels of loneliness in that same generation, this is a relevant and timely research question and the chosen statistical approach is suited to address this question. Yet, the manuscript would benefit from a bit more conceptual clarity, additional methodological detail, and a more nuanced interpretation that acknowledges key limitations.

Introduction/Conceptual
- Adolescence as a sensitive period from a life course perspective is emphasized heavily throughout the introduction (and also the discussion) of the manuscript. Yet, can that truly be inferred from the current data that does not include information on the association of internalising and social exclusion in earlier (i.e., childhood) or later (i.e., middle adulthood, older adulthood) life stages? Relatedly, differences in the association between internalizing and social exclusion within adolescence are hypothesized. However, the nature of these differences are not described. A more detailed introduction of development within the adolescent period (life is very different for 12yo, 16yo, and 21yo) and what that means for the internalising-social exclusion association would better anticipate this hypothesis.

Methods
- There are several aspects about the study design that remain elusive and that should be clarified for better understanding:
o It is not entirely clear to me how the reported sample size is achieved. I assume that these are participants who participated at least at one assessment. But how many participants participated at each age?
o At how many assessments did participants participate on average?
o In which calendar years were data collected?
o How are participants recruited and are they representative of the UK population or parts of the population?
- It is not quite clear from the description how the variables were standardized. At one point it was mentioned that variables were standardized to a range of 0-100, at another point the manuscript talked about z-standardized variables. From the coefficients that are later reported I assume that it was the former, but wouldn't the latter result in more easily interpretable estimates?

Discussion
- There are several key limitations not mentioned that are important for conceptualising the present results. These should be addressed in the discussion or acknowledged as limitations:
o Other potential covariates that could account for confounding between the social exclusion-internalizing association.
o The limitations in establishing causality or directionality should be explicitly acknowledged, particularly given the cross-sectional nature of the association of interest. Also, there is evidence that internalizing symptoms are prospectively associated with future social exclusion so reverse causation is possible.
o The potential impact of cohort effects should be discussed, given that the data were aligned by age rather than date of assessment and given the aforementioned secular changes in mental health.
o
Smaller comments:
- The description of ethics approvals includes data collections that don't appear directly relevant to this study. Consider referencing a source describing the broader project and focusing on the approvals relevant to the present study.
- Standardized coefficients should be included in the abstract for an easier interpretation.
- The three bullying items were averaged, but one appears to be on a different scale than the other two. This approach should be clarified.

EDITORIAL POLICIES

We ask that you ensure your manuscript complies with our editorial policies and reporting requirements.

To that end, we require revised manuscripts to be accompanied by a completed item: a reporting summary that collects information on study design and procedure.

- <a href="https://www.nature.com/documents/nr-reporting-summary.pdf">Nature Research Reporting Summary</a>

Your revised manuscript can only be sent back to the referees if this checklist is completed and uploaded with the revision.

Notes: If you have submitted a Stage 1 Registered Report, Review, Primer, Comment, or Perspective you do not need to submit these forms. If you have already submitted these forms, you may disregard this request.

* TRANSPARENT PEER REVIEW: Communications Psychology uses a transparent peer review system. This means that we publish the editorial decision letters including Reviewers' comments to the authors and the author rebuttal letters online as a supplementary peer review file. However, on author request, confidential information and data can be removed from the published reviewer reports and rebuttal letters prior to publication. If your manuscript has been previously reviewed at another journal, those Reviewers' comments would not form part of the published peer review file.

** Visit Nature Research's author and referees' website at <a href="http://www.nature.com/authors">www.nature.com/authors</a> for information about policies, services and author benefits**

Communications Psychology is committed to improving transparency in authorship. As part of our efforts in this direction, we are now requesting that all authors identified as ‘corresponding author’ create and link their Open Researcher and Contributor Identifier (ORCID) with their account on the Manuscript Tracking System prior to acceptance. ORCID helps the scientific community achieve unambiguous attribution of all scholarly contributions. You can create and link your ORCID from the home page of the Manuscript Tracking System by clicking on ‘Modify my Springer Nature account’ and following the instructions in the link below. Please also inform all co-authors that they can add their ORCIDs to their accounts and that they must do so prior to acceptance.
https://www.springernature.com/gp/researchers/orcid/orcid-for-nature-research

For more information please visit http://www.springernature.com/orcid

If you experience problems in linking your ORCID, please contact the <a href="http://platformsupport.nature.com/">Platform Support Helpdesk</a>.

Version 1:

Decision Letter:

** Please ensure you delete the link to your author homepage in this e-mail if you wish to forward it to your coauthors **

Dear Dr Bates,

Thank you for your patience during the peer-review process. We sincerely apologize for the delay. Your manuscript titled "Age-specific relationships between adverse social experiences and internalising symptoms in adolescence" has now been seen by 2 reviewers, and I include their comments at the end of this message. Unfortunately, only Reviewer 3 was able to return in this round of review. Therefore, we recruited an additional methods expert. You will see that they both find the work much improved. There are a some remaining modeling concerns, and we would like to consider your responses to these concerns and assess a revised manuscript before we make a final decision on publication.

We therefore invite you to revise and resubmit your manuscript, along with a point-by-point response to the reviewers. Please highlight all changes in the manuscript text file.

Editorially, we consider the questions concerning the modelling of time-varying covariates the most critical and look forward to receiving your responses regarding the modelling of the time-varying covariates in a longitudinal model, the separation of between-person growth process from the within-person deviation from this process as well as covariances with the growth factors.

As you revise the manuscript in response to these issues, please also implement all requests in the attached Mandatory

Revision Requests document. All requirements listed in this document need to be fully met, or the work will be returned to you for further revisions without peer review. This workflow is in place to increase the likelihood that the paper will be accepted for publication. It reduces the number of rounds of revision (and review) and ensures that the reviewers vet a version of the article that is compliant with journal policies. If you have any questions regarding the required revisions, please contact the journal prior to resubmission to avoid a negative outcome.

We are committed to providing a fair and constructive peer-review process. Please don't hesitate to contact us if you wish to discuss the revision in more detail.

Please submit the following items:

- Revised manuscript
- Point-by-point response to the referees' comments
- Mandatory Revision Requests Table (attached).
- Cover letter (as a separate document)

via this link: Link Redacted .

** This url links to your confidential home page and associated information about manuscripts you may have submitted or are reviewing for us. If you wish to forward this email to co-authors, please delete the link to your homepage first **

We hope to receive your revised paper within 8 weeks; please let us know if you aren't able to submit it within this time so that we can discuss how best to proceed. If we don't hear from you, and the revision process takes significantly longer, we may close your file. In this event, we will still be happy to reconsider your paper at a later date, provided it still presents a significant contribution to the literature at that stage.

We look forward to seeing the revised manuscript and thank you for the opportunity to review your work.

Best regards,

Yana Fandakova

Yana Fandakova
External Editor
Communications Psychology

REVIEWER EXPERTISE:

Reviewer #3 adolescent social development
Reviewer #4 latent growth curve modeling

REVIEWER REPORTS:

Reviewer #3 (Remarks to the Author):

Thank you for the opportunity to review the revised version of this manuscript. I appreciate that the authors were very responsive to the comments raised by me and the other reviewers. I have no other comments at this stage.

Reviewer #4 (Remarks to the Author):

In this study, the authors examine the relationship between adverse social experiences, specifically bullying and social exclusion, on internalising symptoms in adolescents from age 10 to 21. It is an interesting study using a large data set from the UK Household Longitudinal Study, and the authors provided a major revision of the first draft, based on the first round of reviews. The study builds upon earlier works on timing effects of adverse childhood experiences and strives to reveal periods of heightened sensitivity for adverse social experiences. I think the study deals with a relevant topic and I was able to follow the manuscript well. The authors responeded to all questions raised by the reviewers in the first round of review. Furthermore, I think the authors do a good job in describing their analysis and the particular latent growth curve model with

time-varying covariates used. Still, I have some questions, which I list below.

1) I want to follow up on a point raised by Reviewer 2, who asked whether the time-varying covariates (TVC) bullying (age 10 to 15) and social exclusion (age 16 to 21) could also be modeled in a longitudinal model (e.g., characterized by some form of growth process or an autoregressive trajectory or both). Based on the reviewer's comment, it seems to me that the authors then included a higher lag of bullying, because they found evidence for developmental sensitivity (i.e., time-specific effects of the TVC on internalsing symptoms) in this model only. In this model, the contemporaneous relationship between bullying and internalising symptoms remained significant (i.e., lag-0-bullying), and lag-1-bullying also significantly predicted internalising symptoms except for age 15. I generally think the inclusion of lags of higher order is a good idea. However, I also wonder if bullying is characterized by a latent growth process which should be modeled, in order to separate a between-person growth process from the within-person deviation from this process (as it is done with interalising symptoms). In particular, my question is why the authors decided to fit the latent growth model with the time-varying covariate discussed in Curran et al. (2014) which focuses on the within-person relationships between the TVC and the outcome, but omits the relationships which (may) exist at the between-level (e.g., is a linear increase in bullying over the years related to a linear increase in internalising symptoms)? Curran et al. (2014) present the latent curve model with structured residuals in the same paper to disentangle these between-person from the within-person components. I am not saying that this model should be used, but I would appreciate it if the authors would comment on why modeling bullying or social exclusion via growth curves or autoregressive trajectories is not of interest here.

2) In the LCM-TVC by Curran et al. (2014), the time-varying covariates are also allowed to covary with the growth factors (see Figure 3 in their paper). From the figure in the current study and the exploratory supplemental materials, this does not seem to be the case here. What is the reason for not including this correlation in the model?

3) Furthermore, the authors write on page 11, lines 288-290: "Time-varying covariates (bullying and social exclusion, in this case) allow us to examine how variables that naturally change over time influence the slope at specific points along the age trajectory." I don't think this is the case in the LCM-TVC, because the slope factor is not regressed on the TVC, or am I misunderstanding this sentence?

4) I don't understand the final sample size of the models - are the 20% taken from the N = 10,285 and N = 11,623, respectively, which are stated on page 6, line 162? Or is this already the 80% the confirmatory analysis in the manuscript is based on?

5) Anyway, I like that the authors split the large sample in an exploratory and a confirmatory sample! I find it interesting that in the exploratory sample age 16 to 21, the developmental sensitivty model also does seem to fit the data better, compared to the confirmatory analysis, where the regression estimates of social exclusion on internalising symptoms are stable over time. This does, in my opinion, not need to be discussed in the main text, but I wonder if the authors have also thought about this? If the (exploratory) sample is drawn at random, I would expect that the results align.

Minor Points

6) On page 8, line 212, the authors refer to the GHQ-12 for measuring "internalising symptoms in the sample aged 10-15 years" in the section regarding the 16 to 21 years sample. I am not sure if this is intended or should rather be the sample aged 16 to 21?

7) On page 14, line 364, the estimate is presented double (est = -.811, estimate = -.811), the same happens in line 365

8) In the figure note of Figure 3, the wording "confidence intervals (+/- 95%)" (line 391) is somewhat unusual, I would rather call it just 95% confidence intervals

9) On page 18, line 453, the authors write that "bullying at age 14 did not significantly predict bullying at age 15 years" - I think this should be internalising symptoms at age 15 instead of bullying?

10) Page 24, line 612 misses a period after "levels"

11) Page 25, line 638 "measures of" is doubled

* TRANSPARENT PEER REVIEW: Communications Psychology uses a transparent peer review system. This means that we publish the editorial decision letters including Reviewers' comments to the authors and the author rebuttal letters online as a supplementary peer review file. However, on author request, confidential information and data can be removed from the published reviewer reports and rebuttal letters prior to publication. If your manuscript has been previously reviewed at another journal, those Reviewers' comments would not form part of the published peer review file.

** Visit Nature Research's author and referees' website at <a href="http://www.nature.com/authors">www.nature.com/authors</a> for information about policies, services and author benefits**

Communications Psychology is committed to improving transparency in authorship. As part of our efforts in this direction, we are now requesting that all authors identified as ‘corresponding author’ create and link their Open Researcher and Contributor Identifier (ORCID) with their account on the Manuscript Tracking System prior to acceptance. ORCID helps the scientific community achieve unambiguous attribution of all scholarly contributions. You can create and link your ORCID from the home page of the Manuscript Tracking System by clicking on ‘Modify my Springer Nature account’ and following the instructions in the link below. Please also inform all co-authors that they can add their ORCIDs to their accounts and that they must do so prior to acceptance.
https://www.springernature.com/gp/researchers/orcid/orcid-for-nature-research

For more information please visit http://www.springernature.com/orcid

If you experience problems in linking your ORCID, please contact the <a href="http://platformsupport.nature.com/">Platform Support Helpdesk</a>.

Version 2:

Decision Letter:

** Please ensure you delete the link to your author homepage in this e-mail if you wish to forward it to your coauthors **

Dear Dr Bates,

Your manuscript titled "Age-specific relationships between adverse social experiences and internalising symptoms from age 10 to 24 years in the UK Household Longitudinal Study" has now been editorially reviewed, and I am delighted to say that we are happy, in principle, to publish a suitably revised version in Communications Psychology.

We therefore invite you to revise your paper one last time to address the remaining editorial requests. At the same time we ask that you edit your manuscript to comply with our format requirements and to maximise the accessibility and therefore the impact of your work.

EDITORIAL REQUESTS:

Please review our specific editorial comments and requests regarding your manuscript in the attached "Editorial Requests Table". Please outline your response to each request in the right hand column. Please upload the completed table with your manuscript files as a Related Manuscript file.

If you have any questions or concerns about any of our requests, please do not hesitate to contact me.

SUBMISSION INFORMATION:

In order to accept your paper, we require the files listed here https://www.nature.com/documents/commsj-file-checklist.pdf .

OPEN ACCESS:

Communications Psychology is a fully open access journal. Articles are made freely accessible on publication. For further information about article processing charges, open access funding, and advice and support from Nature Research, please visit https://www.nature.com/commspsychol/open-access

At acceptance, you will be provided with instructions for completing the open access licence agreement on behalf of all authors. This grants us the necessary permissions to publish your paper. Additionally, you will be asked to declare that all required third party permissions have been obtained, and to provide billing information in order to pay the article-processing charge (APC).

* TRANSPARENT PEER REVIEW: Communications Psychology uses a transparent peer review system. On author request, confidential information and data can be removed from the published reviewer reports and rebuttal letters prior to publication. If you are concerned about the release of confidential data, please let us know specifically what information you would like to have removed. Please note that we cannot incorporate redactions for any other reasons.

* CODE AVAILABILITY: All Communications Psychology manuscripts must include a section titled "Code Availability" at the end of the methods section. We require that the custom analysis code supporting your conclusions is made available in a publicly accessible repository at this stage; please choose a repository that generates a digital object identifier (DOI) for the code; the link to the repository and the DOI must be included in the Code Availability statement. Publication as Supplementary Information will not suffice.

* DATA AVAILABILITY:
All Communications Psychology manuscripts must include a section titled "Data Availability" at the end of the Methods section. More information on this policy, is available in the Editorial Requests Table and at <a

href="http://www.nature.com/authors/policies/data/data-availability-statements-data-citations.pdf">http://www.nature.com/authors/policies/data/data-availability-statements-data-citations.pdf</a>.

Please use the following link to submit the above items:
Link Redacted
** This url links to your confidential home page and associated information about manuscripts you may have submitted or be reviewing for us. If you wish to forward this email to co-authors, please delete the link to your homepage first **

We hope to hear from you within two weeks; please let us know if you need more time.

Best regards,

Jennifer Bellingtier

Jennifer Bellingtier, PhD
Senior Editor
Communications Psychology

Yana Fandakova
External Editor
Communications Psychology

** Visit Nature Research's author and referees' website at <a href="http://www.nature.com/authors">www.nature.com/authors</a> for information about policies, services and author benefits**

**Open Access** This Peer Review File is licensed under a Creative Commons Attribution 4.0 International License, which permits use, sharing, adaptation, distribution and reproduction in any medium or format, as long as you give appropriate credit to the original author(s) and the source, provide a link to the Creative Commons license, and indicate if changes were made.
In cases where reviewers are anonymous, credit should be given to 'Anonymous Referee' and the source.
The images or other third party material in this Peer Review File are included in the article’s Creative Commons license, unless indicated otherwise in a credit line to the material. If material is not included in the article’s Creative Commons license and your intended use is not permitted by statutory regulation or exceeds the permitted use, you will need to obtain permission directly from the copyright holder.

To view a copy of this license, visit https://creativecommons.org/licenses/by/4.0/

**REVIEWER REPORTS:**

**Editor:**

Thank you for your patience during the peer-review process. Your manuscript titled "Internalising Symptoms Show Differential Developmental Sensitivity to Social Exclusion between Ages 10-21" has now been seen by 3 reviewers, whose comments are appended below. You will see that they find your work of some potential interest. However, they have raised quite substantial concerns that must be addressed. In light of these comments, we cannot accept the manuscript for publication, but would be interested in considering a revised version that fully addresses these serious concerns.

We hope you will find the Reviewers' comments useful as you decide how to proceed. Should additional work allow you to address these criticisms, we would be happy to look at a substantially revised manuscript. If you choose to take up this option, please highlight all changes in the manuscript text file, and provide a detailed point-by-point reply to the reviewers.

Please bear in mind that we will be reluctant to approach the reviewers again in the absence of substantial revisions.

Editorially, we consider it important that the revised manuscript address the conceptual and methodological concerns related to the measurement and modeling of bullying vs. social exclusion raised by all reviewers. Please implement the modeling suggestions raised by Reviewer 2.

***Thank you for the opportunity to revise and resubmit our manuscript and for noting the positive evaluation of the paper. We have made extensive revisions in response to the evaluation of our manuscript. We provide a point-by-point response below, quoting relevant pages from the manuscript for your convenience. Our point-by-point response is in bold italics, and the changes made in the manuscript (and pasted here for clarity) are in blue. We have also submitted the revised manuscript with major changes highlighted in blue. For clarity, we have labelled the comments as follows: Reviewer 1, Comment 1; Reviewer 1, Comment 2 etc.***

***To confirm, we have addressed:***

- ***The conceptual concerns related to modelling bullying and social exclusion. We have explained the rationale for investigating adverse social experiences, strengthened the background literature on timing and sensitivity, and rationalised the conceptual framework (see Reviewer 1, Comment 1).***
- ***The modelling concerns raised by reviewers, adding an alternative modelling approach accounting for bullying in previous years (see Reviewer 2, Comment 8).***

**Reviewer #1 (Remarks to the Author):**

Internalising Symptoms Show Differential Developmental Sensitivity to Social Exclusion between Ages 10-21

This manuscript presents a longitudinal investigation into the developmental sensitivity of adolescents' internalising symptoms in response to bullying and social exclusion across ages 10 to 21. The large, population-based sample and use of latent growth curve modelling represent major strengths. The paper is well-structured and addresses an important topic. However, I have several conceptual, methodological, and presentation-related comments on the manuscript in its current form.

***We thank the reviewer for their positive evaluation of our approach in the paper as well as for highlighting the importance of the topic. We have addressed the feedback below.***

Major

**Reviewer 1, Comment 1**: 1. The manuscript lacks a coherent and consistent conceptual framework for distinguishing bullying from social exclusion. In the abstract and introduction, bullying is implied to be a form of social exclusion, but later the language implies they are treated as separate constructs. There are also no references given for some of these statements (eg.page 2 in the introduction: "Bullying is a common form of social exclusion." - lacks a reference) Especially since for the age ranges 10-15 years only bullying is measured while for 16-21 years only social exclusion is measured and the findings also show different results (one being time-variant the other being stable) it is important to be clear around the definition and distinction of these two constructs. One could argue that the researchers in fact measured two different things with bullying being a different, more severe and harsh aversive social experience, compared to social exclusion and that this explain the difference in resutls. I recommend that the authors revise the introduction to be very clear around conceptual definitions of these constructs and if/how they overlap (including references to existing literature on this) and then also include a discussion of this in the discussion section. Currently there is a discussion that mainly focuses on measurement consistency but does not touch upon the conceptual issues. Relatedly, at the moment, the authors state that bullying was in fact collected in the age ranges 16-21 but that almost all participants answered "inapplicable". It is unclear what that means and should be explained in more detail

***We have responded to each of the issues raised and appreciate the opportunity to clarify the conceptual framework and interpretation of the results. Specifically, we have revised the conceptual framework to distinguish between bullying and social exclusion, and ensured the background is appropriately referenced (point 1). We have outlined conceptual issues in the discussion (point 2). We have explained the issue with the bullying measure at age 16-21 years (point 3).***

1. ***We thank the reviewer for highlighting the issue around conceptualising social exclusion and bullying. We agree that that more clarity is needed and have now carefully revised the manuscript to provide it. We now make it clear that our primary aim was to test whether there was evidence for developmental sensitivity in the relationship between <u>adverse social experiences</u> and internalising symptoms. We define adverse social experiences as potentially traumatic events at the interpersonal level, i.e., arising from relationships with others, including peers (Pollmann et al., 2025). This includes both bullying and social exclusion. We conceptualise bullying and social exclusion as related, but not identical phenomena. We have carefully edited the manuscript to make the conceptual framework clearer:***

***Introduction, pages 4-5:***

Adverse social experiences may be particularly salient in adolescence. Here we define adverse social experiences as potentially traumatic events at the interpersonal level, i.e. arising from relationships with others, including peers (Pollmann et al., 2025). Emerging evidence suggests a high prevalence of adverse social experience in adolescence, with around 18-22% reporting experiencing multiple social adversities, including peer relationship problems, sibling bullying, and school issues across two UK cohorts of nearly 15,000 adolescents (Kwok & Inman et al., 2025). This is higher than the reported rate of adverse childhood experiences, which sits around 8-10% (Nweze et al., 2023). In the present research, two types of adverse social experiences were studied: bullying and social exclusion. Bullying is defined as repeated abuse where the individual is not able to defend

themselves (Olweus, 2013). Rates of bullying are high among adolescents: A survey of almost 300,000 participants found that 30% of 12-17-year-olds reported experiencing at least one instance of bullying in the past 30 days (Biswas et al., 2020). A 2023 meta-analysis showed that children and adolescents who were bullied were 2.77 times more likely to suffer from depression than those who were not bullied (Ye et al., 2023). Social exclusion is considered an indirect form of victimisation and defined as being purposefully rejected or left out (Mulvey et al., 2017; Wang et al., 2010). It can be a form of bullying; many national surveys have included feeling excluded in their questions about bullying (Charania et al., 2022). In a sample aged 11-19 years, social exclusion was found to be negatively associated with wellbeing (Arslan, 2018). (…)

2. ***We appreciate the suggestion to be cautious in interpreting differences between the two models given the different constructs measured. We have adjusted our discussion of our findings to better reflect this issue and also now highlight it as limitation:***

***Discussion, page 22:***
"It is possible that sensitivity to bullying increases beyond age 15 years, however this was not tested in the present study due to limited measures in the cohort data; further research is needed to test this hypothesis."

***Discussion, page 24:***
"Measures of measures of adverse social experiences and internalising symptoms were different between ages 10-15 years and 16-21 years in the UKHLS cohort, this hampers comparability between the models."

3. ***We have explained the data availability for bullying more clearly in the methods, and we've highlighted the differences in measurement in the introduction. In this dataset, participants age 16 years and above were <u>not</u> asked to provide information on bullying if they were marked as no longer being at school. This resulted in 98% of participants aged 16+ had no response to bullying items. We have also made sure to focus on <u>within</u> sample differences in each sample (aged 10-15 years and age 16-21 years) rather than <u>between sample</u> differences.***

***Methods, page 9:***
"The bullying items from the youth survey (age 10-15 years) were asked for participants age 16+ at alternate waves from 3 to 13. However, if participants were rated "not still at school", the item was coded as "inapplicable" by the data owners. This was the case for 98% of the age 16+ sample. Therefore, there was not sufficient data for bullying to be included in the age 16-21 years sample."

***Introduction, page 5:***
"To test timing effects, it was important to include adolescents beyond age 18. Sufficient bullying data was not available from age 16 years and internalising symptoms were measured with different questionnaires from 10-15 years and 16-21 years. We therefore chose to include a measure social exclusion for age 16-21 years, as this has some conceptual overlap with bullying and both are common in adolescence (Mulvey et al., 2017; Wang et al., 2010b). As a result, we modelled bullying and internalising symptoms from age 10-15 years, and social exclusion and internalising symptoms from age 16-21 years. Due to this change in measurement, we also focus on comparing effects *within* each of the 10–15-year sample and the 16-21 years sample, rather than making inferences about differences *between* samples."

***Discussion, page 21:***

“This study assessed whether sensitivity to adverse social experiences in relation to internalising symptoms differed across ages 10 to 15 years (N = 10,285) and 16 to 21 years (N = 11,623) using data from the UK Household Longitudinal Survey cohort. Higher bullying was associated with higher internalising symptoms at each year from age 10 to 15. We found evidence for age-specific sensitivity between bullying and internalising symptoms such that the model where this relationship was allowed to vary by year, fit the data better. Moreover, contemporaneous relationships at each year remained after controlling for bullying in the previous year. In the 16-to-21-year-old cohort, the social adversity tested was social exclusion. We found evidence for a strong relationship, such that feeling more socially excluded was associated with higher internalising symptoms. This relationship was stable over time, and there was no evidence for age-specific sensitivity between internalising symptoms and social exclusion from age 16-21 years. Internalising symptom levels and trajectories showed individual differences, and, for young adolescents, non-linearities. Sex differences were also observed: in the younger sample (age 10-15 years), internalising symptoms increased in females and decreased in males. In later adolescence (16-21 years), symptoms showed a U-shape in males and there was no evidence for significant change in females. The evidence highlights sensitivity at yearly increments between bullying and internalising symptoms from age 10-15 years. The relationship between social exclusion and internalising symptoms from age 16-21 did not vary statistically between years. Previous literature has argued adolescents are particularly sensitive to social cues, we extend this to adverse social experiences. This evidence should provide a basis for exploring age-specific antecedents to mental health problems in adolescence.

**Reviewer 1, Comment 2:** Interpretation of Age-Specific Effects. The paper claims “age-specific sensitivity” to bullying at age 15, but the evidence may reflect cumulative exposure rather than a true developmental window of sensitivity. This is an important distinction that is never mentioned. I wonder if it is possible to conduct sensitivity analyses to test whether the duration of how much bullying has been experienced in the past years rather than sensitivity at any specific age drives the results? I think this might be possible given how I understand the datasets used, so I would recommend the authors should implement such an analysis to help with interpretation of the effects.

***We thank the reviewer for the insightful comment regarding the potential role of cumulative exposure. We agree that cumulative effects are important to consider when studying developmental sensitivities. However, the cohort data used in this study are not well-suited to testing cumulative effects for two key reasons: 1) the nature of the available measures, and 2) the structure of missing data across waves.***

***Regarding 1), bullying was assessed in the UKHLS cohort using the items “How often were you physically bullied?” and “How often were you bullied in other ways?” and response options were “Never’, “Not much (1-3 times in the last 6 months)”, “Quite a lot (more than 4 times in the last 6 months” and “A lot (a few times every week)”. To strengthen the construct, we also included the item from the SDQ Peer Problems subscale: “Other children or young people pick on me or bully me” with response options of “Not true”, “Somewhat true”, and “Certainly true”. A cumulative score derived from the current measures would likely conflate short-term fluctuations with long-term exposure and lead to ambiguous interpretation. Measures suitable for constructing cumulative risk scores would ideally include consistent multi-wave assessments using comparable scales, or indices capturing onset, duration, and recurrence of bullying across adolescence (Gilbert et al., 2024).***

***Regarding 2), the cohort’s pattern of missing data across ages limits our ability to model cumulative exposure without introducing bias and convergence issues. Due to limited repeated data across age in bullying and social exclusion items, implementing such methods would lead to substantial loss of power and ambiguous interpretation.***

***We now acknowledge this limitation explicitly in the revised manuscript as follows:***

***Discussion, page 25:***
"While cumulative exposure models provide a valuable lens on social adversity (Gilbert et al., 2024), the available measures and missing data structure in this cohort preclude a robust estimation of cumulative bullying exposure. Future research using repeated, harmonized measures of bullying across adolescence are required to distinguish between cumulative risk and potential age-specific sensitivity effects."

**Reviewer 1, Comment 3:** The claim that the approach in the current paper is "novel" may be overstated. Prior work has used longitudinal modelling to examine sensitivity to social adversity. The authors should clarify what exaclty is novel and how.

***We have removed the original wording, and instead explain the aims of our paper more clearly: In the introduction, we explain previous evidence examining the timing of adversity in adolescence, the gap in the literature addressed, and outline our rationale for investigating adverse social experiences (see Reviewer 1, Comment 1). In the discussion we explain the advantages of the methods used and how they can be utilised in the future.***

***Discussion, page 23-24:***
"Latent growth models with time-varying covariates (Curran et al., 2014), as used here, are advantageous in examining the relationship between adversity and mental health in observational data, where randomised control trials are not feasible. The latent growth models fit here handle missing data where models are estimated from all available data at each wave, without the need for imputation, reducing bias (Enders & Bandalos, 2001). These models could therefore be applied across various longitudinal datasets to further explore the effects of both the type and timing of adverse social experiences across the lifespan. In future research, these models could be leveraged to examine sensitivity to social adversities within the Adolescent Adverse Experiences framework across different levels, e.g., community (school issues) and structural (discrimination and marginalisation) levels."

**Reviewer 1, Comment 4:** Altogether, including the models for gender differences, the authors compared 4 different models for each age group (10-15,16-21) – this should be stated somewhere explicitly and I think warrants a statement regarding correction for multiple comparisons. Is this required here? If not, why not?

***We thank the reviewer for highlighting this issue. Multiple comparison correction in structural equation modelling is a challenging topic (Cribbie, 2007), in part because multiple comparison correction can induce Type II errors in model comparisons, as well as Type I errors for parameter estimates in complex models. At the same time, we agree with the reviewer that not correcting at all, especially for marginally significant effects in large samples, is also problematic.***

***Given that our primary hypothesis concerns age-specific associations between internalising symptoms and adverse social experiences (i.e., regression parameters), we have corrected for multiple comparison on these focal parameters, as recommended by Cribbie (2007). We applied Benjamini-Hochberg procedure to control for false discovery rate (FDR) within each year. Most of the p-values for the regression parameters were < .001 and thus negligibly affected; for transparency, FDR values are reported for the few parameters with p-values above .001. We have explained this in the methods and updated the tables. There were no differences in the level of significance for parameters of interest after FDR correction was applied:***

***Methods, page 12-13:***
"Addressing multiple comparisons in structural equation modelling is challenging (Cribbie, 2007). To balance Type I and Type II error control, we applied the Benjamini-Hochberg procedure to control the false discovery rate (FDR) for the focal regression parameters within each age group. Most p-values were < .001 and thus negligibly affected by FDR adjustment; adjusted values are reported for the remaining parameters, there were no differences in level of significance for parameters of interest after FDR correction."

***Supplementary materials:***

"**Table S4**

Standardised estimates from the developmental sensitivity model accounting for bullying in the previous year

| Age (years) | Effect type | Estimate | SE | 95% CI | | *p* | *FDR* | Standardised estimates |
|---|---|---|---|---|---|---|---|---|
| | | | | *lower* | *upper* | | | |
| 10 | Age-specific | .579 | .03 | .52 | .63 | <.001 | .00016 | .400 |
| 11 | Age-specific | .406 | .08 | .25 | .56 | <.001 | .00016 | .247 |
| 11 | Bullying age 10 | .288 | .09 | .11 | .49 | .002 | .003 | .201 |
| 12 | Age-specific | .475 | .06 | .36 | .59 | <.001 | .00016 | .287 |
| 12 | Bullying age 11 | .327 | .07 | .19 | .47 | <.001 | .00016 | .199 |
| 13 | Age-specific | .528 | .06 | .41 | .65 | <.001 | .00016 | .304 |
| 13 | Bullying age 12 | .319 | .08 | .17 | .47 | <.001 | .00016 | .184 |
| 14 | Age-specific | .408 | .20 | .01 | .80 | .044 | .048 | .194 |
| 14 | Bullying age 13 | .435 | .21 | .03 | .84 | .036 | .044 | .240 |
| 15 | Age-specific | .600 | .12 | .37 | .82 | <.001 | .00016 | .270 |
| 15 | Bullying age 14 | .227 | .13 | -.08 | .49 | .092 | .092 | .106 |

**Reviewer 1, Comment 5:** Figures:
**Reviewer 1, Comment 5.1**: Figure1 lacks clarity. It only includes 2 of the 3 slopes tested which is confusing and it is unclear to me why the authors chose to omit the basis slope from the figure. In addition, the difference between the two models (developmental sensitivity and stable sensitivity) is pretty straightforward and well explained in the text, so I would consider merging the two figures into one which could have a10/a as the labels connecting the bullying and internalising measures at each age and the figure caption could explain that these represent the two different models. And finally, I think the current visual depiction of the different slopes is not optimal and looks somewhat confusing. I wonder if the authors could visualize the different slopes in a clearer way?

***Thank you for the suggestions to improve clarity of our figure. We believe the reviewer is referring to Figure 2 here and have responded accordingly. We have condensed Figure 2 into one schematic as suggested and updated the figure caption. The basis slope is not included in the figure because the model did not converge and***

***the results are therefore not interpreted in the manuscript: we have made this clearer in the caption:***

**"Figure 2**
Path schematic for latent growth models. The schematic represents the latent growth models reported in the manuscript (same model fitted to sample aged 10-15 years and 16-21 years, social exclusion instead of bullying for the latter). Paths are visualised with single headed arrows, covariances and variances with double-headed arrows. The model fitting the basis slope did not converge and is not visualised here. The parameters "a10", "a11", "a12" etc. represent the *developmental sensitivity* model where the effect of bullying on internalising symptoms was allowed to vary per age. The parameters "a" represent the *stable sensitivity* model where the regression effect was constrained to be equal over age."

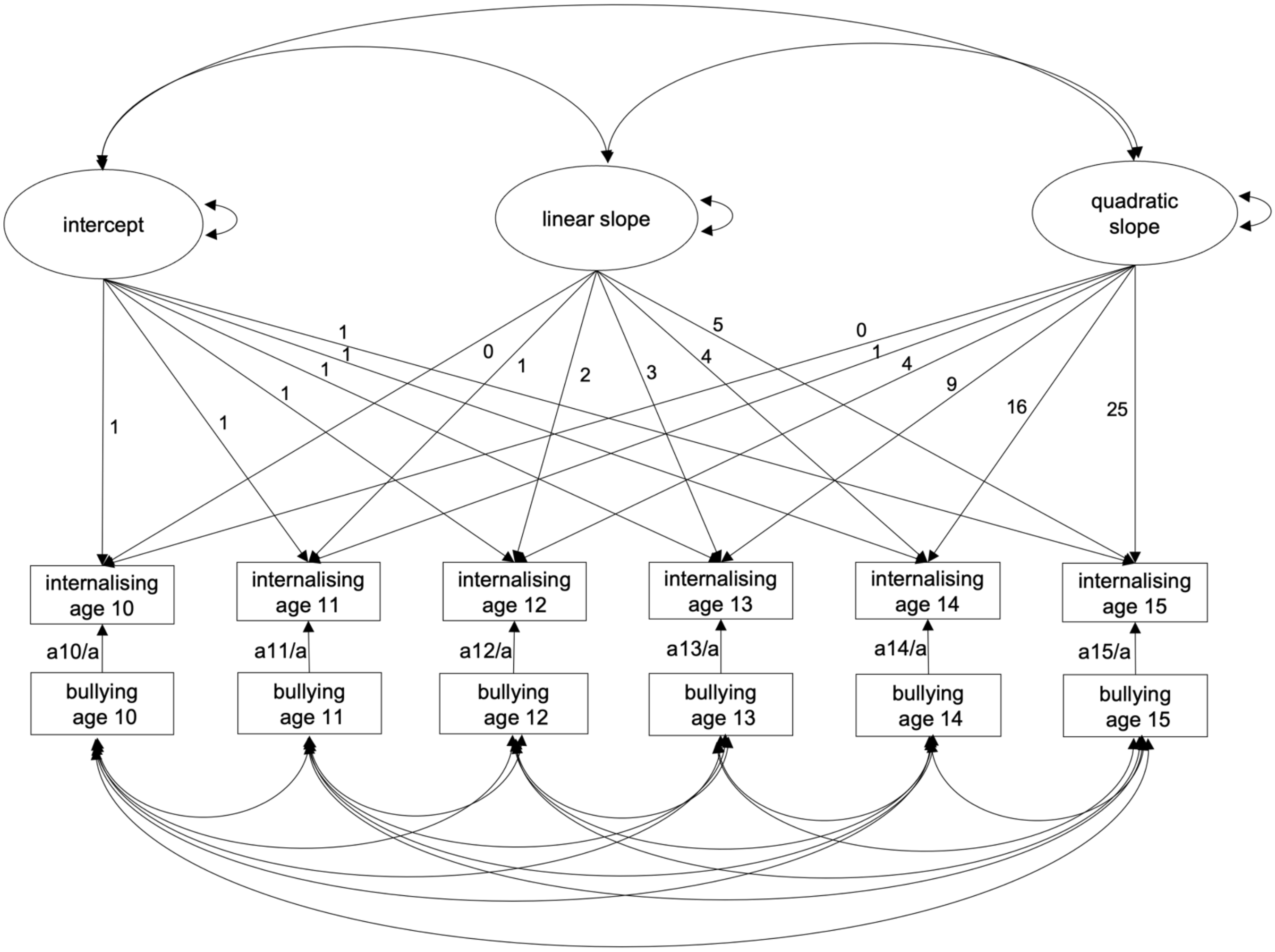


**Reviewer 1, Comment 5.2**: Figures depicting sex differences (i.e. trajectory plots) are more informative than those showing aggregate data (given the singificant sex differences) and should be moved into the main text. Figure S3 in particular is valuable and should be included in the main text. I would even consider moving Figure 3 into the supplement instead – but alternatively the authors could also merge figures 3 and S3 into one figure included in the main text if they prefer.

***Thank you for the suggestion. We have combined Figure S3 and Figure 3 to show trajectories for the full sample and split by sex. We have removed Figure S3 and***

***updated the Figure 3 caption, please note this is now Figure 4 because an additional figure was added in this revision:***

**"Figure 4**

Mean trajectories of internalising symptoms from ages 10 to 15 (top left) and from ages 16 to 21 (top right) are visualised as spaghetti plots of raw participants' scores (scaled from 0-100) for the full sample, males and females. The bottom panels visualise the model-implied unstandardised regression coefficients and confidence intervals (+/- 95%) for the relationship between bullying and internalising symptoms between 10 to 15 years (bottom left) and for the relationship between social exclusion and internalising symptoms between 16 to 21 years (bottom right). For visualisation purposes, a randomly selected sample of 500 is visualised in the 16-to-21-year plot (top right), and the regression coefficients from the unconstrained model are presented for both samples. Note all participants with one or more datapoints were included in the model, therefore only participants with two or more datapoints will have lines connecting datapoints in the figure. "

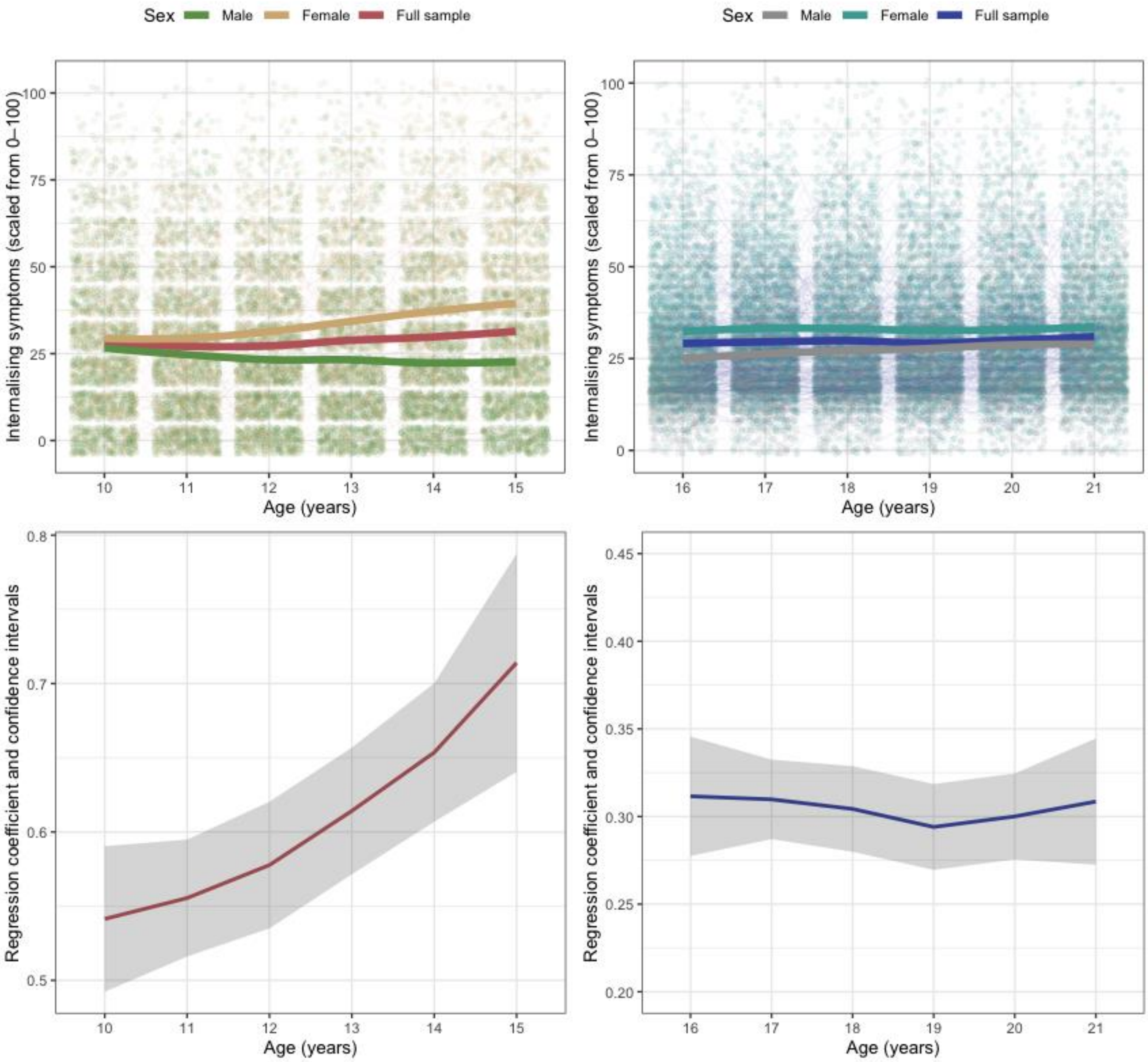

**Reviewer 1, Comment 6:** Interpretation of results: the authors state that effect size increases with age for the 10-15 years data and then later (in the discussion) state that age 15 had the highest effect. What is this based on? The estimates? If that's true, I'm not sure this strong interpretation is warranted. Also, in that case shouldn't the authors instead interpret the standardised estimates? This needs to be explained better. Similarly, the authors state that there was a "strong" effect in the model for the 16-21 years data. What is this based on? The effect size? Please clarify.

***Since the predictors and outcomes of interest in our regression paths are each measured on the same scale, we have interpreted unstandardised estimates as measures of effect size, in line with Flora et al, (2025). We additionally reported the standardised estimates for transparency. The use of unstandardised estimates for time-varying covariates facilitate interpretation and retain meaningful metrics (Baguley, 2009; Feingold, 2019). We have included this explanation, unstandardised and standardised estimates, and we have removed reference to strength to be conservative:***

***Methods, page 12:***
"We have focused on unstandardised estimates of time-varying covariates, bullying and social exclusion, to facilitate interpretation and retain meaningful metrics (Flora et al., 2025). We have reported the standardised estimates as well for transparency."

***Abstract:***
"Adolescence (age 10-24 years) has been proposed as a second sensitive period of development with heightened sensitivity to socio-cultural processing. However, age-specific relationships between adverse social experiences and mental health have yet to be tested. In this study, we used self-reported data at yearly age intervals from age 10 to 21 years from the UK Household Longitudinal (UKHLS) Study. We investigated individual differences in how internalising symptoms change with age and whether there are differences in sensitivity to adverse social experiences (bullying and social exclusion). We captured the relationship between bullying and internalising symptoms from age 10 to 15 years (N=10,285) and social exclusion and internalising symptoms from age 16 to 21 years (N=11,623), by leveraging latent growth curve models with time-varying covariates. More bullying was associated with greater internalising symptoms, and there was evidence for age-specific sensitivity at yearly intervals from age 10-15 years (estimates = .541-.714, *p*s < .001, standardised estimates: .270-.400, *FDR corrected ps* < .05). From ages 16-21, higher feelings of social exclusion were associated with greater internalising symptoms (est = .312, *p* < .001), and the strength of this relationship remained stable over time. This evidence shows that vulnerability to adverse social experiences varies over age, and more so between age 10-15 years. The findings highlight: 1) the role of adverse social experiences in internalising symptoms with yearly precision, 2) the relevance of age-specific vulnerabilities in designing anti-bullying interventions, 3) and the importance of considering sensitivities beyond age 18 years."

**Reviewer 1, Comment 7:** As a side note, please add page numbers and line numbers to help with revision.

***We have added page numbers and line numbers to the manuscript.***

Minor
**Reviewer 1, Comment 8:** It wasn't clear to me why pre-registration was not feasible after the exploratory phase. Can the authors clarify?

***Our approach is following guidelines outlined in Akker et al. (2021). We provided the full output for the exploratory sample results on OSF for transparency, and this is reported in the manuscript. However, combining the exploratory/confirmatory testing***

***and preregistration is an excellent suggestion for future research and we will consider this for the next project.***

**Reviewer 1, Comment 9:** The conclusion currently reiterates the method and results. It should instead focus on implications, future research directions, and policy relevance.

***We have rewritten the conclusion to reflect the implications, future research direction and policy relevance:***

***Conclusion (page 25-26):***
"This study provides evidence that adolescents show variation in the strength of the relationship between adverse social experiences (bullying and social exclusion, in this instance) and internalising symptoms. Higher bullying was associated with higher internalising symptoms at each year of age from 10 to 15 years, even after accounting for bullying experiences in the year prior, and the relationship varied between year of age. Higher social exclusion was associated with higher internalising symptoms at each year of age from 16 to 21 years, and this relationship was stable over time. These findings are in line with evidence that adolescence may be a second sensitive period to socio-cultural processing and highlight that not all adverse social experiences carry equal weight across adolescence. Future studies should identify mechanisms of age-specific sensitivities, apply causal modelling to research questions, and test the closure of sensitive periods where lifespan is available. Policies should prioritise age-tailored anti-bullying initiatives across school and community settings."

**Reviewer 1, Comment 10:** References. Several statements in the text were lacking references. For example, the very first statement " Internalising symptoms, including depression, anxiety, and worry, are a common experience in adolescence" only includes a reference for the cited age range but not for the actual statement. Or, page 1 of the introduction " Identifying developmental windows of sensitivity to exposure to adverse experiences has been influential in establishing prevention and intervention efforts for ill-health in children." No reference was given here. Ensure all statements are appropriately sourced.

***We have carefully revised the manuscript and ensured statements are appropriately referenced, for example:***

***Introduction, page 2:***
"Internalising symptoms, including depression, anxiety, and worry, are a common experience in adolescence (ages 10-24 years; Kieling et al., 2024; Lu et al., 2024; Sawyer et al., 2018; Solmi et al., 2022)."

**Reviewer 1, Comment 11:** In my view, the title does not accurately capture the study's findings or design. The study measured bullying in ages 10-15 and not social exclusion. As stated before, the conceptualisation of bullying as a form of social exclusion was not done convincingly or consistently in the manuscript.

***We have revised the title:***

"Age-specific relationships between adverse social experiences and internalising symptoms in adolescence"

**Reviewer 1, Comment 12:** The abstract could be revised to help with clarity. It currently lacks clarity in framing what previous literature lacks ("developmental specificity and methodology" is vague).

***We have revised the abstract to make the framing clearer, see Reviewer 1, Comment 6.***

**Reviewer 1, Comment 13:** At the beginning in the introduction there are a few statements on prevalence of internalising symptoms and it should be made clear where these numbers come from (UK?).

***We have added this information to the introduction:***

***Introduction, page 2:***
"Data from the World Health Organisation shows that at least one in five adolescents globally report depression symptoms (Lu et al., 2024), and for around 14% of adolescents, symptoms tip over into diagnoses of mental health conditions according to the Global Burden of Disease Study (Kieling et al., 2024)."

**Reviewer 1, Comment 14:** A statement in the introduction states that it is unclear why internalising symptoms arise in adolescents. The authors make it sound like there is a complete lack of knowledge and I think the introduction would benefit strongly if the authors added at least some of the existing research here stating more explicitly what has been shown so far and what is missing rather than simply stating this is unclear. Similarly, they later state "We know less about developmental sensitivty beyond childhood, with a gap in evidence for adolescence in particular". Again, this completely omits any existing research on developmental sensitivity in adolescence and should be revised.

***We appreciate this suggestion and opportunity to strengthen the introduction. We have now included a more comprehensive review of research on developmental sensitivity in adolescence:***

***Introduction, pages 3-4:***
… "Adolescence is a period of extensive structural and functional brain development (Carrick et al., 2025; Fuhrmann et al., 2015, 2022) and heightened sensitivity to socio-cultural processing (Blakemore & Mills, 2014; Cheng et al., 2024). Characterised by the onset of puberty and development of secondary sex characteristics (Blakemore et al., 2010; Pfeifer & Allen, 2021), adolescence is a unique period of development in social (Andrews et al., 2021), cognitive (Fuhrmann et al., 2020) and behavioural (Humphrey & Dumontheil, 2016) domains. As adolescents gain independence, their social lives expand from their immediate family and friends to wider social networks, including peers (Lam, McHala & Crouter, 2014), partners (Moreira et al., 2021) and within the community (see Beyers, Soenens & Vansteenkiste, 2024 for review). Adolescence has therefore been conceptualised as a sensitive period for socio-cultural processing (Blakemore & Mills, 2014; Cheng et al., 2024). At the same time, specific adverse experiences of a social nature become more prominent, including bullying (Biswas et al., 2020), and social exclusion (Li et al., 2021). For a comprehensive understanding of the link between adversity exposure and mental health problems, research must consider the specific *timing* and *types* of adversities most salient in adolescence.

Variability in the timing of adversity exposure during adolescence has been linked to fluctuations in internalising and externalising symptoms (Dunn et al., 2017; Hales et al., 2023; Keiley et al., 2001; Lee et al., 2025; Lei et al., 2022; Lussier et al., 2023; Pickler et al., 2025). Indications of developmental sensitivity are mixed: some have shown adversity occurring between age 10-18 years predicted adult psychosocial maladjustment (Lei et al., 2022), whereas others have shown the age of first exposure to interpersonal violence was not significantly associated with psychopathology (Dunn et al., 2017). Studies commonly assess timing effects by comparing relatively broad age groups, rarely assessing exposure at the more fine-grained measurement of individual years. A study published in 2025, however, assessed nine time points between age 4 and 18 years. They tested fluctuations in the relationship between adversity and internalising symptoms, where adverse childhood

experiences (ACEs) including emotional abuse and parent divorce formed a composite score of adversity. Time-varying estimation models testing fluctuations revealed coefficients at age 15 years were three times that of the coefficients at age 5 (Lee et al., 2025). This research demonstrates the value of a fine-grained approach to testing the timing of adversity exposure in adolescence. However, work is needed to 1) understand the effect of timing of adversity beyond age 18 years and 2) to test how adverse social experiences might relate to internalising symptoms in adolescence, during which time individuals may be particularly vulnerable (Pollmann et al., 2025; Cheng et al., 2024)."

**Reviewer 1, Comment 15:** I think the statement in the introduction on "years lost" could be removed as it isn't really clear what is meant here and in any case I don't think there is a need for justification for why mental health disorders in young people are a problem.

***We have deleted this sentence and replaced it to lead into the literature review and rationale for the present study:***

***Introduction, page 2:***
… "Research identifying the type and timing of risk factors is key to understanding the origins of the onset and persistence of internalising symptoms in adolescence."

**Reviewer 1, Comment 16:** Sex vs. Gender Terminology. The manuscript uses "sex" throughout, but it is unclear why it refers to biological sex rather than gender identity given the lack of biological measures. At the very least a statement explaining this focus should be included although the authors might reconsider using gender instead of sex.

***We have added a statement explaining the UKHLS terminology:***

***Methods, page 6:***
"We refer to the variable as "sex", in line with UKHLS terminology. UKHLS records responses to the question "What is your sex?" with two options of "Male" or "Female". UKHLS records participants' sex as "inconsistent" if the information is not consistent between waves and forenames do not indicate whether they were male or female (N = 2)."

**Reviewer 1, Comment 18:** Figure 1: why not show all data instead of a subsample? At the very least this choice needs to be explained.

***Because the samples are so large (N=10,285 and N=11,623, respectively) the plots showing full sample datapoints were too crowded and it was not possible to see the distribution of scores or the bar plots with the mean score underneath. The subsample is a random sample; therefore, designed to be representative of the main sample.***

**Reviewer #2 (Remarks to the Author):**

The manuscript "Internalising Symptoms Show Differential Developmental Sensitivity to Social Exclusion between Ages 10-21" presents the hypothesis that there may be relationships between internalizing problems and bullying (social exclusion) using two periods of age 10-15 and 16-21. The relationship between mental health development and social exclusion is well defined by prior research and provides good background for undertaking this analysis.

The use of a large scale behavioral study to assess these trends and covariances over the developmental period 10-21 is good. However there is the issue of different measures between early and late adolescence.

***Thank you for your positive evaluation of our approach in the paper. We have addressed your comments in full bellow.***

Methods:

**Reviewer 2, Comment 1:** Was the bullying item for 10-15 from SDQ removed from the SDQ score and only counted in Bullying? This is not clear from the text.

***The bullying item was taken from a different SDQ subscale to the Emotional Symptoms subscale. We have made this clearer in the text.***

***Methods, page 8:***

"The third bullying item was extracted from the SDQ Peer Problems subscale (a different subscale to the Emotional Symptoms subscale referred to above), where participants were asked to rate the item according to their experiences in the past six months: "Other children or young people pick on me or bully me.""

**Reviewer 2, Comment 2:** Social exclusion in 16-21 is a single item but the chronbach alpha is given for all items of UCLA-3. It is unclear whether being left out is due to intentional behavior or circumstantial. What can be said about being lonely/depressed and lonely/bullied by this measure?

***We acknowledge that this item does not distinguish between intentional exclusion or circumstantial exclusion, however it does capture a subjective perception social exclusion. When used in conjunction with other items, it can be used to measure loneliness. As a single-item direct measure, it has been used a proxy of social exclusion in previous research (Dykxhoorn et al., 2024). We have addressed this issue in the discussion:***

***Discussion, page 25:***

"Other measurement issues need to be noted here, too. Social exclusion was measured with a single item capturing feeling left out. This measure may not capture social exclusion fully. There is no consensus on the measurement of social exclusion in the literature. Some studies define social exclusion as whether an individual feels excluded from a social group (Arslan, 2018b), and others argue that measures should encapsulate social support and material deprivation (Allen et al., 2023; van Bergen et al., 2017). Future research should invest in developing a social exclusion measure that can capture this experience with sensitivity across adolescence."

**Reviewer 2, Comment 3:** Figure 1 is a good opportunity to show spaghetti plots of a random sample of 500 participants to provide reasoning for the latent trajectory shape. I'd expect to see 500 lines from 10-15 or 16-21 and an average line of all of these.

***We thank the reviewer for their suggestion. The trajectory shapes are visualised in Figure 4. We agree that plotting raw data is valuable. We note that not every participant will have complete data across all timepoints, so a representative sample of individuals will not show complete lines. Our analysis includes all data using full-information maximum likelihood (FIML) to account for missingness. FIML estimates trajectories based on all available data at all waves has been shown to have reduced bias in estimation (Enders & Bandalos, 2001). The spaghetti plots in Figure 4 include all participants, or a random subsample of 500, and therefore show lines only for participants with two or more datapoints, we have made this clearer in the figure caption:***

“**Figure 4**
Mean trajectories of internalising symptoms from ages 10 to 15 (top left) and from ages 16 to 21 (top right) are visualised as spaghetti plots of raw participants’ scores (scaled from 0-100) for the full sample, males and females. The bottom panels visualise the model-implied unstandardised regression coefficients and confidence intervals (+/- 95%) for the relationship between bullying and internalising symptoms between 10 to 15 years (bottom left) and for the relationship between social exclusion and internalising symptoms between 16 to 21 years (bottom right). For visualisation purposes, a randomly selected sample of 500 is visualised in the 16-to-21-year plot (top right), and the regression coefficients from the unconstrained model are presented for both samples. Note all participants with one or more datapoints were included in the model, therefore only participants with two or more datapoints will have lines connecting datapoints in the figure.”

Results:
**Reviewer 2, Comment 4:** A table showing the model fits of linear, lin+quad, and latent basis would be good to compare fits.

***We have included a table of the model fit in the supplementary materials:***

**Table S3**

Model fit indices for trajectories for both age 10-15 years and age 16-21 years

| Model | $\chi^2$(df) | *p* value | RMSEA (lower 90% CI, upper 90% CI) | *CFI* | SRMR |
|---|---|---|---|---|---|
| | | ***Age 10-15 years*** | | | |
| Linear | 69.92(16) | < .001 | .018 (.014, .023) | .971 | .056 |
| Linear and quadratic | 18.18(12) | .110 | .007 (.000, .013) | .997 | .054 |
| | | ***Age 16-21 years*** | | | |
| Linear | 117.49(16) | < .001 | .023 (.020, .027) | .980 | .031 |
| Linear and quadratic | 51.41(12) | <.001 | .017 (.012, .022) | .992 | .018 |

**Reviewer 2, Comment 5:** Time starts at 0 according to the SEM figure provided. This would mean only half of the U shape is modeled and because females in 3.1. have no linear slope and only a quadratic growth, indicating that only increasing internalizing symptoms - not an initial decrease as reported.

***We thank the reviewer for the careful consideration of our model. The latent growth model was specified with standard loadings used for quadratic LGMs which is the linear growth squared (Felt et al., 2017), where time was centred at the first measurement point (coded as 0). This parameterization does not mean that only half the U-shape is modelled. The quadratic term captures the curvature of internalising trajectories over the observed developmental period. We have added a sentence to explain this in the methods:***

***Methods, page 11:***
"Time was coded with standard loadings (starting at 0), such that the quadratic term reflects curvature in internalising symptom change across the observed age range."

***In terms of the interpretation, we apologise that the results were not clear. The quadratic model included a linear and quadratic term, and this model fit better than the linear model with only the linear term. This is why we have interpreted both the significant negative linear slope (initial decrease) and the significant positive quadratic slope. We have added a sentence to clarify:***

***Results, page 13:***
"We found the latent basis slope was not suitable due to negative variances, and a model including a linear and quadratic slope fit better than a model with a linear slope only ($\Delta\chi^2(4) = 43.32$, $p < .001$; quadratic slope model fit: $\chi^2(12) = 18.18$, $p = .110$, RMSEA = .007 [.000, .013], CFI = .997, SRMR = .054)."

**Reviewer 2, Comment 6:** Could measurement invariance be addressed by examining the item responses between males and females?

***Measurement invariance is a valuable approach to identify group differences. However, our models include adverse social experiences and internalising symptoms as observed, rather than latent variables. Therefore, the usual measurement invariance tests cannot be conducted. To show the reader how these variables behave across males and females, we conducted t-tests and corrected for multiple comparisons. This is reported in Supplementary Materials:***

***Results, page 17:***

"To outline how these variables behave across males and females, we conducted t-tests and corrected for multiple comparisons, reported in Supplementary Table S4."

***Supplementary Tables S4 and S5:***

**Table S4**

Means and standard deviations and Welch's t test results for internalising symptoms and bullying at each age between males and females. Bonferroni corrections applied to p values for 12 comparisons (.05/12 = .004), significant results after correction highlighted in bold.

| Variable | Age | Males (Mean, SD) | Females (Mean, SD) | Welch's t test: t(df), *p* |
|---|---|---|---|---|
| Internalising symptoms | | | | |
| | 10 | 26.55 (22.12) | 29.44 (21.72) | **-3.45(2729), .00057** |
| | 11 | 24.94 (21.78) | 29.28 (22.06) | **-5.24(2789), < .001** |
| | 12 | 23.26 (19.88) | 31.33 (22.94) | **-10.14(2832), < .001** |
| | 13 | 23.23 (20.69) | 34.31 (24.10) | **-13.17(2801), < .001** |
| | 14 | 21.84 (19.69) | 37.32 (24.53) | **-18.84(2797), < .001** |
| | 15 | 22.80 (20.74) | 39.42 (24.37) | **-19.41(2763), < .001** |

| | | | | |
|---|---|---|---|---|
| Bullying | | | | |
| | 10 | 36.84 (16.07) | 34.46 (14.12) | **4.12(2725), < .001** |
| | 11 | 34.84 (14.68) | 32.03 (11.68) | **-5.59(2583), < .001** |
| | 12 | 33.06 (13.97) | 32.17 (12.37) | 1.83(2912), .067 |
| | 13 | 32.79 (13.94) | 32.10 (12.39) | 1.40(2793), .161 |
| | 14 | 30.96 (11.28) | 31.31 (11.30) | -.861(2946), .389 |
| | 15 | 30.81 (11.93) | 29.84 (9.66) | 2.33(2569), .019 |

**Table S5**

Means and standard deviations and Welch's t test results for internalising symptoms and bullying at each age between males and females. Bonferroni corrections applied to p values for 12 comparisons (.05/12 = .004), significant results after correction highlighted in bold.

| **Variable** | **Age** | **Males (Mean, SD)** | **Females (Mean, SD)** | **Welch's t test: t(df), *p*** |
|---|---|---|---|---|
| Internalising symptoms | | | | |
| | 16 | 24.99 (13.63) | 32.30 (16.66) | **-17.35(5137), < .001** |
| | 17 | 26.34 (14.03) | 33.35 (16.89) | **-16.72(5420), < .001** |
| | 18 | 27.15 (14.34) | 33.13 (17.15) | **-13.63(5132), < .001** |
| | 19 | 27.73 (14.72) | 32.62 (16.97) | **-10.84(4926), < .001** |
| | 20 | 29.02 (15.42) | 32.98 (16.85) | **-8.39(4636), < .001** |
| | 21 | 28.83 (14.70) | 33.58 (16.86) | **-10.22(4585), < .001** |
| Social exclusion | | | | |
| | 16 | 49.70 (21.30) | 53.38 (21.85) | -2.80(1060), .005 |
| | 17 | 49.42 (21.21) | 55.42 (23.19) | **-4.76(1232), < .001** |
| | 18 | 48.66 (20.98) | 55.72 (23.03) | **-5.69(1258), < .001** |
| | 19 | 50.48 (21.23) | 54.44 (22.64) | **-3.14(1195), < .001** |
| | 20 | 50.32 (20.98) | 54.74 (22.57) | **-3.43(1130), .0006** |
| | 21 | 49.90 (20.11) | 52.90 (22.29) | -2.36(1074), .018 |

**Reviewer 2, Comment 7:** In 3.4 interpretations should be adjusted to account for t=0 as bottom of the U shape. That is, if linear slope is positive and quad is negative, then there is a decreasing amount of growth experienced over age. It may start positive but grow more negative as time goes on.

***We apologise for the unclear explanation of the factor loadings. We have explained our interpretation and changes to the manuscript in response to Reviewer 2, Comment 5.***

Comments on Modeling:
**Reviewer 2, Comment 8:** Is there a reason that bullying/loneliness is treated as a time varying covariate? Could this not be some construct informed by previous years and should be modeled longitudinally? Could Internalizing problems influence bullying/loneliness? This would be a good candidate for a bivariate latent change score model.

***We thank the reviewers for their thoughtful comments regarding our modelling approach and the suggestions to 1) account for bullying in previous years and 2) consider whether internalising symptoms could influence bullying and social exclusion.***

1. ***We have fit a model to account for bullying in previous years in the 10-15-year-old sample given that we found evidence for developmental sensitivity for this sample only. We fit two exploratory models and compared them to the original developmental sensitivity model. In both models, we included additional regression paths to account for bullying in the previous year: A) The effect of bullying in the previous year on internalising symptoms was constrained to be equal over age, B) The effect of bullying in the previous year was allowed to vary at each age. This allows us to test whether there is an effect of previous year bullying and whether this is different at each age.***

   ***We compared models using a chi-square likelihood ratio test. The results show that the model where the effect of bullying in the previous year was allowed to vary between ages fit the data best ($\Delta\chi^2(4) = 33.31$, $p < .001$; $\chi^2(37) = 72.96$, $p < .001$, RMSEA = .010 [.07, .014], CFI = .993, SRMR = .056). This suggests bullying in the previous year was related to contemporaneous internalising symptoms, and this relationship differed by age. As in the developmental sensitivity model, higher bullying was significantly associated with higher internalising symptoms at all ages. Specifically, the contemporaneous relationship was still significant after accounting for bullying in the previous year (ps < .05, estimates = .406-.600). The effect of bullying in the previous year on internalising at the current year was also significant at each time point from age 11 to age 14 years (ps < .05, estimates = .288-.435), compatible with a longitudinal relationship from age 11 to 14 years. However, bullying at age 14 did not significantly predict bullying at age 15 years (p = .092) but there was a significant contemporaneous effect of bullying and internalising symptoms at age 15 (p < .001, estimate = .600). These results emphasise age-specific relationships between age 10 to 15 years, even after accounting for bullying in the previous year/ We have reported this analysis in the methods, results and discussion in the main manuscript:***

***Methods, page 13:***

### 2.5. Exploratory analyses

It is possible that social adversity in previous years influenced internalising symptoms in later years, given previous evidence for longitudinal associations between bullying and internalising symptoms (Ye et al., 2023). Where there was evidence for the developmental sensitivity model, we next fit a model accounting for social adversity in the year prior as well as the contemporaneous effect. We fit a model where the lagged effect (previous year of social adversity) was constrained to be equal across years and a model where the strength of the lagged effect was allowed to vary per year (Supplementary Figure S3). We compared the fit of these two models to the developmental sensitivity model.

**Supplementary Figure S3**

Path schematic for latent growth model accounted for previous years social adversity (sensitivity analysis). The parameters “b1”, “b2”, “b3” etc. represent the previous year of social adversity, bullying in the example visualised, allowed to vary by age. The parameters labelled “b” represent previous year of social adversity constrained to be equal over age.

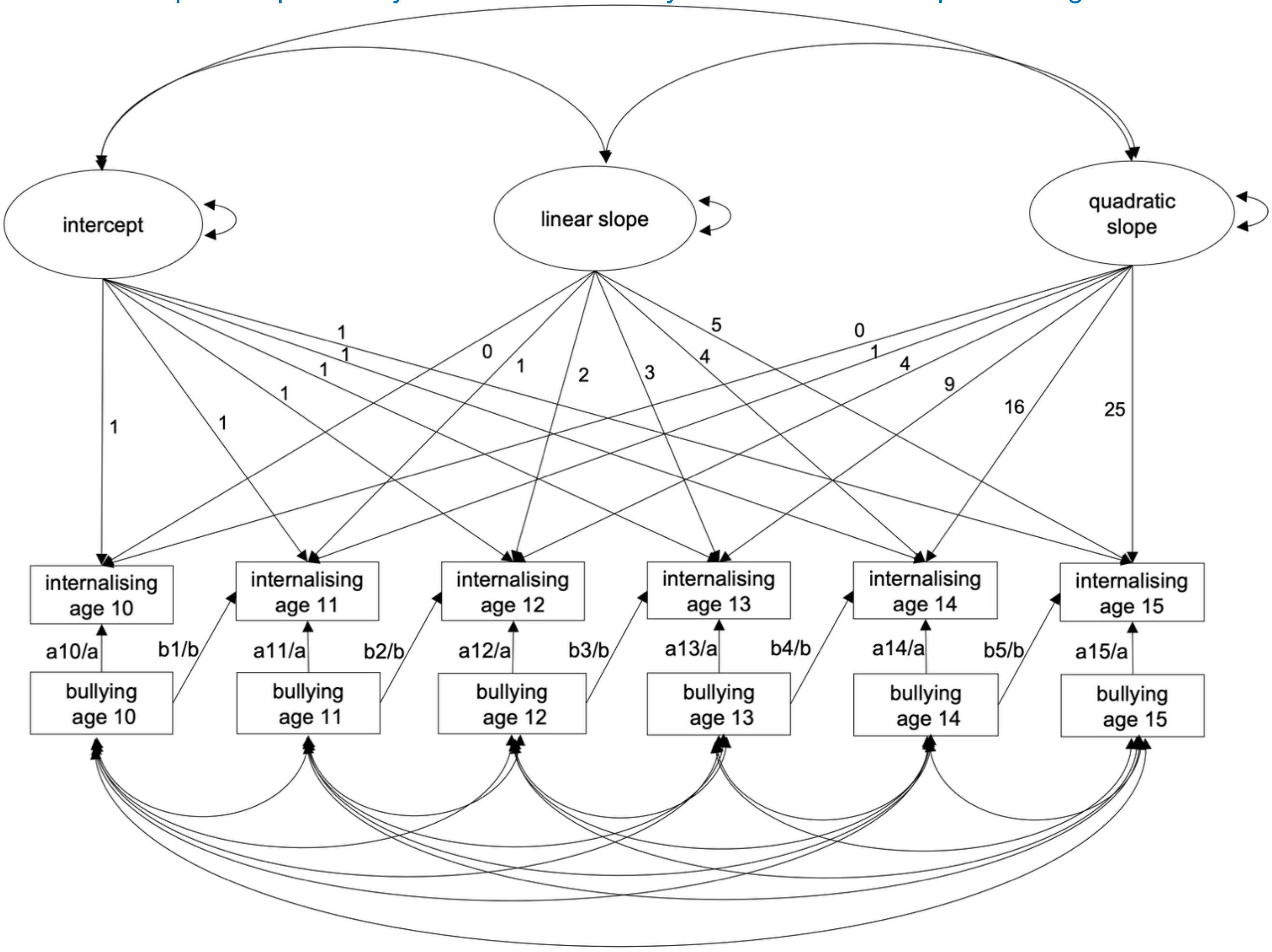


***Results, page 17-18:***

## 2.1. Evidence for contemporaneous sensitivity remains after controlling for bullying in previous years

As an exploratory analysis (detailed in Methods, 2.5.), we tested whether there was a significant contemporaneous effect of bullying, when the effect of bullying in the previous year was controlled for. We compared the fit of the original developmental sensitivity model to a model where bullying in the previous year was constrained to be equal over age and a model whether bullying in the previous year was allowed to vary over age. The chi-squared test suggested the developmental sensitivity model where the effect of bullying from the previous year was allowed to vary per age fit the data best: $\Delta\chi^2(4) = 33.31$, $p < .001$; $\chi^2(37) = 72.96$, $p < .001$, RMSEA = .010 [.07, .014], CFI = .993, SRMR = .056). The contemporaneous relationship between bullying and internalising symptoms remained significant at each timepoint from age 11 to age 15 years, after accounting for bullying at the previous time point (*ps* < .05, estimates = .406-.600, standardised estimates = .194-.304). The effect of bullying in the previous year was also significant at each time point from age 11 to age 14 years (*ps* < .05, estimates = .288-.435, standardised estimates = .185-.241). However, bullying at age 14 did not significantly predict bullying at age 15 years (*p* = .092). This supports evidence for developmental sensitivity such that bullying at previous years played a role in the subsequent year from age 11 to 14, except at age 15 where bullying

occurring in the same year was more strongly associated with higher internalising symptoms. Second, the contemporaneous relationship at each year remained significant after accounting for the previous year. Statistics are reported in Supplementary Table S7.

**Table S7**

Results from the developmental sensitivity model accounting for bullying in the previous year

| Age (years) | Effect type | Estimate | SE | 95% CI lower | 95% CI upper | *p* | *FDR* | Standardised estimates |
|---|---|---|---|---|---|---|---|---|
| 10 | Age-specific | .579 | .03 | .52 | .63 | <.001 | .00016 | .400 |
| 11 | Age-specific | .406 | .08 | .25 | .56 | <.001 | .00016 | .247 |
| 11 | Bullying age 10 | .288 | .09 | .11 | .49 | .002 | .003 | .201 |
| 12 | Age-specific | .475 | .06 | .36 | .59 | <.001 | .00016 | .287 |
| 12 | Bullying age 11 | .327 | .07 | .19 | .47 | <.001 | .00016 | .199 |
| 13 | Age-specific | .528 | .06 | .41 | .65 | <.001 | .00016 | .304 |
| 13 | Bullying age 12 | .319 | .08 | .17 | .47 | <.001 | .00016 | .184 |
| 14 | Age-specific | .408 | .20 | .01 | .80 | .044 | .048 | .194 |
| 14 | Bullying age 13 | .435 | .21 | .03 | .84 | .036 | .044 | .240 |
| 15 | Age-specific | .600 | .12 | .37 | .82 | <.001 | .00016 | .270 |
| 15 | Bullying age 14 | .227 | .13 | -.08 | .49 | .092 | .092 | .106 |

***Discussion, page 20-21:***
"This study assessed whether sensitivity to social adversity in relation to internalising symptoms differed across ages 10 to 15 years (N = 10,285) and 16 to 21 years (N = 11,623) using data from the UK Household Longitudinal Survey cohort. Higher bullying was associated with higher internalising symptoms at each year from age 10 to 15. We found evidence for age-specific sensitivity between bullying and internalising symptoms such that the model where this relationship was allowed to vary by year, fit the data better. Moreover, contemporaneous relationships at each year remained after controlling for bullying in the previous year."

2. ***We cannot rule out bidirectional effects of internalising symptoms and bullying/social exclusion, therefore we have interpreted results as associations rather than implying single direction. We have also outlined this in the discussion as a limitation.***

***Example of association interpretation, page 20:***
"Higher bullying was associated with higher internalising symptoms at each year from age 10 to 15."

***Limitation stated in discussion, page 24:***

“While our models allow us to, e.g. control for differences in internalising symptoms at baseline, and to account for exposure in previous years, caution must be applied here in interpreting the causality of effects in the associations reported.“

**Reviewer 2, Comment 9:** Comments on Bullying/Loneliness:
I believe this could be 2 separate analyses. I am not convinced these are similar and could be two unique processes related to internalizing behaviors. Combining them as one process doesn’t make sense without more work to equate these two outcomes.

***We thank the reviewer for highlighting the issue around conceptualising social exclusion and bullying. We agree that that more clarity is needed and have now carefully revised the manuscript to provide it. We now make it clear that our primary aim was to test whether there was evidence for developmental sensitivity in the relationship between <u>adverse social experiences</u> and internalising symptoms. We define adverse social experiences as potentially traumatic events at the interpersonal level, i.e., arising from relationships with others, including peers (Pollmann et al., 2025). This includes both bullying and social exclusion. We conceptualise bullying and social exclusion as related, but not identical phenomena. We have carefully edited the manuscript to make the conceptual framework clearer:***

***Introduction, pages 3-5:***

“Adolescence is a period of extensive structural and functional brain development (Carrick et al., 2025; Fuhrmann et al., 2015, 2022) and heightened sensitivity to socio-cultural processing (Blakemore & Mills, 2014; Cheng et al., 2024). Characterised by the onset of puberty and development of secondary sex characteristics (Blakemore et al., 2010; Pfeifer & Allen, 2021), adolescence is a unique period of development in social (Andrews et al., 2021), cognitive (Fuhrmann et al., 2020) and behavioural (Humphrey & Dumontheil, 2016) domains. As adolescents gain independence, their social lives expand from their immediate family and friends to wider social networks, including peers (Lam, McHala & Crouter, 2014), intimate partners (Moreira et al., 2021) and within the community (see Beyers, Soenens & Vansteenkiste, 2024 for review). Adolescence has therefore been conceptualised as a sensitive period for socio-cultural processing (Blakemore & Mills, 2014; Cheng et al., 2024). At the same time, specific adverse experiences of a social nature become more prominent, including bullying (Biswas et al., 2020), and social exclusion (Li et al., 2021). For a comprehensive understanding of the link between adversity exposure and mental ill-health, research must consider the specific *timing* and *types* of adversities most salient in adolescence.

Variability in the timing of adversity exposure during adolescence has been linked to fluctuations in internalising and externalising symptoms (Dunn et al., 2017; Hales et al., 2023; Keiley et al., 2001; Lee et al., 2025; Lei et al., 2022; Lussier et al., 2023; Pickler et al., 2025). Indications of developmental sensitivity are mixed: some have shown adversity occurring between age 10-18 years predicted adult psychosocial maladjustment (Lei et al., 2022), whereas others have shown the age of first exposure to interpersonal violence was not significantly associated with psychopathology (Dunn et al., 2017). Studies commonly assess timing effects by comparing relatively broad age groups, rarely assessing exposure at the more fine-grained measurement of individual years. A study published in 2025, however, assessed nine time points between age 4 and 18 years. They tested fluctuations in the relationship between adversity and internalising symptoms, where adverse child experiences (ACEs) including emotional abuse and parent divorce formed a composite score of adversity. Time-varying estimation models testing fluctuations revealed coefficients at age 15 years were three times that of the coefficients at age 5 (Lee et al., 2025). This emerging research demonstrates the value of a fine-grained approach to testing the timing of adversity exposure in adolescence. However, work is needed to 1) understand the effect of timing of adversity beyond age 18 years and 2) to test how adverse social experiences might

relate to internalising symptoms in adolescence, during which time individuals may be particularly vulnerable (Pollmann et al., 2025; Cheng et al., 2024).

Adverse social experiences may be more prominent in adolescence. Here we define adverse social experiences as potentially traumatic events at the interpersonal level, i.e. arising from relationships with others, including peers (Pollmann et al., 2025). Emerging evidence suggests a high prevalence of adverse social experience in adolescence, with around 18-22% reporting experiencing multiple social adversities, including peer relationship problems, sibling bullying, and school issues across two UK cohorts of nearly 15,000 adolescents (Kwok & Inman et al., 2025). This is higher than the reported rate of adverse childhood experiences, which sits around 8-10% (Nweze et al., 2023). We studied two types of adverse social experiences: bullying and social exclusion. Bullying is defined as repeated abuse where the individual is not able to defend themselves (Olweus, 2013). Rates of bullying are high among adolescents: A survey of almost 300,000 participants found that 30% of 12-17-year-olds reported experiencing at least one instance of bullying in the past 30 days (Biswas et al., 2020). A 2023 meta-analysis showed that children and adolescents who were bullied were 2.77 times more likely to suffer from depression than those who were not bullied (Ye et al., 2023). Social exclusion is considered an indirect form of victimisation and defined as being purposefully rejected or left out (Mulvey et al., 2017; Wang et al., 2010a). It can be a form of bullying; many national surveys have included feeling excluded in their questions about bullying (Charania et al., 2022). In a sample aged 11-19 years, social exclusion was found to be negatively associated with wellbeing (Arslan, 2018a). Greater internalising symptoms were found in mid-adolescents reporting social exclusion compared to non-excluded adolescents, and the socially excluded group also demonstrated heightened activation in social brain regions (including the dorsal anterior cingulate cortex, subgenual anterior cingulate cortex, and anterior insula) (Rudolph et al., 2016). Models examining timing of adversity exposure, as in ACEs research (Lee et al., 2025), need to be applied to adverse social experiences in adolescence to clarify age-specific sensitivity and to direct future prevention efforts.

**Reviewer #3 (Remarks to the Author):**

This manuscript examines the developmental course of internalising symptoms across adolescence in a UK cohort and examines whether social exclusion is associated with internalizing symptoms. It also examines whether the association of social exclusion and internalizing symptoms changes across adolescence. Considering historical increases in internalizing symptoms in recent cohorts of adolescents and some evidence of increasing levels of loneliness in that same generation, this is a relevant and timely research question and the chosen statistical approach is suited to address this question. Yet, the manuscript would benefit from a bit more conceptual clarity, additional methodological detail, and a more nuanced interpretation that acknowledges key limitations.

***Thank you for your positive evaluation of the manuscript and acknowledging both the timeliness and relevance. We appreciate your feedback and have addressed your comments in full bellow.***

**Reviewer 3, Comment 1:** Introduction/Conceptual
- Adolescence as a sensitive period from a life course perspective is emphasized heavily throughout the introduction (and also the discussion) of the manuscript. Yet, can that truly be inferred from the current data that does not include information on the association of internalising and social exclusion in earlier (i.e., childhood) or later (i.e., middle adulthood, older adulthood) life stages? Relatedly, differences in the association between internalizing and social exclusion within adolescence are hypothesized. However, the nature of these differences are not described. A more detailed introduction of development within the adolescent period (life is very different for 12yo, 16yo, and 21yo) and what that means for the internalising-social exclusion association would better anticipate this hypothesis.

***We thank the reviewer for the thoughtful consideration of the conceptual framework. We have revised the introduction to explain evidence for the timing of adverse social experiences, i.e., at different stages of adolescence as pointed out, and provided further rationale for examining age-specific effects of bullying and social exclusion:***

***Introduction, pages 3-5:***

"Adolescence is a period of extensive structural and functional brain development (Carrick et al., 2025; Fuhrmann et al., 2015, 2022) and heightened sensitivity to socio-cultural processing (Blakemore & Mills, 2014; Cheng et al., 2024). Characterised by the onset of puberty and development of secondary sex characteristics (Blakemore et al., 2010; Pfeifer & Allen, 2021), adolescence is a unique period of development in social (Andrews et al., 2021), cognitive (Fuhrmann et al., 2020) and behavioural (Humphrey & Dumontheil, 2016) domains. As adolescents gain independence, their social lives expand from their immediate family and friends to wider social networks, including peers (Lam, McHala & Crouter, 2014), intimate partners (Moreira et al., 2021) and within the community (see Beyers, Soenens & Vansteenkiste, 2024 for review). Adolescence has therefore been conceptualised as a sensitive period for socio-cultural processing (Blakemore & Mills, 2014; Cheng et al., 2024). At the same time, specific adverse experiences of a social nature become more prominent, including bullying (Biswas et al., 2020), and social exclusion (Li et al., 2021). For a comprehensive understanding of the link between adversity exposure and mental ill-health, research must consider the specific *timing* and *types* of adversities most salient in adolescence.

Variability in the timing of adversity exposure during adolescence has been linked to fluctuations in internalising and externalising symptoms (Dunn et al., 2017; Hales et al., 2023; Keiley et al., 2001; Lee et al., 2025; Lei et al., 2022; Lussier et al., 2023; Pickler et al., 2025). Indications of developmental sensitivity are mixed: some have shown adversity occurring between age 10-18 years predicted adult psychosocial maladjustment (Lei et al., 2022), whereas others have shown the age of first exposure to interpersonal violence was not significantly associated with psychopathology (Dunn et al., 2017). Studies commonly assess timing effects by comparing relatively broad age groups, rarely assessing exposure at the more fine-grained measurement of individual years. A study published in 2025, however, assessed nine time points between age 4 and 18 years. They tested fluctuations in the relationship between adversity and internalising symptoms, where adverse child experiences (ACEs) including emotional abuse and parent divorce formed a composite score of adversity. Time-varying estimation models testing fluctuations revealed coefficients at age 15 years were three times that of the coefficients at age 5 (Lee et al., 2025). This emerging research demonstrates the value of a fine-grained approach to testing the timing of adversity exposure in adolescence. However, work is needed to 1) understand the effect of timing of adversity beyond age 18 years and 2) to test how adverse social experiences might relate to internalising symptoms in adolescence, during which time individuals may be particularly vulnerable (Pollmann et al., 2025; Cheng et al., 2024).

Adverse social experiences may be more prominent in adolescence. Here we define adverse social experiences as potentially traumatic events at the interpersonal level, i.e. arising from relationships with others, including peers (Pollmann et al., 2025). Emerging evidence suggests a high prevalence of adverse social experience in adolescence, with around 18-22% reporting experiencing multiple social adversities, including peer relationship problems, sibling bullying, and school issues across two UK cohorts of nearly 15,000 adolescents (Kwok & Inman et al., 2025). This is higher than the reported rate of adverse childhood experiences, which sits around 8-10% (Nweze et al., 2023). We studied two types of adverse social experiences: bullying and social exclusion. Bullying is defined as repeated abuse where the individual is not able to defend themselves (Olweus, 2013). Rates of bullying are high among adolescents: A survey of almost 300,000 participants found that 30% of 12-17-year-olds reported experiencing at least one instance of bullying in the past 30 days (Biswas et al., 2020). A 2023 meta-analysis showed that children and adolescents who

were bullied were 2.77 times more likely to suffer from depression than those who were not bullied (Ye et al., 2023). Social exclusion is considered an indirect form of victimisation and defined as being purposefully rejected or left out (Mulvey et al., 2017; Wang et al., 2010a). It can be a form of bullying; many national surveys have included feeling excluded in their questions about bullying (Charania et al., 2022). In a sample aged 11-19 years, social exclusion was found to be negatively associated with wellbeing (Arslan, 2018a). Greater internalising symptoms were found in mid-adolescents reporting social exclusion compared to non-excluded adolescents, and the socially excluded group also demonstrated heightened activation in social brain regions (including the dorsal anterior cingulate cortex, subgenual anterior cingulate cortex, and anterior insula) (Rudolph et al., 2016). Models examining timing of adversity exposure, as in ACEs research (Lee et al., 2025), need to be applied to adverse social experiences in adolescence to clarify age-specific sensitivity and to direct future prevention efforts."

**Reviewer 3, Comment 2:** Methods
- There are several aspects about the study design that remain elusive and that should be clarified for better understanding:
o It is not entirely clear to me how the reported sample size is achieved. I assume that these are participants who participated at least at one assessment. But how many participants participated at each age?
o At how many assessments did participants participate on average?
o In which calendar years were data collected?

***Participants with at least one assessment were included. We have detailed the sample sizes and missing data patterns by age (Table S2 and Figure S1). A table with the calendar years of data collection (Table S1) in the supplementary materials, and we have added information to the methods to clarify:***

***Methods, page 10:***
"Participants with internalising symptom data for at least one time point were included. Out of a possible 6 timepoints, participants in the 10-15 years sample had data for an average of 1.7 timepoints (range = 1-4) for internalising symptoms and 1.7 timepoints for bullying (range = 1-4). Participants in the 16-21 years sample had data for an average of 2.6 timepoints (range = 1-6) for internalising symptoms and 2 timepoints (range = 1-4) for social exclusion."

**Reviewer 3, Comment 3:** How are participants recruited and are they representative of the UK population or parts of the population?

***We have added information about participants representativeness to the methods:***

***Methods, page 6:***
"Over 100,000 participants have been enrolled in the Understanding Society study so far. Understanding Society was designed as a large, equal-probability sample of the general population of the UK. Ethic minority and immigrant households were oversampled for meaningful analysis. Understanding Society report close population estimates between the sample and Office for National Statistics data (Understanding Society, 2025). The University of Essex Ethics Committee has approved all data collection on Understanding Society main study, COVID-19 surveys and innovation panel waves, including asking consent for all data linkages except to health records. The full ethics statement from Understanding Society is reported in the supplementary materials (Supplementary Methods 1)."

**Reviewer 3, Comment 4:** It is not quite clear from the description how the variables were standardized. At one point it was mentioned that variables were standardized to a range of 0-100, at another point the manuscript talked about z-standardized variables. From the coefficients that are later reported I assume that it was the former, but wouldn't the latter

result in more easily interpretable estimates?

***We apologise for the lack of clarity. We rescaled variables from 0-100 to ensure they were equally weighted in the variable average, specifically, the bullying score was calculated as the mean across items after individual items were rescaled from 0-100. Z-scores were calculated only to identify outliers. For our coefficients, we have reported unstandardised estimates as sell as standardised estimates from lavaan's inbuilt "std.all" function.***

***Methods, page 9:***
"We treated z-scores greater than 5 and less than -5 as missing in order to exclude outliers due to human error as opposed to natural variation in the data (Fuhrmann et al., 2020). Z-scores were calculated only to identify outliers."

***Methods, page 12:***
"We have reported unstandardised estimates to aid meaningful interpretation of results and standardised estimates for transparency (Flora at al., 2025)."

**Reviewer 3, Comment 4:** Discussion
- There are several key limitations not mentioned that are important for conceptualising the present results. These should be addressed in the discussion or acknowledged as limitations:
o Other potential covariates that could account for confounding between the social exclusion-internalizing association.

***We have expanded in the discussion and addressed the limitation of lack of confounders:***

***Discussion, page 24:***
"Future research will need assess potential confounding carefully, including, e.g. the use of genetically informative designs. As a result, caution should be applied in the interpretation of results."

**Reviewer 3, Comment 4:** The limitations in establishing causality or directionality should be explicitly acknowledged, particularly given the cross-sectional nature of the association of interest. Also, there is evidence that internalizing symptoms are prospectively associated with future social exclusion so reverse causation is possible.

***Thank you for highlighting this important point. We agree that we cannot rule out bidirectional effects of internalising symptoms and bullying/social exclusion, therefore we have interpreted results as associations rather than implying single direction. We have also outlined this in the discussion as a limitation.***

***Example of association interpretation, page 20:***
"Higher bullying was associated with higher internalising symptoms at each year from age 10 to 15."

***Directionality limitation stated in discussion, page 24:***
"While our models allow us to, e.g. control for differences in internalising symptoms at baseline, and to account for exposure in previous years, caution must be applied here in interpreting the direction and causality of effects in the associations reported."

**Reviewer 3, Comment 4:** The potential impact of cohort effects should be discussed, given that the data were aligned by age rather than date of assessment and given the aforementioned secular changes in mental health.

***We have added this to the discussion:***

***Discussion, page 24:***
*“*Aligning data by age allowed us to model developmental patterns more precisely; however, this approach may also introduce cohort effects, as differences may reflect changes across age as well as secular changes in mental health”

Smaller comments:
**Reviewer 3, Comment 4:** The description of ethics approvals includes data collections that don’t appear directly relevant to this study. Consider referencing a source describing the broader project and focusing on the approvals relevant to the present study.

***We have shortened this section and moved the additional details for the supplementary:***

***Methods, page 6:***
“The University of Essex Ethics Committee has approved all data collection on Understanding Society main study, COVID-19 surveys and innovation panel waves, including asking consent for all data linkages except to health records. The full ethics statement from Understanding Society is reported in the supplementary materials (Supplementary Methods 1).”

**Reviewer 3, Comment 4:** Standardized coefficients should be included in the abstract for an easier interpretation.

***We have updated the abstract with standardised parameter estimates:***
**Abstract:** Adolescence (age 10-24 years) has been proposed as a second sensitive period of development with heightened sensitivity to socio-cultural processing. However, age-specific relationships between adverse social experiences and mental health have yet to be tested. In this study, we used self-reported data at yearly age intervals from age 10 to 21 years from the UK Household Longitudinal (UKHLS) Study. We investigated individual differences in how internalising symptoms change with age and whether there are differences in sensitivity to adverse social experiences (bullying and social exclusion). We captured the relationship between bullying and internalising symptoms from age 10 to 15 years (N=10,285) and social exclusion and internalising symptoms from age 16 to 21 years (N=11,623), by leveraging latent growth curve models with time-varying covariates. More bullying was associated with greater internalising symptoms, and there was evidence for age-specific sensitivity at yearly intervals from age 10-15 years (estimates = .541-.714, *ps* < .001, standardised estimates: .270-.400, *FDR corrected ps* < .05). From ages 16-21, higher feelings of social exclusion were associated with greater internalising symptoms (est = .312, $p < .001$), and the strength of this relationship remained stable over time. This evidence shows that vulnerability to adverse social experiences varies over age, and more so between age 10-15 years. The findings highlight: 1) the role of adverse social experiences in internalising symptoms with yearly precision, 2) the relevance of age-specific vulnerabilities in designing anti-bullying interventions, 3) and the importance of considering sensitivities beyond age 18 years.

**Reviewer 3, Comment 4:** The three bullying items were averaged, but one appears to be on a different scale than the other two. This approach should be clarified.
***We have clarified this calculation:***
***Methods, page 8:***
“The bullying score was calculated as the mean across items after individual items were rescaled from 0-100.”

***RESPONSE TO EDITOR:*** *Thank you for the opportunity to revise the manuscript and for the additional comments received via the Editorial Request Form. We have made the requested changes from the Editorial Request form and responded to the reviewer comments below in full. Changes are highlighted in blue in the manuscript.*

***RESPONSE TO REVIEWERS:***
REVIEWER EXPERTISE:

Reviewer #3 adolescent social development
Reviewer #4 latent growth curve modeling

REVIEWER REPORTS:

Reviewer #3 (Remarks to the Author):

Thank you for the opportunity to review the revised version of this manuscript. I appreciate that the authors were very responsive to the comments raised by me and the other reviewers. I have no other comments at this stage.

*We thank the reviewer for taking the time to assess our manuscript and are appreciate the positive review.*

Reviewer #4 (Remarks to the Author):

In this study, the authors examine the relationship between adverse social experiences, specifically bullying and social exclusion, on internalising symptoms in adolescents from age 10 to 21. It is an interesting study using a large data set from the UK Household Longitudinal Study, and the authors provided a major revision of the first draft, based on the first round of reviews. The study builds upon earlier works on timing effects of adverse childhood experiences and strives to reveal periods of heightened sensitivity for adverse social experiences. I think the study deals with a relevant topic and I was able to follow the manuscript well. The authors responeded to all questions raised by the reviewers in the first round of review. Furthermore, I think the authors do a good job in describing their analysis and the particular latent growth curve model with time-varying covariates used. Still, I have some questions, which I list below.

*We thank the reviewer for the constructive comments and careful review of the models. We have responded in full below. Revisions to the manuscripts are highlighted in blue in the document.*

1) I want to follow up on a point raised by Reviewer 2, who asked whether the time-varying covariates (TVC) bullying (age 10 to 15) and social exclusion (age 16 to 21) could also be modeled in a longitudinal model (e.g., characterized by some form of growth process or an autoregressive trajectory or both). Based on the reviewer's comment, it seems to me that the authors then included a higher lag of bullying, because they found evidence for developmental sensitivity (i.e., time-specific effects of the TVC on internalsing symptoms) in this model only. In this model, the contemporaneous relationship between bullying and internalising symptoms remained significant (i.e., lag-0-bullying), and lag-1-bullying also significantly predicted internalising symptoms except for age 15. I generally think the inclusion of lags of higher order is a good idea. However, I also wonder if bullying is characterized by a latent growth process which should be modeled, in order to separate a

between-person growth process from the within-person deviation from this process (as it is done with interalising symptoms). In particular, my question is why the authors decided to fit the latent growth model with the time-varying covariate discussed in Curran et al. (2014) which focuses on the within-person relationships between the TVC and the outcome, but omits the relationships which (may) exist at the between-level (e.g., is a linear increase in bullying over the years related to a linear increase in internalising symptoms)? Curran et al. (2014) present the latent curve model with structured residuals in the same paper to disentangle these between-person from the within-person components. I am not saying that this model should be used, but I would appreciate it if the authors would comment on why modeling bullying or social exclusion via growth curves or autoregressive trajectories is not of interest here.

*We thank the reviewer for their thoughtful consideration of the modelling choices made. Our rationale for modelling internalising, but not bullying, as a growth process is conceptual in nature: We conceptualise internalising symptoms as a latent growth factor in line with evidence showing that it is an internal, developmental process that broadly increases during adolescence (e.g., Pfeifer & Allen, 2020). In contrast, we conceptualize bullying as an episodic exogenous exposure. It perturbs trajectories of internalising symptoms, rather than co-developing with them along its own intrinsic trajectory. This is in line with theories conceptualising bullying as an external environmental stressor or adversity (e.g. Pollmann et al., 2025). We have now included this explanation in the latent growth models section of the Methods:*

*2.4.Latent growth models, page 11:*
"Bullying and social exclusion are conceptualised as episodic exogenous stressors, rather than a developmental process, and therefore modelled as time-varying covariates."

2) In the LCM-TVC by Curran et al. (2014), the time-varying covariates are also allowed to covary with the growth factors (see Figure 3 in their paper). From the figure in the current study and the exploratory supplemental materials, this does not seem to be the case here. What is the reason for not including this correlation in the model?

*Curran does include these effects but emphasizes that models need to align with theories of development (Curran et al., 2015). In our case, we are not looking to discount the effect of bullying on the overall developmental process, that would be akin to conceptualizing it as a stable between-person characteristic. This makes sense for TVCs like, e.g. drug use, but as explained above (previous comment) does not align with our conceptualization of bullying as an episodic exogenous stressor.*

3) Furthermore, the authors write on page 11, lines 288-290: "Time-varying covariates (bullying and social exclusion, in this case) allow us to examine how variables that naturally change over time influence the slope at specific points along the age trajectory." I don't think this is the case in the LCM-TVC, because the slope factor is not regressed on the TVC, or am I misunderstanding this sentence?

*Thank you for picking up this inconsistency. We have revised this sentence to reflect the model parameters:*

*2.4.Latent growth models, page 11:*
"Time-varying covariates (bullying and social exclusion, in this case) allow us to examine time-specific associations between adverse social experiences and internalising symptoms at each age, after accounting for individual differences in the internalising symptom trajectory."

4) I don't understand the final sample size of the models - are the 20% taken from the N = 10,285 and N = 11,623, respectively, which are stated on page 6, line 162? Or is this already the 80% the confirmatory analysis in the manuscript is based on?

*Apologies for the lack of clarity. We have reordered the information in this paragraph and edited for clarity:*

*2.1.Study design and cohort characteristics, page 6:*
"Data from Waves 1-12 in the UKHLS cohort was downloaded in February 2023. Different measures are used to capture social exclusion and internalising symptoms between ages 10-15 and 16-21; therefore, we fit separate models to ages 10-15 (N = 10,285) and ages 16-21 (N = 11,623). The main samples reported here consist of 80% of the full sample: We randomly selected 20% of the sample as the exploratory sample to write the analysis scripts and test model convergence, we then replicated the analysis in the confirmatory sample (remaining 80%). The approach is recommended in secondary data analysis to control for researcher degrees of freedom when pre-registration is not feasible due to model complexity (Akker et al., 2021). The confirmatory sample descriptive statistics and results are reported in this manuscript."

5) Anyway, I like that the authors split the large sample in an exploratory and a confirmatory sample! I find it interesting that in the exploratory sample age 16 to 21, the developmental sensitivty model also does seem to fit the data better, compared to the confirmatory analysis, where the regression estimates of social exclusion on internalising symptoms are stable over time. This does, in my opinion, not need to be discussed in the main text, but I wonder if the authors have also thought about this? If the (exploratory) sample is drawn at random, I would expect that the results align.

*We thank the reviewer for the observation and careful reading of our findings. We think it likely that the difference in findings between exploratory and confirmatory samples is attributable to the smaller sample size of the exploratory sample (20% of full dataset), which, combined with the substantial missing data in the 16–21-year sample, reduces statistical power to detect differences between developmental and stable sensitivity models. The chi-square difference test is sensitive to sample size, and borderline results may therefore differ across subsamples of unequal size. We consider the confirmatory sample (80%) to provide the more reliable estimate.*

Minor Points

6) On page 8, line 212, the authors refer to the GHQ-12 for measuring "internalising symptoms in the sample aged 10-15 years" in the section regarding the 16 to 21 years sample. I am not sure if this is intended or should rather be the sample aged 16 to 21?

*Thank you for picking up this typo, we have corrected to age 16-21 years:*

*2.2.2. Measures completed by age 16-21 years sample*
*"*The SDQ was only administered up to age 15 in the UKHLS cohort; therefore, we used the GHQ-12 General Health Questionnaire (Goldberg et al., 1997) to capture internalising symptoms in the sample aged 16-21 years."

7) On page 14, line 364, the estimate is presented double (est = -.811, estimate = -.811), the same happens in line 365

*Thank you for picking up these issues, we have deleted the repetition from these two sentences.*

8) In the figure note of Figure 3, the wording "confidence intervals (+/- 95%)" (line 391) is somewhat unusual, I would rather call it just 95% confidence intervals

*Thank you – we now use “95% confidence intervals”.*

9) On page 18, line 453, the authors write that "bullying at age 14 did not significantly predict bullying at age 15 years" - I think this should be internalising symptoms at age 15 instead of bullying?

*Thank you for picking this up – we have corrected to internalising symptoms.*

10) Page 24, line 612 misses a period after "levels"
*Thank you, we have added the period.*

11) Page 25, line 638 "measures of" is doubled

*Thank you, we have deleted the repetition.*
