# Supplementary material for: Greater bullying and social exclusion are linked to higher internalising symptoms from ages 10 to 21
Source: Commun Psychol. 2026 Jul 8;4:151. doi: 10.1038/s44271-026-00492-x (PMC13724274; doi:10.1038/s44271-026-00492-x)
Supplement: Supplementary file 2 [file 44271_2026_492_MOESM2_ESM.pdf]

Supplementary materials: Greater bullying and social exclusion are linked to higher internalising symptoms from ages 10 to 21

Authors: Kathryn E. Bates, Theresa Cheng, Erin Dunn, Rogier A. Kievit, Delia Fuhrmann

Supplementary Methods 1

The University of Essex Ethics Committee has approved all data collection on Understanding Society main study, COVID-19 surveys and innovation panel waves, including asking consent for all data linkages except to health records. Requesting consent for health record linkage was approved at Wave 1 by the National Research Ethics Service (NRES) Oxfordshire REC A (08/H0604/124), at BHPS Wave 18 by the NRES Royal Free Hospital & Medical School (08/H0720/60) and at Wave 4 by NRES Southampton REC A (11/SC/0274). Approval for asking consent for health record linkage and for the collection of blood and subsequent serology testing in the March 2021 wave of the COVID-19 study was obtained from London – City & East Research Ethics Committee (21/HRA/0644). Approval for the collection of biosocial data by trained nurses in Waves 2 and 3 of the main survey was obtained from the National Research Ethics Service (Understanding Society – UK Household Longitudinal Study: A Biosocial Component, Oxfordshire A REC, Reference: 10/H0604/2). The biosocial data collection at IP12 'Understanding Society Health Innovation Panel: Biomeasure and health data collection from the Innovation Panel of the UK Household Longitudinal Study' was approved by East of England – Essex Research Ethics Committee, Ref 19/EE/0146.

Table S1

UKHLS wave and dates collected for each measure at ages 10-15 years and 16-21 years. Internalising symptoms are highlighted in grey, and bullying and social exclusion are highlighted in white.

Wave (date collected)	Age 10-15 years		Age 16-21 years	
	Internalising symptoms	*Bullying*	*Internalising symptoms*	*Social exclusion*
Wave 1 (Jan 2009-March 2011)	✓	✓	✓	
Wave 2 (Jan 2010-March 2012)				
Wave 3 (Jan 2011-July 2013)	✓	✓	✓	
Wave 4 (Jan 2012-June 2014)				
Wave 5 (Jan 2013-June 2015)	✓	✓	✓	
Wave 6 (Jan 2014-May 2016)				
Wave 7 (Jan 2015-May 2017)	✓	✓	✓	
Wave 8 (Jan 2016-May 2018)				
Wave 9 (Jan 2017-May 2019)	✓	✓	✓	✓
Wave 10 (Jan 2018-May 2020)				✓
Wave 11 (Jan 2019-May 2021)	✓	✓	✓	✓
Wave 12 (Jan 2020-May 2022)				✓

Table S2

Number and percentage of missing per observed variable

Variable	Number of measurement occasions	Number of missing	Percentage missing
	10-15 years		
Internalising symptoms (Emotional problems, SDQ)			
10 years	2733	7579	73.49
11 years	2802	7510	72.83
12 years	2924	7388	71.64
13 years	2843	7469	72.43
14 years	2937	7375	71.52
15 years	2779	7533	73.05
Bullying			
10 years	2751	7561	73.32
11 years	2813	7499	72.72
12 years	2937	7375	71.52
13 years	2875	7455	72.29
14 years	2949	7363	71.40
15 years	2795	7517	72.89
	16-21 years		
Internalising symptoms (GHQ-12)			
16 years	5180	6443	55.43
17 years	5463	6160	52.99
18 years	5152	6471	55.67
19 years	4931	6692	57.58
20 years	4696	6927	59.60
21 years	4599	7024	60.43
Social exclusion			
16 years	1241	10535	90.64
17 years	1088	10382	89.32
18 years	1265	10358	89.12
19 years	1212	10411	89.57
20 years	1147	10476	90.13
21 years	1114	10509	90.42

Figure S1

Patterns of missingness for each observed variable in 10-15 years sample (A) and 16-21 years sample (B). For example, row 3 of plot A shows 812 participants have data for 6 variables, bullying and internalising symptoms (IS) at ages 10, 12, and 14 years, a further 678 participants have data for bullying and internalising symptoms at age 12 and 14, and so on. Note the columns are ordered by pattern rather than column name. Only 40 patterns are shown per plot for visual clarity.

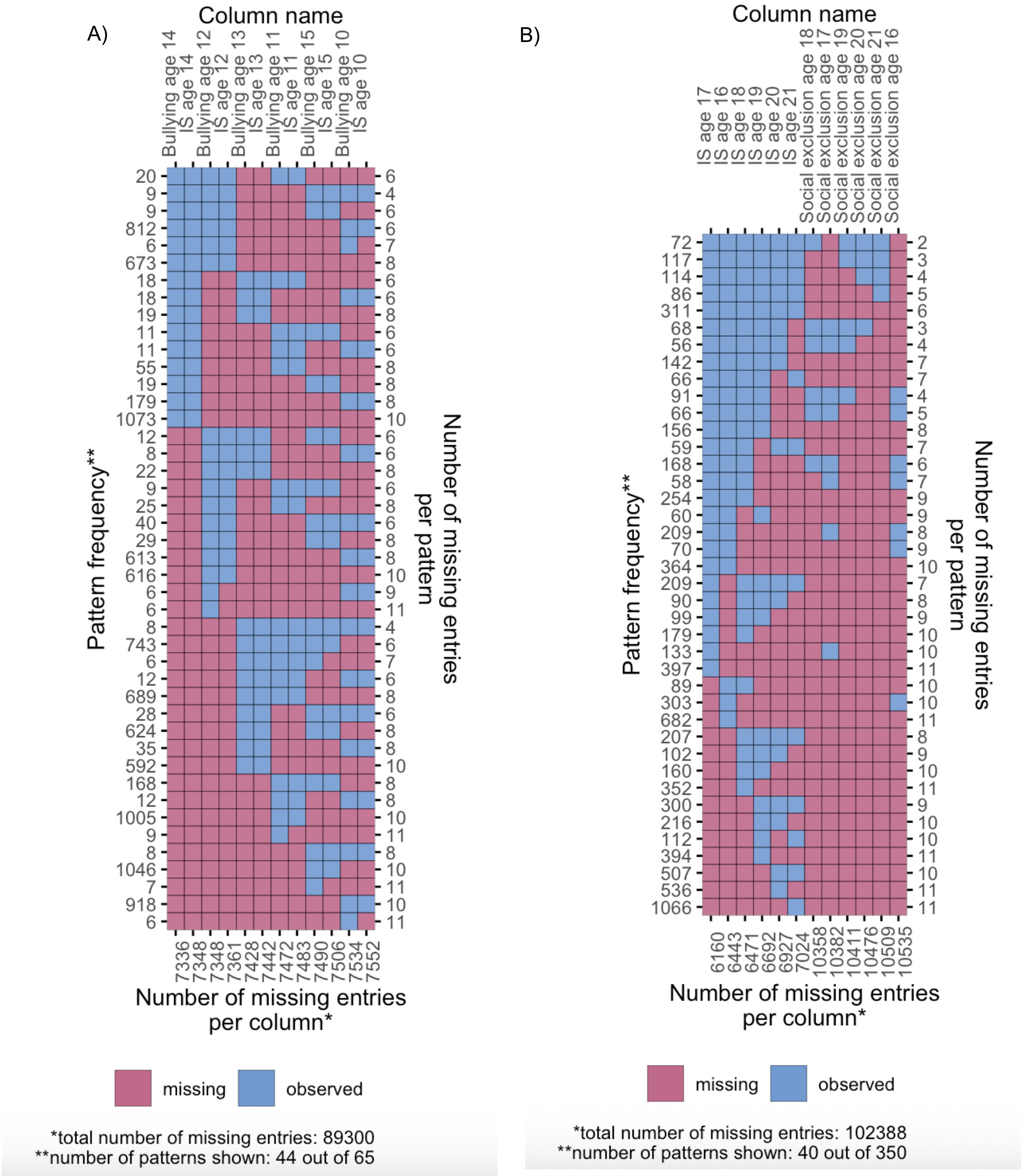

Figure S2

Correlations between SDQ Emotional problems at age 15 and GHQ-12 scores from age 16 to 21 years. Only correlations surviving Bonferroni correction for multiple comparisons (alpha level .05/12 comparisons = $p < .004$) visualised.

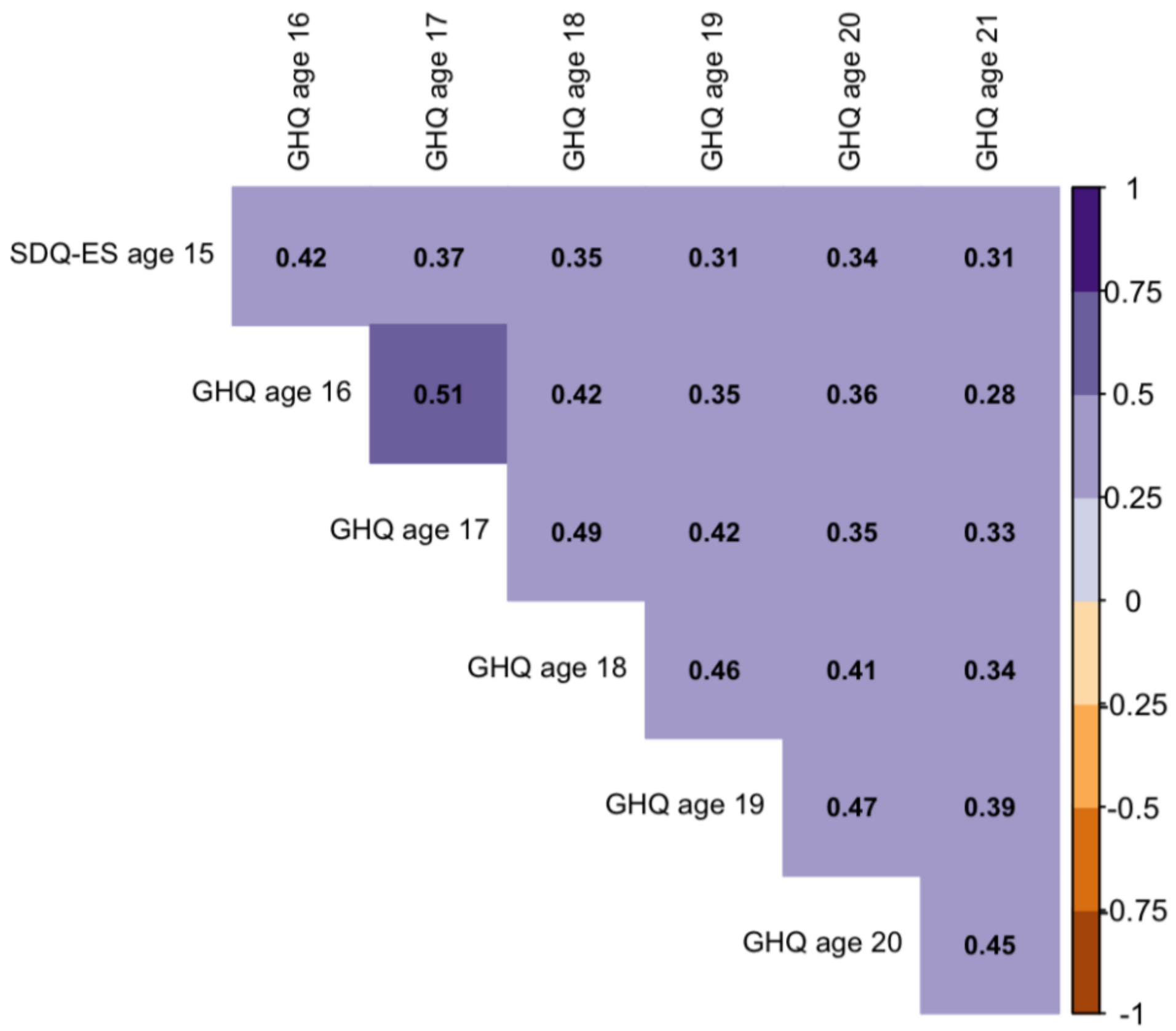

Supplementary Figure S3

Path schematic for latent growth model accounted for previous years social adversity (sensitivity analysis). The parameters “b1”, “b2”, “b3” etc. represent the previous year of social adversity, bullying in the example visualised, allowed to vary by age. The parameters labelled “b” represent previous year of social adversity constrained to be equal over age.

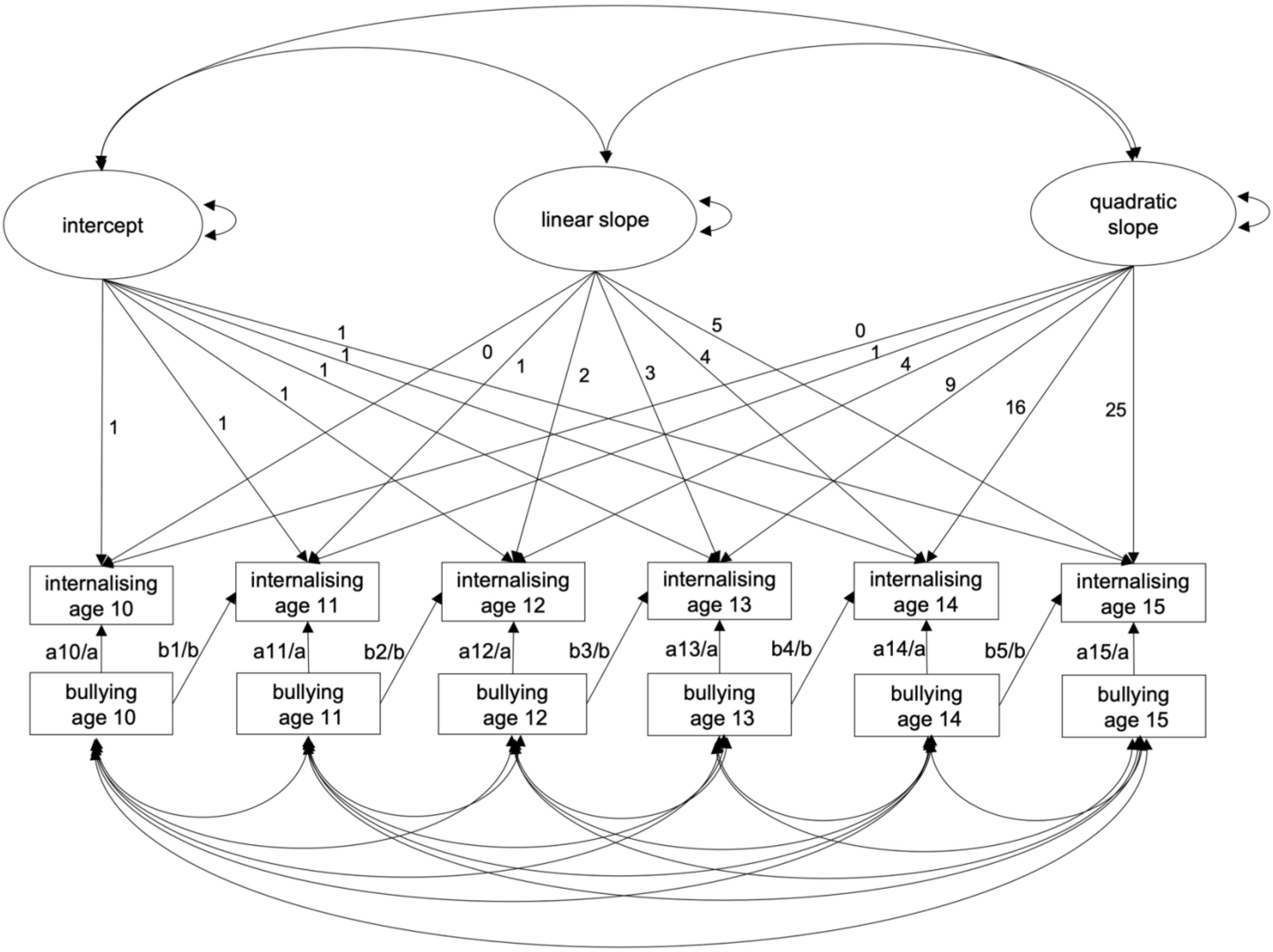

Table S3

Model fit indices for trajectories for both age 10-15 years and age 16-21 years

Model	χ^2(df)	*p* value	RMSEA (lower 90% CI, upper 90% CI)	*CFI*	SRMR
			Age 10-15 years		
Linear	69.92(16)	< .001	.018 (.014, .023)	.971	.056
Linear and quadratic	18.18(12)	.110	.007 (.000, .013)	.997	.054
			Age 16-21 years		
Linear	117.49(16)	< .001	.023 (.020, .027)	.980	.031
Linear and quadratic	51.41(12)	<.001	.017 (.012, .022)	.992	.018

Table S4

Means and standard deviations and Welch's t test results for internalising symptoms and bullying at each age between males and females. Bonferroni corrections applied to p values for 12 comparisons (.05/12 = .004), significant results after correction highlighted in bold.

**Variable**	**Age**	**Males (Mean, SD)**	**Females (Mean, SD)**	**Welch's t test: t(df), *p***
Internalising symptoms				
	10	26.55 (22.12)	29.44 (21.72)	**-3.45(2729), .00057**
	11	24.94 (21.78)	29.28 (22.06)	**-5.24(2789), < .001**
	12	23.26 (19.88)	31.33 (22.94)	**-10.14(2832), < .001**
	13	23.23 (20.69)	34.31 (24.10)	**-13.17(2801), < .001**
	14	21.84 (19.69)	37.32 (24.53)	**-18.84(2797), < .001**
	15	22.80 (20.74)	39.42 (24.37)	**-19.41(2763), < .001**
Bullying				
	10	36.84 (16.07)	34.46 (14.12)	**4.12(2725), < .001**
	11	34.84 (14.68)	32.03 (11.68)	**-5.59(2583), < .001**
	12	33.06 (13.97)	32.17 (12.37)	1.83(2912), .067
	13	32.79 (13.94)	32.10 (12.39)	1.40(2793), .161
	14	30.96 (11.28)	31.31 (11.30)	-.861(2946), .389
	15	30.81 (11.93)	29.84 (9.66)	2.33(2569), .019

Table S5

Means and standard deviations and Welch's t test results for internalising symptoms and bullying at each age between males and females. Bonferroni corrections applied to p values for 12 comparisons (.05/12 = .004), significant results after correction highlighted in bold.

Variable	Age	Males (Mean, SD)	Females (Mean, SD)	Welch's t test: t(df), *p*
Internalising symptoms				
	16	24.99 (13.63)	32.30 (16.66)	**-17.35(5137), < .001**
	17	26.34 (14.03)	33.35 (16.89)	**-16.72(5420), < .001**
	18	27.15 (14.34)	33.13 (17.15)	**-13.63(5132), < .001**
	19	27.73 (14.72)	32.62 (16.97)	**-10.84(4926), < .001**
	20	29.02 (15.42)	32.98 (16.85)	**-8.39(4636), < .001**
	21	28.83 (14.70)	33.58 (16.86)	**-10.22(4585), < .001**
Social exclusion				
	16	49.70 (21.30)	53.38 (21.85)	-2.80(1060), .005
	17	49.42 (21.21)	55.42 (23.19)	**-4.76(1232), < .001**
	18	48.66 (20.98)	55.72 (23.03)	**-5.69(1258), < .001**
	19	50.48 (21.23)	54.44 (22.64)	**-3.14(1195), < .001**
	20	50.32 (20.98)	54.74 (22.57)	**-3.43(1130), .0006**
	21	49.90 (20.11)	52.90 (22.29)	-2.36(1074), .018

Table S6

Relationship between bullying and internalising symptoms in the sample aged 10-15 years for males and females

Age (years)	Estimate	SE	95% CI		*p*	Standardised estimates
			LL	*UL*		
Males						
10	.499	.03	.43	.57	<.001	.368
11	.513	.03	.46	.57	<.001	.351
12	.529	.03	.47	.58	<.001	.368
13	.576	.03	.52	.63	<.001	.397
14	.636	.03	.58	.69	<.001	.369
15	.721	.05	.63	.82	<.001	.416
Females						
10	.613	.04	.54	.68	<.001	.399
11	.623	.03	.56	.68	<.001	.336
12	.654	.03	.59	.71	<.001	.357
13	.683	.03	.62	.74	<.001	.356
14	.720	.03	.65	.79	<.001	.327
15	768	.05	.67	.87	<.001	.312

Table S7

Results from the developmental sensitivity model accounting for bullying in the previous year

Age (years)	Effect type	Estimate	SE	95% CI		*p*	*FDR*	Standardised estimates
				lower	*upper*			
10	Age-specific	.579	.03	.52	.63	<.001	.00016	.400
11	Age-specific	.406	.08	.25	.56	<.001	.00016	.247
11	Bullying age 10	.288	.09	.11	.49	.002	.003	.201
12	Age-specific	.475	.06	.36	.59	<.001	.00016	.287
12	Bullying age 11	.327	.07	.19	.47	<.001	.00016	.199
13	Age-specific	.528	.06	.41	.65	<.001	.00016	.304
13	Bullying age 12	.319	.08	.17	.47	<.001	.00016	.184
14	Age-specific	.408	.20	.01	.80	.044	.048	.194
14	Bullying age 13	.435	.21	.03	.84	.036	.044	.240
15	Age-specific	.600	.12	.37	.82	<.001	.00016	.270
15	Bullying age 14	.227	.13	-.08	.49	.092	.092	.106

KB_Developmental sensitivity output_Exploratory sample

2025-13-06

- Info
- Outline
- Setup
  - Read in packages
  - Read in data
- Fit models for age 10-15 years sample (N = 2576)
  - Plot internalising symptoms (is) for age 10-15 years
  - Fit LGM for youth (10-15 years)
  - Fit LGMs with nonlinear slopes (basis, quad)
  - Plot predicted linear + quadratic model
  - Fit stable sensitivity model to youth 10-15
  - Fit developmental sensitivity model to youth 10-15
  - Compare stable and developmental sensitivity models
  - Plot regression coefficients for developmental sensitivity model age 10-15
- Fit models for age 10-15 years sample (N = 2905)
  - Plot internalising symptoms age 16-21 years
  - Fit LGM for older youth age 16-21
  - Fit nonlinear models to older youth (basis + quad)
  - Plot predicted linear + quadratic model
  - Fit stable sensitivity model to older youth 16-21
  - Fit developmental sensitivity model for older youth age 16-21 years
  - Compare stable and developmental sensitivity models older youth (16-21 years)
  - Plot regression coefficients for developmental sensitivity model age 16-21

Info

These are the results from the main analysis of the exploratory sample (randomly selected 20% of full sample) for the manuscript titled: “Internalising Symptoms Show Differential Developmental Sensitivity to Social Exclusion between Ages 10-21”, by Kathryn Bates, Ayla Pollmann, Rogier Kievit, and Delia Fuhrmann. The confirmatory sample results are presented in the paper. The analytical procedure is outlined below, please see Methods in the manuscript.

For queries, contact Kathryn Bates: kathryn.2.bates@kcl.ac.uk (mailto:kathryn.2.bates@kcl.ac.uk)

Outline

We investigated trajectories of internalising symptoms from age 10-15 years and from age 16-21 years in the UKHLS dataset. We then tested whether internalising symptoms were more sensitive to social exclusion/bullying at specific ages. We followed the same procedure for the age 10-15 years sample (N =

2576, 20% of full sample) and the age 16-21 years sample (N = 2905):

- Plot trajectories of internalising symptoms
- Fit linear latent growth model, and nonlinear linear growth models (basis and quadratic slopes)
- Compare model fit to identify shape of trajectories
- Fit a stable sensitivity model where the effect of social exclusion/bullying is the same across age
- Fit a developmental sensitivity model where the effect of social exclusion/bullying varies per age
- Compare model fit to find model best explaining the data
- Plot the regression coefficients

Setup

Read in packages

Read in data

```
youth_exp = read.csv("youth_exp.csv")
olderyouth_exp = read.csv("olderyouth_exp.csv")
```

Fit models for age 10-15 years sample (N = 2576)

Plot internalising symptoms (is) for age 10-15 years

```
#select vars
youth_is = youth_exp %>%
  select(pidp, is_10:is_15)

#rename for plot
youth_is = youth_is %>%
  rename("10" = is_10,
         "11" = is_11,
         "12" = is_12,
         "13" = is_13,
         "14" = is_14,
         "15" = is_15)

#make long
youth_is_long = gather(youth_is, age, is_score, "10":"15", factor_key = T)
# id as factor for individual data points
youth_is_long$pidp = as.factor(youth_is_long$pidp)

#plot
youth_traj_plot = ggplot(data=youth_is_long,aes(x=age, y=is_score)) +
        geom_point(position= "jitter", size=1, alpha=.07, colour = "#c1666b") +
        geom_line(position= "jitter", size=0.7,alpha=.07, aes(group=pidp), colour =
"#c1666b") +
        stat_smooth(aes(group=1), colour="#c1666b", method="loess", se=F, size=2, f
ormula = 'y~x') +
        theme(legend.position="none", text = element_text(size=9, family='Arial'),
        axis.text.x = element_text(angle=90, vjust=0.5)) +
        labs(x = "Age (years)", y = "Internalising symptoms (scaled from 0-100)") +
        theme_bw()
```

```
## Warning: Using `size` aesthetic for lines was deprecated in ggplot2 3.4.0.
## ℹ Please use `linewidth` instead.
## This warning is displayed once every 8 hours.
## Call `lifecycle::last_lifecycle_warnings()` to see where this warning was
## generated.
```

```
#view plot
youth_traj_plot
```

```
## Warning: Removed 11230 rows containing non-finite outside the scale range
## (`stat_smooth()`).
```

```
## Warning: Removed 11230 rows containing missing values or values outside the scal
e range
## (`geom_point()`).
```

```
## Warning: Removed 9398 rows containing missing values or values outside the scale
range
## (`geom_line()`).
```

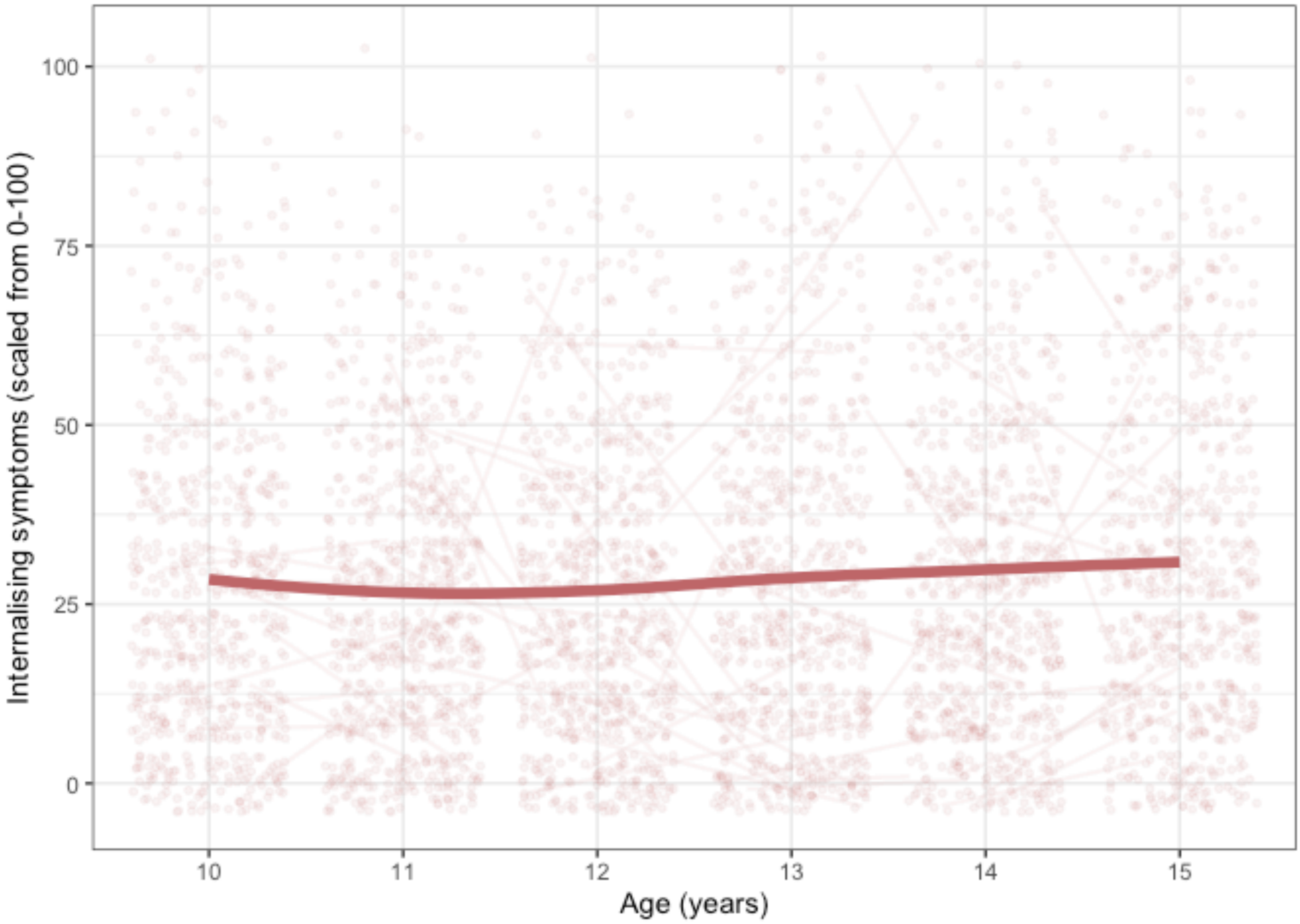

Fit LGM for youth (10-15 years)

```
#define linear model
lgm_linear<-'

#intercept
int =~ 1*is_10 + 1*is_11 + 1*is_12 + 1*is_13 + 1*is_14 + 1*is_15

#slope
slope =~ 0*is_10 + 1*is_11 + 2*is_12 + 3*is_13 + 4*is_14 + 5*is_15

#covariance
int ~~ slope

'

#fit model to youth_exp data
linearLGM_youth1015 <- growth(lgm_linear,
                              data=youth_exp,
                              estimator='mlr',
                              missing='fiml.x',
                              fixed.x = F)
```

```
## Warning: lavaan->lav_data_full():
##    some cases are empty and will be ignored: 72 84 269 333 436 545 648 673
##    784 896 1098 1241 1999 2127 2188.
```

```
## Warning: lavaan->lav_data_full():
##    due to missing values, some pairwise combinations have less than 10%
##    coverage; use lavInspect(fit, "coverage") to investigate.
```

```
## Warning: lavaan->lav_mvnorm_missing_h1_estimate_moments():
##    Maximum number of iterations reached when computing the sample moments
##    using EM; use the em.h1.iter.max= argument to increase the number of
##    iterations
```

```
# check fit
fit.indices = (c("chisq", "df", "pvalue", "cfi", "rmsea", "rmsea.ci.lower", "rmsea.
ci.upper", "srmr"))
fitMeasures(linearLGM_youth1015, fit.indices)
```

```
##          chisq             df         pvalue            cfi          rmsea
##         35.795         16.000          0.003          0.961          0.022
## rmsea.ci.lower rmsea.ci.upper           srmr
##          0.012          0.032          0.090
```

```
#view model
summary(linearLGM_youth1015,fit.measures=T,standardized=T)
```

```
## lavaan 0.6-20.2283 ended normally after 110 iterations
##
##   Estimator                                         ML
##   Optimization method                           NLMINB
##   Number of model parameters                        11
##
##                                                   Used       Total
##   Number of observations                          2561        2576
##   Number of missing patterns                        34
##
## Model Test User Model:
##                                               Standard      Scaled
##   Test Statistic                                35.795      42.279
##   Degrees of freedom                                16          16
##   P-value (Chi-square)                           0.003       0.000
##   Scaling correction factor                                  0.847
##     Yuan-Bentler correction (Mplus variant)
##
## Model Test Baseline Model:
##
##   Test statistic                               522.221     577.252
##   Degrees of freedom                                15          15
##   P-value                                        0.000       0.000
##   Scaling correction factor                                  0.905
##
## User Model versus Baseline Model:
##
##   Comparative Fit Index (CFI)                    0.961       0.953
##   Tucker-Lewis Index (TLI)                       0.963       0.956
##
##   Robust Comparative Fit Index (CFI)                         1.000
##   Robust Tucker-Lewis Index (TLI)                            4.555
##
## Loglikelihood and Information Criteria:
##
##   Loglikelihood user model (H0)             -18856.023  -18856.023
##   Scaling correction factor                                  1.056
##       for the MLR correction
##   Loglikelihood unrestricted model (H1)     -18838.125  -18838.125
##   Scaling correction factor                                  0.932
##       for the MLR correction
##
##   Akaike (AIC)                               37734.045   37734.045
##   Bayesian (BIC)                             37798.375   37798.375
##   Sample-size adjusted Bayesian (SABIC)      37763.425   37763.425
##
```

```
## Root Mean Square Error of Approximation:
##
##   RMSEA                                         0.022       0.025
##   90 Percent confidence interval - lower        0.012       0.015
##   90 Percent confidence interval - upper        0.032       0.036
##   P-value H_0: RMSEA <= 0.050                   1.000       1.000
##   P-value H_0: RMSEA >= 0.080                   0.000       0.000
##
##   Robust RMSEA                                              0.000
##   90 Percent confidence interval - lower                    0.000
##   90 Percent confidence interval - upper                    0.000
##   P-value H_0: Robust RMSEA <= 0.050                        0.975
##   P-value H_0: Robust RMSEA >= 0.080                        0.021
##
## Standardized Root Mean Square Residual:
##
##   SRMR                                          0.090       0.090
##
## Parameter Estimates:
##
##   Standard errors                             Sandwich
##   Information bread                           Observed
##   Observed information based on                Hessian
##
## Latent Variables:
##                    Estimate  Std.Err  z-value  P(>|z|)   Std.lv  Std.all
##   int =~
##     is_10             1.000                               16.839    0.740
##     is_11             1.000                               16.839    0.814
##     is_12             1.000                               16.839    0.809
##     is_13             1.000                               16.839    0.740
##     is_14             1.000                               16.839    0.739
##     is_15             1.000                               16.839    0.729
##   slope =~
##     is_10             0.000                                0.000    0.000
##     is_11             1.000                                4.924    0.238
##     is_12             2.000                                9.847    0.473
##     is_13             3.000                               14.771    0.649
##     is_14             4.000                               19.694    0.864
##     is_15             5.000                               24.618    1.065
##
## Covariances:
##                    Estimate  Std.Err  z-value  P(>|z|)   Std.lv  Std.all
##   int ~~
##     slope           -41.045   11.836   -3.468    0.001   -0.495   -0.495
##
## Intercepts:
##                    Estimate  Std.Err  z-value  P(>|z|)   Std.lv  Std.all
##     int              25.859    0.587   44.039    0.000    1.536    1.536
##     slope             0.953    0.184    5.178    0.000    0.194    0.194
##
```

```
## Variances:
##                   Estimate  Std.Err  z-value  P(>|z|)   Std.lv  Std.all
##    .is_10          234.434   42.678    5.493    0.000  234.434    0.453
##    .is_11          201.950   29.151    6.928    0.000  201.950    0.472
##    .is_12          216.929   17.150   12.649    0.000  216.929    0.501
##    .is_13          262.537   21.016   12.492    0.000  262.537    0.507
##    .is_14          176.075   25.087    7.019    0.000  176.075    0.339
##    .is_15           54.788   32.906    1.665    0.096   54.788    0.103
##     int            283.552   39.833    7.119    0.000    1.000    1.000
##     slope           24.241    4.133    5.865    0.000    1.000    1.000
```

Fit LGMs with nonlinear slopes (basis, quad)

```
#define basis model
lgm_basis <-'

#intercept
int =~ 1*is_10 + 1*is_11 + 1*is_12 + 1*is_13 + 1*is_14 + 1*is_15

#slope
basis_slope =~ 0*is_10 + is_11 + is_12 + is_13 + is_14 + 1*is_15

#covariance
int ~~ basis_slope

'

#fit model
basisLGMyouth_1015 <-growth(lgm_basis,
                            data=youth_exp,
                            estimator='mlr',
                            missing='fiml.x',
                            fixed.x = F)
```

```
## Warning: lavaan->lav_data_full():
##    some cases are empty and will be ignored: 72 84 269 333 436 545 648 673
##    784 896 1098 1241 1999 2127 2188.
```

```
## Warning: lavaan->lav_data_full():
##    due to missing values, some pairwise combinations have less than 10%
##    coverage; use lavInspect(fit, "coverage") to investigate.
```

```
## Warning: lavaan->lav_mvnorm_missing_h1_estimate_moments():
##    Maximum number of iterations reached when computing the sample moments
##    using EM; use the em.h1.iter.max= argument to increase the number of
##    iterations
```

```
#view fit
fitMeasures(basisLGMyouth_1015, fit.indices)
```

```
##          chisq             df         pvalue            cfi          rmsea
##         26.801         12.000          0.008          0.971          0.022
## rmsea.ci.lower rmsea.ci.upper           srmr
##          0.011          0.033          0.086
```

```
#define quad model
lgm_quad_train <-'

#intercept
int =~ 1*is_10 + 1*is_11 + 1*is_12 + 1*is_13 + 1*is_14 + 1*is_15

#slopes
linear_slope =~ 0*is_10 + 1*is_11 + 2*is_12 + 3*is_13 + 4*is_14 + 5*is_15
quad_slope =~ 0*is_10 + 1*is_11 + 4*is_12 + 9*is_13 + 16*is_14 + 25*is_15

#covariances
int ~~ linear_slope
int ~~ quad_slope

'

#fit model
quadLGMyouth_1015 <-growth(lgm_quad_train,
                           data=youth_exp,
                           estimator='mlr',
                           missing='fiml.x',
                           fixed.x = F)
```

```
## Warning: lavaan->lav_data_full():
##    some cases are empty and will be ignored: 72 84 269 333 436 545 648 673
##    784 896 1098 1241 1999 2127 2188.
```

```
## Warning: lavaan->lav_data_full():
##    due to missing values, some pairwise combinations have less than 10%
##    coverage; use lavInspect(fit, "coverage") to investigate.
```

```
## Warning: lavaan->lav_mvnorm_missing_h1_estimate_moments():
##    Maximum number of iterations reached when computing the sample moments
##    using EM; use the em.h1.iter.max= argument to increase the number of
##    iterations
```

```
## Warning: lavaan->lav_object_post_check():
##    some estimated ov variances are negative
```

```
#view fit
fitMeasures(linearLGM_youth1015, fit.indices)
```

```
##          chisq             df         pvalue            cfi          rmsea
##         35.795         16.000          0.003          0.961          0.022
## rmsea.ci.lower rmsea.ci.upper           srmr
##          0.012          0.032          0.090
```

```
fitMeasures(quadLGMyouth_1015, fit.indices)
```

```
##          chisq             df         pvalue            cfi          rmsea
##         19.208         12.000          0.084          0.986          0.015
## rmsea.ci.lower rmsea.ci.upper           srmr
##          0.000          0.028          0.106
```

```
#compare linear and non linear
lavTestLRT(linearLGM_youth1015, basisLGMyouth_1015) # no significant difference
```

```
##
## Scaled Chi-Squared Difference Test (method = "satorra.bentler.2001")
##
## lavaan->lavTestLRT():
##    lavaan NOTE: The "Chisq" column contains standard test statistics, not the
##    robust test that should be reported per model. A robust difference test is
##    a function of two standard (not robust) statistics.
##                     Df   AIC   BIC  Chisq Chisq diff Df diff Pr(>Chisq)
## basisLGMyouth_1015  12 37733 37821 26.801
## linearLGM_youth1015 16 37734 37798 35.795     6.7727       4     0.1484
```

```
lavTestLRT(linearLGM_youth1015, quadLGMyouth_1015) # quad fit better than linear
```

```
##
## Scaled Chi-Squared Difference Test (method = "satorra.bentler.2001")
##
## lavaan->lavTestLRT():
##    lavaan NOTE: The "Chisq" column contains standard test statistics, not the
##    robust test that should be reported per model. A robust difference test is
##    a function of two standard (not robust) statistics.
##                     Df   AIC   BIC  Chisq Chisq diff Df diff Pr(>Chisq)
## quadLGMyouth_1015   12 37725 37813 19.208
## linearLGM_youth1015 16 37734 37798 35.795     11.662       4    0.02005 *
## ---
## Signif. codes:  0 '***' 0.001 '**' 0.01 '*' 0.05 '.' 0.1 ' ' 1
```

```
#view model
summary(quadLGMyouth_1015,fit.measures=T,standardized=T)
```

```
## lavaan 0.6-20.2283 ended normally after 173 iterations
##
##   Estimator                                         ML
##   Optimization method                           NLMINB
##   Number of model parameters                        15
##
##                                                   Used       Total
##   Number of observations                          2561        2576
##   Number of missing patterns                        34
##
## Model Test User Model:
##                                               Standard      Scaled
##   Test Statistic                                19.208      29.337
##   Degrees of freedom                                12          12
##   P-value (Chi-square)                           0.084       0.004
##   Scaling correction factor                                  0.655
##     Yuan-Bentler correction (Mplus variant)
##
## Model Test Baseline Model:
##
##   Test statistic                               522.221     577.252
##   Degrees of freedom                                15          15
##   P-value                                        0.000       0.000
##   Scaling correction factor                                  0.905
##
## User Model versus Baseline Model:
##
##   Comparative Fit Index (CFI)                    0.986       0.969
##   Tucker-Lewis Index (TLI)                       0.982       0.961
##
##   Robust Comparative Fit Index (CFI)                         1.000
##   Robust Tucker-Lewis Index (TLI)                          829.696
```

```
##
## Loglikelihood and Information Criteria:
##
##   Loglikelihood user model (H0)            -18847.729  -18847.729
##   Scaling correction factor                                 1.154
##       for the MLR correction
##   Loglikelihood unrestricted model (H1)    -18838.125  -18838.125
##   Scaling correction factor                                 0.932
##       for the MLR correction
##
##   Akaike (AIC)                              37725.459   37725.459
##   Bayesian (BIC)                            37813.181   37813.181
##   Sample-size adjusted Bayesian (SABIC)     37765.522   37765.522
##
## Root Mean Square Error of Approximation:
##
##   RMSEA                                         0.015       0.024
##   90 Percent confidence interval - lower        0.000       0.010
##   90 Percent confidence interval - upper        0.028       0.037
##   P-value H_0: RMSEA <= 0.050                   1.000       1.000
##   P-value H_0: RMSEA >= 0.080                   0.000       0.000
##
##   Robust RMSEA                                              0.000
##   90 Percent confidence interval - lower                    0.000
##   90 Percent confidence interval - upper                    0.000
##   P-value H_0: Robust RMSEA <= 0.050                        1.000
##   P-value H_0: Robust RMSEA >= 0.080                        0.000
##
## Standardized Root Mean Square Residual:
##
##   SRMR                                          0.106       0.106
##
## Parameter Estimates:
##
##   Standard errors                            Sandwich
##   Information bread                          Observed
##   Observed information based on               Hessian
##
## Latent Variables:
##                    Estimate  Std.Err  z-value  P(>|z|)   Std.lv  Std.all
##   int =~
##     is_10             1.000                               26.016    1.152
##     is_11             1.000                               26.016    1.255
##     is_12             1.000                               26.016    1.239
##     is_13             1.000                               26.016    1.154
##     is_14             1.000                               26.016    1.129
##     is_15             1.000                               26.016    1.130
##   linear_slope =~
##     is_10             0.000                                0.000    0.000
##     is_11             1.000                               15.779    0.761
##     is_12             2.000                               31.558    1.503
```

```
##     is_13              3.000                                   47.338    2.101
##     is_14              4.000                                   63.117    2.739
##     is_15              5.000                                   78.896    3.427
##   quad_slope =~
##     is_10              0.000                                    0.000    0.000
##     is_11              1.000                                    2.514    0.121
##     is_12              4.000                                   10.057    0.479
##     is_13              9.000                                   22.628    1.004
##     is_14             16.000                                   40.228    1.746
##     is_15             25.000                                   62.856    2.731
##
## Covariances:
##                    Estimate  Std.Err  z-value  P(>|z|)   Std.lv  Std.all
##   int ~~
##     linear_slope   -320.604  162.052   -1.978    0.048   -0.781   -0.781
##     quad_slope       43.614   25.976    1.679    0.093    0.667    0.667
##   linear_slope ~~
##     quad_slope      -37.051   22.599   -1.639    0.101   -0.934   -0.934
##
## Intercepts:
##                    Estimate  Std.Err  z-value  P(>|z|)   Std.lv  Std.all
##     int              27.608    0.723   38.172    0.000    1.061    1.061
##     linear_slope     -1.313    0.591   -2.222    0.026   -0.083   -0.083
##     quad_slope        0.436    0.109    3.994    0.000    0.173    0.173
##
## Variances:
##                    Estimate  Std.Err  z-value  P(>|z|)   Std.lv  Std.all
##    .is_10          -166.591  224.263   -0.743    0.458 -166.591   -0.326
##    .is_11           125.890   46.515    2.706    0.007  125.890    0.293
##    .is_12           193.289   26.651    7.253    0.000  193.289    0.438
##    .is_13           217.491   35.876    6.062    0.000  217.491    0.428
##    .is_14           163.816   24.834    6.596    0.000  163.816    0.309
##    .is_15           -34.198   92.770   -0.369    0.712  -34.198   -0.065
##     int             676.833  224.739    3.012    0.003    1.000    1.000
##     linear_slope    248.985  132.360    1.881    0.060    1.000    1.000
##     quad_slope        6.321    4.036    1.566    0.117    1.000    1.000
```

Plot predicted linear + quadratic model

```
#save parameter estimates
params <- parameterEstimates(quadLGMyouth_1015)

# pull out intercept and slope
intercept_mean <- params$est[params$lhs == "int" & params$op == "~1"]
slope1_mean <- params$est[params$lhs == "linear_slope" & params$op == "~1"]
slope2_mean <- params$est[params$lhs == "quad_slope" & params$op == "~1"]

# define time points
time <- 0:5

# compute predicted
predicted_values <- intercept_mean + slope1_mean * time + slope2_mean * time^2

# plot predicted trajectory
plot(time, predicted_values, type = "b", pch = 19, col = "#c1666b",
     xlab = "Time", ylab = "Predicted Value",
     main = "Predicted LGM Trajectory (Linear + Quadratic)")
```

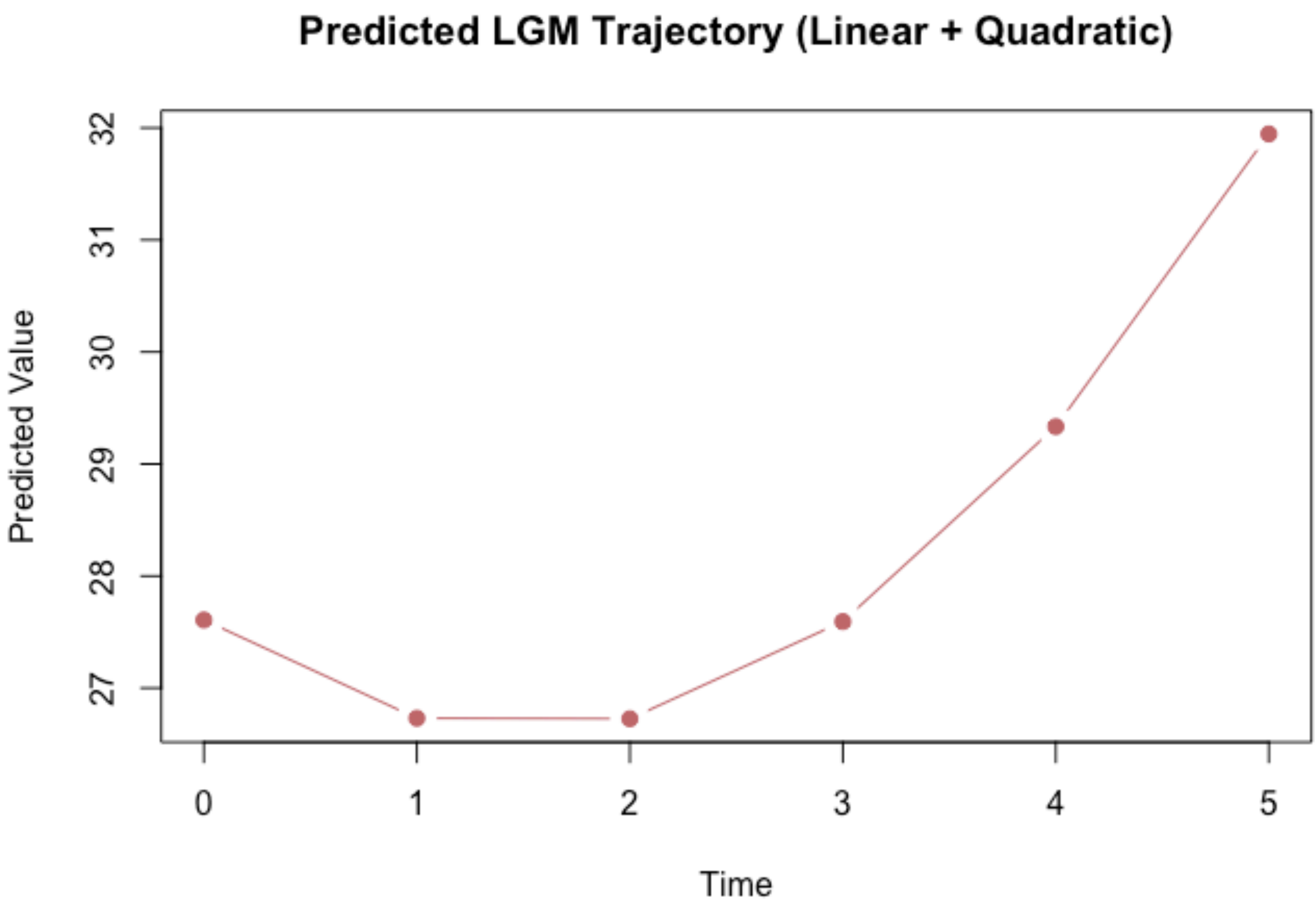

Fit stable sensitivity model to youth 10-15

Note: The model did not converge including age 11 and 15 due to limited repeated measures across internalising symptoms and bullying. This model includes age 10-15 for internalising symptoms and age 10, 12, 13 and 14 for bullying

```
# define model
lgm_stable_1015<-'

#intercept
int   =~  1*is_10 + 1*is_11 + 1*is_12 + 1*is_13 + 1*is_14 + 1*is_15

#slopes
quad_slope =~ 0*is_10 + 1*is_11 + 4*is_12 + 9*is_13 + 16*is_14 + 25*is_15
linear_slope =~ 0*is_10 + 1*is_11 + 2*is_12 + 3*is_13 + 4*is_14 + 5*is_15

#covariance between intercept and slopes
int ~~ quad_slope
int ~~ linear_slope

#regression parameters - stable sensitivity across age
is_10 ~ stablesens*bully_10
is_12 ~ stablesens*bully_12
is_13 ~ stablesens*bully_13
is_14 ~ stablesens*bully_14

#bullying intercepts
bully_10 ~ 1
bully_12 ~ 1
bully_13 ~ 1
bully_14 ~ 1

#covariances in bullying across age
bully_10 ~~ bully_12
bully_10 ~~ bully_13
bully_10 ~~ bully_14

bully_12 ~~ bully_13
bully_12 ~~ bully_14

bully_13 ~~ bully_14

'

#fit model
growth_model_stable1015<-growth(lgm_stable_1015,
                                data=youth_exp,
                                estimator='mlr',
                                missing='fiml.x',
                                fixed.x=FALSE)
```

```
## Warning: lavaan->lav_data_full():
##    some cases are empty and will be ignored: 72 333 648 1241 2127.
```

```
## Warning: lavaan->lav_data_full():
##    due to missing values, some pairwise combinations have less than 10%
##    coverage; use lavInspect(fit, "coverage") to investigate.
```

```
## Warning: lavaan->lav_mvnorm_missing_h1_estimate_moments():
##    Maximum number of iterations reached when computing the sample moments
##    using EM; use the em.h1.iter.max= argument to increase the number of
##    iterations
```

```
## Warning: lavaan->lav_object_post_check():
##    some estimated lv variances are negative
```

```
#view fit
fitMeasures(growth_model_stable1015, fit.indices)
```

```
##          chisq             df         pvalue            cfi          rmsea
##        255.515         35.000          0.000          0.801          0.050
## rmsea.ci.lower rmsea.ci.upper           srmr
##          0.044          0.055          0.137
```

```
#view model
summary(growth_model_stable1015,fit.measures=T,standardized=T,rsquare=T)
```

```
## lavaan 0.6-20.2283 ended normally after 269 iterations
##
##   Estimator                                         ML
##   Optimization method                           NLMINB
##   Number of model parameters                        33
##   Number of equality constraints                     3
##
##                                                   Used       Total
##   Number of observations                          2571        2576
##   Number of missing patterns                        44
##
## Model Test User Model:
##                                               Standard      Scaled
##   Test Statistic                               255.515     214.056
##   Degrees of freedom                                35          35
##   P-value (Chi-square)                           0.000       0.000
##   Scaling correction factor                                  1.194
##     Yuan-Bentler correction (Mplus variant)
##
```

```
## Model Test Baseline Model:
##
##   Test statistic                              1152.043     920.663
##   Degrees of freedom                                45          45
##   P-value                                        0.000       0.000
##   Scaling correction factor                                  1.251
##
## User Model versus Baseline Model:
##
##   Comparative Fit Index (CFI)                    0.801       0.796
##   Tucker-Lewis Index (TLI)                       0.744       0.737
##
##   Robust Comparative Fit Index (CFI)                         1.000
##   Robust Tucker-Lewis Index (TLI)                           -3.201
##
## Loglikelihood and Information Criteria:
##
##   Loglikelihood user model (H0)             -30120.583  -30120.583
##   Scaling correction factor                                  1.301
##       for the MLR correction
##   Loglikelihood unrestricted model (H1)     -29992.826  -29992.826
##   Scaling correction factor                                  1.303
##       for the MLR correction
##
##   Akaike (AIC)                               60301.166   60301.166
##   Bayesian (BIC)                             60476.728   60476.728
##   Sample-size adjusted Bayesian (SABIC)      60381.409   60381.409
##
## Root Mean Square Error of Approximation:
##
##   RMSEA                                          0.050       0.045
##   90 Percent confidence interval - lower         0.044       0.039
##   90 Percent confidence interval - upper         0.055       0.050
##   P-value H_0: RMSEA <= 0.050                    0.545       0.952
##   P-value H_0: RMSEA >= 0.080                    0.000       0.000
##
##   Robust RMSEA                                               0.000
##   90 Percent confidence interval - lower                     0.000
##   90 Percent confidence interval - upper                     0.000
##   P-value H_0: Robust RMSEA <= 0.050                         1.000
##   P-value H_0: Robust RMSEA >= 0.080                         0.000
##
## Standardized Root Mean Square Residual:
##
##   SRMR                                           0.137       0.137
##
## Parameter Estimates:
##
##   Standard errors                             Sandwich
##   Information bread                           Observed
##   Observed information based on                Hessian
```

```
##
## Latent Variables:
##                    Estimate  Std.Err  z-value  P(>|z|)   Std.lv  Std.all
##   int =~
##     is_10             1.000                               11.260    0.510
##     is_11             1.000                               11.260    0.531
##     is_12             1.000                               11.260    0.558
##     is_13             1.000                               11.260    0.507
##     is_14             1.000                               11.260    0.505
##     is_15             1.000                               11.260    0.490
##   quad_slope =~
##     is_10             0.000                                   NA       NA
##     is_11             1.000                                   NA       NA
##     is_12             4.000                                   NA       NA
##     is_13             9.000                                   NA       NA
##     is_14            16.000                                   NA       NA
##     is_15            25.000                                   NA       NA
##   linear_slope =~
##     is_10             0.000                                   NA       NA
##     is_11             1.000                                   NA       NA
##     is_12             2.000                                   NA       NA
##     is_13             3.000                                   NA       NA
##     is_14             4.000                                   NA       NA
##     is_15             5.000                                   NA       NA
##
## Regressions:
##                    Estimate  Std.Err  z-value  P(>|z|)   Std.lv  Std.all
##   is_10 ~
##     blly_10 (stbl)    0.226    0.020   11.425    0.000    0.226    0.149
##   is_12 ~
##     blly_12 (stbl)    0.226    0.020   11.425    0.000    0.226    0.154
##   is_13 ~
##     blly_13 (stbl)    0.226    0.020   11.425    0.000    0.226    0.128
##   is_14 ~
##     blly_14 (stbl)    0.226    0.020   11.425    0.000    0.226    0.125
##
## Covariances:
##                    Estimate  Std.Err  z-value  P(>|z|)   Std.lv  Std.all
##   int ~~
##     quad_slope      -14.985   32.377   -0.463    0.643   -0.919   -0.919
##     linear_slope     56.597  203.265    0.278    0.781    0.740    0.740
##   bully_10 ~~
##     bully_12         75.805   13.113    5.781    0.000   75.805    0.380
##     bully_13         63.667   52.393    1.215    0.224   63.667    0.350
##     bully_14         62.048   11.569    5.363    0.000   62.048    0.347
##   bully_12 ~~
##     bully_13        113.227   31.229    3.626    0.000  113.227    0.656
##     bully_14         87.543   12.336    7.097    0.000   87.543    0.516
##   bully_13 ~~
##     bully_14         35.638   40.011    0.891    0.373   35.638    0.230
##   quad_slope ~~
```

```
##     linear_slope     11.658   27.775    0.420    0.675    1.185    1.185
##
## Intercepts:
##                    Estimate  Std.Err  z-value  P(>|z|)   Std.lv  Std.all
##     bully_10         34.600    0.547   63.253    0.000   34.600    2.383
##     bully_12         33.014    0.482   68.433    0.000   33.014    2.400
##     bully_13         32.237    0.460   70.038    0.000   32.237    2.571
##     bully_14         31.219    0.413   75.518    0.000   31.219    2.532
##     int              23.868    0.823   28.988    0.000    2.120    2.120
##     quad_slope        1.026    0.128    8.041    0.000       NA       NA
##     linear_slope     -3.929    0.686   -5.726    0.000       NA       NA
##
## Variances:
##                    Estimate  Std.Err  z-value  P(>|z|)   Std.lv  Std.all
##    .is_10           350.551  286.652    1.223    0.221  350.551    0.718
##    .is_11           265.170   63.249    4.192    0.000  265.170    0.589
##    .is_12           196.128   27.678    7.086    0.000  196.128    0.482
##    .is_13           244.124   35.756    6.828    0.000  244.124    0.495
##    .is_14           172.184   27.977    6.155    0.000  172.184    0.347
##    .is_15           135.321  127.362    1.062    0.288  135.321    0.256
##     bully_10        210.822   17.528   12.028    0.000  210.822    1.000
##     bully_12        189.165   16.408   11.529    0.000  189.165    1.000
##     bully_13        157.251   14.511   10.837    0.000  157.251    1.000
##     bully_14        152.030   15.543    9.781    0.000  152.030    1.000
##     int             126.789  285.695    0.444    0.657    1.000    1.000
##     quad_slope       -2.096    4.971   -0.422    0.673       NA       NA
##     linear_slope    -46.196  163.220   -0.283    0.777       NA       NA
##
## R-Square:
##                    Estimate
##     is_10             0.282
##     is_11             0.411
##     is_12             0.518
##     is_13             0.505
##     is_14             0.653
##     is_15             0.744
```

Fit developmental sensitivity model to youth 10-15

Note: The model did not converge including age 11 and 15 due to limited repeated measures, this model includes age 10-15 for internalising symptoms (is) and age 10, 12, 13 and 14 for bullying

```
#define model
lgm_devel_1015<-'

#intercepts
int =~ 1*is_10 + 1*is_11 + 1*is_12 + 1*is_13 + 1*is_14 + 1*is_15

#slopes
quad_slope =~ 0*is_10 + 1*is_11 + 4*is_12 + 9*is_13 + 16*is_14 + 25*is_15
linear_slope =~ 0*is_10 + 1*is_11 + 2*is_12 + 3*is_13 + 4*is_14 + 5*is_15

#covariances
int ~~ linear_slope
int ~~ quad_slope

#regression parameters - developmental sensitivity
is_10 ~ devsens_10*bully_10
is_12 ~ devsens_12*bully_12
is_13 ~ devsens_13*bully_13
is_14 ~ devsens_14*bully_14

#bullying intercepts
bully_10 ~ 1
bully_12 ~ 1
bully_13 ~ 1
bully_14 ~ 1

#covariances in bullying across age
bully_10 ~~ bully_12
bully_10 ~~ bully_13
bully_10 ~~ bully_14

bully_12 ~~ bully_13
bully_12 ~~ bully_14

bully_13 ~~ bully_14

'

#fit model
growth_model_devel1015<-growth(lgm_devel_1015,
                               data=youth_exp,
                               estimator='mlr',
                               missing='fiml.x',
                               fixed.x=FALSE)
```

```
## Warning: lavaan->lav_data_full():
##    some cases are empty and will be ignored: 72 333 648 1241 2127.
```

```
## Warning: lavaan->lav_data_full():
##    due to missing values, some pairwise combinations have less than 10%
##    coverage; use lavInspect(fit, "coverage") to investigate.
```

```
## Warning: lavaan->lav_mvnorm_missing_h1_estimate_moments():
##    Maximum number of iterations reached when computing the sample moments
##    using EM; use the em.h1.iter.max= argument to increase the number of
##    iterations
```

```
## Warning: lavaan->lav_object_post_check():
##    some estimated lv variances are negative
```

```
#iew fit
fitMeasures(growth_model_devel1015, fit.indices)
```

```
##          chisq             df         pvalue            cfi          rmsea
##        243.052         32.000          0.000          0.809          0.051
## rmsea.ci.lower rmsea.ci.upper           srmr
##          0.045          0.057          0.134
```

Compare stable and developmental sensitivity models

```
#compare models
lavTestLRT(growth_model_devel1015, growth_model_stable1015) # devel model fits bett
er
```

```
##
## Scaled Chi-Squared Difference Test (method = "satorra.bentler.2001")
##
## lavaan->lavTestLRT():
##    lavaan NOTE: The "Chisq" column contains standard test statistics, not the
##    robust test that should be reported per model. A robust difference test is
##    a function of two standard (not robust) statistics.
##                         Df   AIC   BIC  Chisq Chisq diff Df diff Pr(>Chisq)
## growth_model_devel1015  32 60295 60488 243.05
## growth_model_stable1015 35 60301 60477 255.51     10.302       3    0.01617 *
## ---
## Signif. codes:  0 '***' 0.001 '**' 0.01 '*' 0.05 '.' 0.1 ' ' 1
```

```
#view devel model
summary(growth_model_devel1015,fit.measures=T,standardized=T,rsquare=T)
```

```
## lavaan 0.6-20.2283 ended normally after 270 iterations
##
```

```
##   Estimator                                         ML
##   Optimization method                           NLMINB
##   Number of model parameters                        33
##
##                                                   Used       Total
##   Number of observations                          2571        2576
##   Number of missing patterns                        44
##
## Model Test User Model:
##                                               Standard      Scaled
##   Test Statistic                               243.052     203.874
##   Degrees of freedom                                32          32
##   P-value (Chi-square)                           0.000       0.000
##   Scaling correction factor                                  1.192
##     Yuan-Bentler correction (Mplus variant)
##
## Model Test Baseline Model:
##
##   Test statistic                              1152.043     920.663
##   Degrees of freedom                                45          45
##   P-value                                        0.000       0.000
##   Scaling correction factor                                  1.251
##
## User Model versus Baseline Model:
##
##   Comparative Fit Index (CFI)                    0.809       0.804
##   Tucker-Lewis Index (TLI)                       0.732       0.724
##
##   Robust Comparative Fit Index (CFI)                         1.000
##   Robust Tucker-Lewis Index (TLI)                           -2.086
##
## Loglikelihood and Information Criteria:
##
##   Loglikelihood user model (H0)             -30114.352  -30114.352
##   Scaling correction factor                                  1.411
##       for the MLR correction
##   Loglikelihood unrestricted model (H1)     -29992.826  -29992.826
##   Scaling correction factor                                  1.303
##       for the MLR correction
##
##   Akaike (AIC)                               60294.703   60294.703
##   Bayesian (BIC)                             60487.821   60487.821
##   Sample-size adjusted Bayesian (SABIC)      60382.971   60382.971
##
## Root Mean Square Error of Approximation:
##
##   RMSEA                                          0.051       0.046
##   90 Percent confidence interval - lower         0.045       0.040
##   90 Percent confidence interval - upper         0.057       0.051
##   P-value H_0: RMSEA <= 0.050                    0.416       0.896
##   P-value H_0: RMSEA >= 0.080                    0.000       0.000
```

```
##
##   Robust RMSEA                                              0.000
##   90 Percent confidence interval - lower                    0.000
##   90 Percent confidence interval - upper                    0.000
##   P-value H_0: Robust RMSEA <= 0.050                        1.000
##   P-value H_0: Robust RMSEA >= 0.080                        0.000
##
## Standardized Root Mean Square Residual:
##
##   SRMR                                         0.134       0.134
##
## Parameter Estimates:
##
##   Standard errors                           Sandwich
##   Information bread                         Observed
##   Observed information based on              Hessian
##
## Latent Variables:
##                    Estimate  Std.Err  z-value  P(>|z|)   Std.lv  Std.all
##   int =~
##     is_10             1.000                              11.310    0.505
##     is_11             1.000                              11.310    0.535
##     is_12             1.000                              11.310    0.561
##     is_13             1.000                              11.310    0.511
##     is_14             1.000                              11.310    0.509
##     is_15             1.000                              11.310    0.492
##   quad_slope =~
##     is_10             0.000                                  NA       NA
##     is_11             1.000                                  NA       NA
##     is_12             4.000                                  NA       NA
##     is_13             9.000                                  NA       NA
##     is_14            16.000                                  NA       NA
##     is_15            25.000                                  NA       NA
##   linear_slope =~
##     is_10             0.000                                  NA       NA
##     is_11             1.000                                  NA       NA
##     is_12             2.000                                  NA       NA
##     is_13             3.000                                  NA       NA
##     is_14             4.000                                  NA       NA
##     is_15             5.000                                  NA       NA
##
## Regressions:
##                    Estimate  Std.Err  z-value  P(>|z|)   Std.lv  Std.all
##   is_10 ~
##     blly_10 (d_10)    0.094    0.050    1.876    0.061    0.094    0.061
##   is_12 ~
##     blly_12 (d_12)    0.306    0.034    9.023    0.000    0.306    0.208
##   is_13 ~
##     blly_13 (d_13)    0.305    0.043    7.162    0.000    0.305    0.173
##   is_14 ~
##     blly_14 (d_14)    0.304    0.036    8.387    0.000    0.304    0.169
```

```
##
## Covariances:
##                    Estimate  Std.Err  z-value  P(>|z|)   Std.lv  Std.all
##   int ~~
##     linear_slope     56.561  204.615    0.276    0.782    0.707    0.707
##     quad_slope      -14.900   32.600   -0.457    0.648   -0.901   -0.901
##   bully_10 ~~
##     bully_12         75.805   13.113    5.781    0.000   75.805    0.380
##     bully_13         63.633   52.435    1.214    0.225   63.633    0.349
##     bully_14         62.049   11.569    5.363    0.000   62.049    0.347
##   bully_12 ~~
##     bully_13        113.185   31.272    3.619    0.000  113.185    0.656
##     bully_14         87.542   12.336    7.097    0.000   87.542    0.516
##   bully_13 ~~
##     bully_14         35.659   40.010    0.891    0.373   35.659    0.231
##   quad_slope ~~
##     linear_slope     12.155   28.006    0.434    0.664    1.175    1.175
##
## Intercepts:
##                    Estimate  Std.Err  z-value  P(>|z|)   Std.lv  Std.all
##     bully_10         34.600    0.547   63.253    0.000   34.600    2.383
##     bully_12         33.014    0.482   68.433    0.000   33.014    2.400
##     bully_13         32.237    0.460   70.037    0.000   32.237    2.571
##     bully_14         31.219    0.413   75.519    0.000   31.219    2.532
##     int              28.339    1.748   16.210    0.000    2.506    2.506
##     quad_slope        1.848    0.309    5.990    0.000       NA       NA
##     linear_slope     -8.933    1.831   -4.879    0.000       NA       NA
##
## Variances:
##                    Estimate  Std.Err  z-value  P(>|z|)   Std.lv  Std.all
##    .is_10           372.415  288.022    1.293    0.196  372.415    0.742
##    .is_11           262.872   63.346    4.150    0.000  262.872    0.589
##    .is_12           194.255   27.618    7.034    0.000  194.255    0.477
##    .is_13           243.761   35.982    6.775    0.000  243.761    0.497
##    .is_14           168.535   27.176    6.202    0.000  168.535    0.341
##    .is_15           127.227  125.676    1.012    0.311  127.227    0.241
##     bully_10        210.821   17.528   12.028    0.000  210.821    1.000
##     bully_12        189.162   16.408   11.529    0.000  189.162    1.000
##     bully_13        157.248   14.511   10.837    0.000  157.248    1.000
##     bully_14        152.030   15.543    9.781    0.000  152.030    1.000
##     int             127.906  287.230    0.445    0.656    1.000    1.000
##     quad_slope       -2.136    5.009   -0.426    0.670       NA       NA
##     linear_slope    -50.068  164.559   -0.304    0.761       NA       NA
##
## R-Square:
##                    Estimate
##     is_10             0.258
##     is_11             0.411
##     is_12             0.523
##     is_13             0.503
##     is_14             0.659
```

```
##     is_15            0.759
```

Plot regression coefficients for developmental sensitivity model age 10-15

```
#extract parameters and save
parEst_1015 = parameterEstimates(growth_model_devel1015)

#put into table
table_1 <- parameterEstimates(growth_model_devel1015, standardized = TRUE) %>%
  filter(startsWith(label, c("devsens_"))) %>%
  mutate(Age = str_extract(rhs, "\\d+"),  # extract digits from rhs as Age
         label = "Dev") %>%
  select(Age, label, est, se, std.all, pvalue, ci.lower, ci.upper)

#view table
table_1
```

```
##   Age label   est    se std.all pvalue ci.lower ci.upper
## 1  10   Dev 0.094 0.050   0.061  0.061   -0.004    0.191
## 2  12   Dev 0.306 0.034   0.208  0.000    0.239    0.372
## 3  13   Dev 0.305 0.043   0.173  0.000    0.221    0.388
## 4  14   Dev 0.304 0.036   0.169  0.000    0.233    0.375
```

```
#age as factor for plot
table_1$Age = as.factor(table_1$Age)

#plot regression coefficients
ci_plot_1015 = ggplot(data = table_1, aes(x = Age, y = est, group = 1)) +
  geom_line(aes(group=1), colour = "#c1666b", size = 1) +
  geom_ribbon(aes(ymin = ci.lower, ymax = ci.upper), alpha = 0.2) +
  labs(x = "Age (years)", y = "Regression coefficient and confidence intervals") +
  ggtitle("10-15 years sample") +
  theme_bw(base_size = 11)

#view plot
ci_plot_1015
```

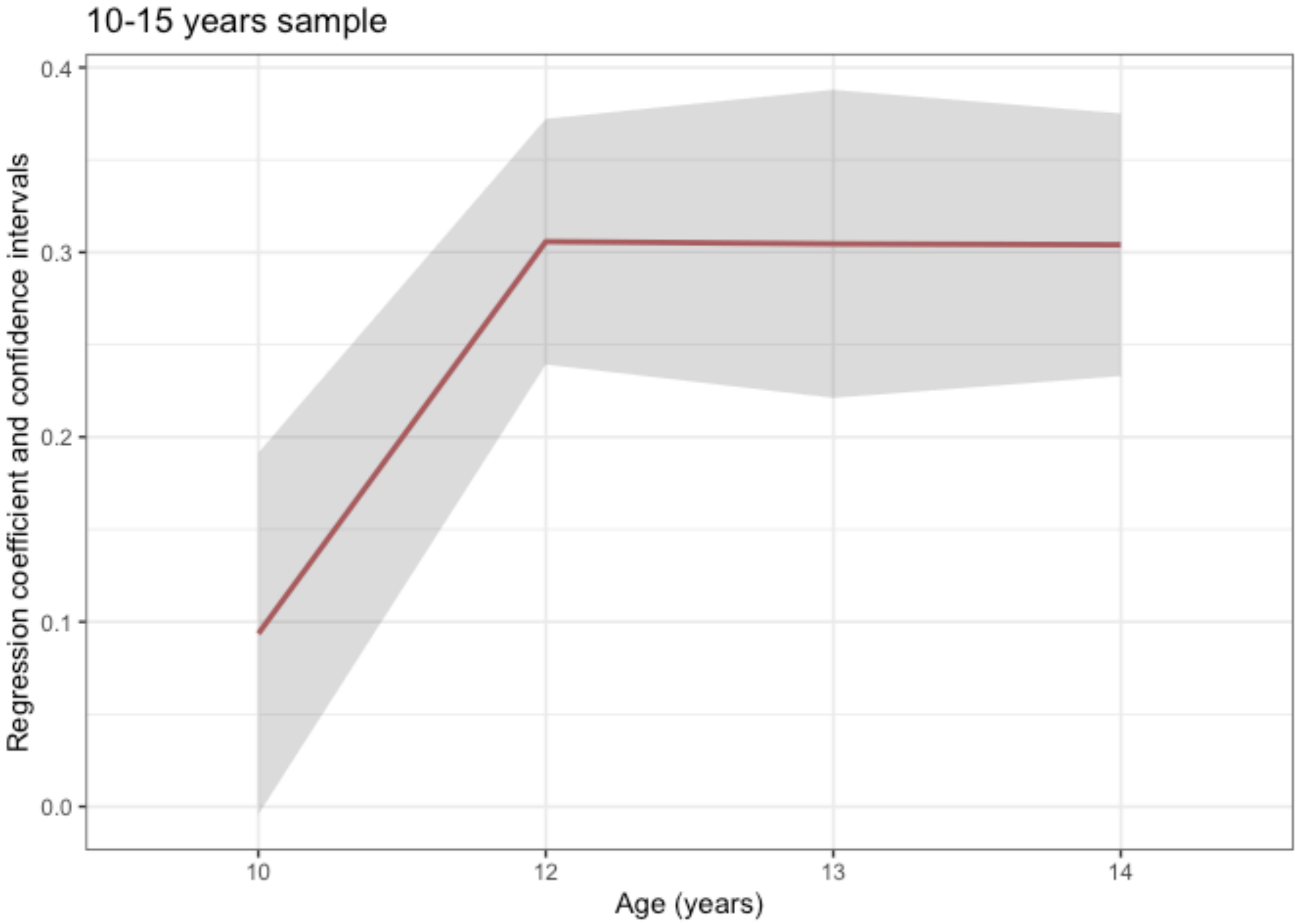

Fit models for age 10-15 years sample (N = 2905)

We followed the same analytic procedure for the 16-21 year sample.

Plot internalising symptoms age 16-21 years

```
#select for plot
older_is = olderyouth_exp %>%
  dplyr::select(pidp, is_16:is_21)

#rename for plot
older_is = older_is %>%
  rename("16" = is_16,
         "17" = is_17,
         "18" = is_18,
         "19" = is_19,
         "20" = is_20,
         "21" = is_21)

#make long
older_is_long = gather(older_is, age, is_score, "16":"21", factor_key = T)
#id column as factor for individual datapoints
older_is_long$pidp = as.factor(older_is_long$pidp)

#plot
older_traj_plot = ggplot(data=older_is_long,aes(x=age, y=is_score)) +
  geom_point(position= "jitter", size=1, alpha=.07, colour = "#4357AD") +
  geom_line(position= "jitter", size=0.7, alpha=.07, aes(group=pidp), colour = "#43
57AD") +
  stat_smooth(aes(group=1), colour="#4357AD", method="loess", se=F, size=2, formula
= 'y~x') +
  theme(legend.position="none", text = element_text(size=9, family='Arial'),
        axis.text.x = element_text(angle=90, vjust=0.5)) +
        labs(x = "Age (years)", y = "Internalising symptoms (scaled from 0-100") +
        theme_bw()

#view plot
older_traj_plot
```

```
## Warning: Removed 9814 rows containing non-finite outside the scale range
## (`stat_smooth()`).
```

```
## Warning: Removed 9814 rows containing missing values or values outside the scale
range
## (`geom_point()`).
```

```
## Warning: Removed 8978 rows containing missing values or values outside the scale
range
## (`geom_line()`).
```

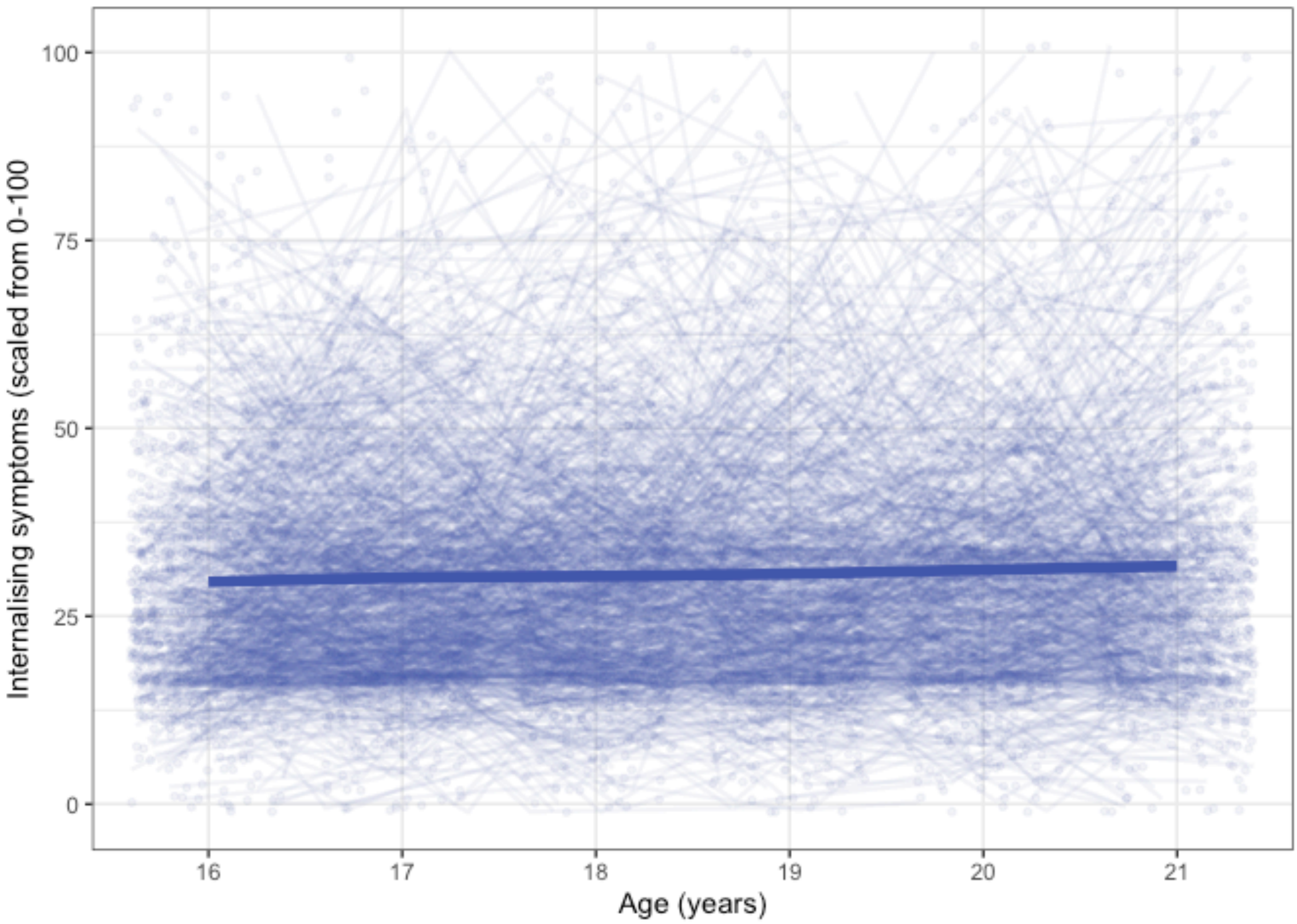

Fit LGM for older youth age 16-21

```
#linear model
lgm_linear_1621<-'

#intercept
int =~ 1*is_16 + 1*is_17 + 1*is_18 + 1*is_19 + 1*is_20 + 1*is_21

#slope
slope =~  0*is_16 + 1*is_17 + 2*is_18 + 3*is_19 + 4*is_20 + 5*is_21

#covariance
int ~~ slope

'

#fit model
linearLGM_older1621 <- growth(lgm_linear_1621,
                              data=olderyouth_exp,
                              estimator='mlr',
                              missing='fiml.x',
                              fixed.x = F)
```

```
## Warning: lavaan->lav_data_full():
##    some cases are empty and will be ignored: 250 908 1785 1923 2250.
```

```
#view fit
fitMeasures(linearLGM_older1621, fit.indices)
```

```
##          chisq             df         pvalue            cfi          rmsea
##         33.445         16.000          0.006          0.988          0.019
## rmsea.ci.lower rmsea.ci.upper           srmr
##          0.010          0.029          0.032
```

```
#view model
summary(linearLGM_older1621,fit.measures=T,standardized=T)
```

```
## lavaan 0.6-20.2283 ended normally after 102 iterations
##
##   Estimator                                         ML
##   Optimization method                           NLMINB
##   Number of model parameters                        11
##
##                                                   Used       Total
##   Number of observations                          2900        2905
##   Number of missing patterns                        63
##
## Model Test User Model:
##                                               Standard      Scaled
##   Test Statistic                                33.445      25.618
##   Degrees of freedom                                16          16
##   P-value (Chi-square)                           0.006       0.060
##   Scaling correction factor                                  1.306
##     Yuan-Bentler correction (Mplus variant)
##
## Model Test Baseline Model:
##
##   Test statistic                              1475.374    1019.581
##   Degrees of freedom                                15          15
##   P-value                                        0.000       0.000
##   Scaling correction factor                                  1.447
##
## User Model versus Baseline Model:
##
##   Comparative Fit Index (CFI)                    0.988       0.990
##   Tucker-Lewis Index (TLI)                       0.989       0.991
##
##   Robust Comparative Fit Index (CFI)                         0.987
##   Robust Tucker-Lewis Index (TLI)                            0.988
##
```

```
## Loglikelihood and Information Criteria:
##
##   Loglikelihood user model (H0)            -31250.127  -31250.127
##   Scaling correction factor                                 1.597
##       for the MLR correction
##   Loglikelihood unrestricted model (H1)    -31233.404  -31233.404
##   Scaling correction factor                                 1.424
##       for the MLR correction
##
##   Akaike (AIC)                              62522.254   62522.254
##   Bayesian (BIC)                            62587.951   62587.951
##   Sample-size adjusted Bayesian (SABIC)     62553.000   62553.000
##
## Root Mean Square Error of Approximation:
##
##   RMSEA                                         0.019       0.014
##   90 Percent confidence interval - lower        0.010       0.003
##   90 Percent confidence interval - upper        0.029       0.023
##   P-value H_0: RMSEA <= 0.050                   1.000       1.000
##   P-value H_0: RMSEA >= 0.080                   0.000       0.000
##
##   Robust RMSEA                                              0.038
##   90 Percent confidence interval - lower                    0.009
##   90 Percent confidence interval - upper                    0.061
##   P-value H_0: Robust RMSEA <= 0.050                        0.780
##   P-value H_0: Robust RMSEA >= 0.080                        0.001
##
## Standardized Root Mean Square Residual:
##
##   SRMR                                          0.032       0.032
##
## Parameter Estimates:
##
##   Standard errors                            Sandwich
##   Information bread                          Observed
##   Observed information based on               Hessian
##
## Latent Variables:
##                    Estimate  Std.Err  z-value  P(>|z|)   Std.lv  Std.all
##   int =~
##     is_16             1.000                               11.208    0.711
##     is_17             1.000                               11.208    0.725
##     is_18             1.000                               11.208    0.716
##     is_19             1.000                               11.208    0.682
##     is_20             1.000                               11.208    0.685
##     is_21             1.000                               11.208    0.648
##   slope =~
##     is_16             0.000                                0.000    0.000
##     is_17             1.000                                2.314    0.150
##     is_18             2.000                                4.627    0.296
##     is_19             3.000                                6.941    0.422
```

```
##     is_20              4.000                                9.254    0.566
##     is_21              5.000                               11.568    0.669
##
## Covariances:
##                    Estimate  Std.Err  z-value  P(>|z|)   Std.lv  Std.all
##   int ~~
##     slope             -8.860    2.597   -3.411    0.001   -0.342   -0.342
##
## Intercepts:
##                    Estimate  Std.Err  z-value  P(>|z|)   Std.lv  Std.all
##     int               28.970    0.339   85.503    0.000    2.585    2.585
##     slope              0.599    0.105    5.712    0.000    0.259    0.259
##
## Variances:
##                    Estimate  Std.Err  z-value  P(>|z|)   Std.lv  Std.all
##    .is_16            122.826   11.397   10.777    0.000  122.826    0.494
##    .is_17            125.718    8.973   14.011    0.000  125.718    0.526
##    .is_18            133.525    8.966   14.893    0.000  133.525    0.545
##    .is_19            149.380   10.693   13.969    0.000  149.380    0.553
##    .is_20            127.419    9.827   12.966    0.000  127.419    0.476
##    .is_21            128.205   12.919    9.924    0.000  128.205    0.429
##     int              125.625    9.773   12.854    0.000    1.000    1.000
##     slope              5.353    0.929    5.765    0.000    1.000    1.000
```

Fit nonlinear models to older youth (basis + quad)

```
#latent basis model
lgm_basis_1621 <-'

#intercept
int =~ 1*is_16 + 1*is_17 + 1*is_18 + 1*is_19 + 1*is_20 + 1*is_21

#slope
basis_slope =~ 0*is_16 + is_17 + is_18 + is_19 + is_20 + 1*is_21

#covariance
int ~~ basis_slope

'

#fit model
basisLGM_older1621 <- growth(lgm_basis_1621,
                             data=olderyouth_exp,
                             estimator='mlr',
                             missing='fiml.x',
                             fixed.x = F)
```

```
## Warning: lavaan->lav_data_full():
##    some cases are empty and will be ignored: 250 908 1785 1923 2250.
```

```
#view fit
fitMeasures(basisLGM_older1621, fit.indices)
```

```
##          chisq             df         pvalue            cfi          rmsea
##         29.257         12.000          0.004          0.988          0.022
## rmsea.ci.lower rmsea.ci.upper           srmr
##          0.012          0.033          0.029
```

```
#view model
summary(basisLGM_older1621,fit.measures=T,standardized=T,rsquare=T)
```

```
## lavaan 0.6-20.2283 ended normally after 169 iterations
##
##   Estimator                                         ML
##   Optimization method                           NLMINB
##   Number of model parameters                        15
##
##                                                   Used       Total
##   Number of observations                          2900        2905
##   Number of missing patterns                        63
##
## Model Test User Model:
##                                               Standard      Scaled
##   Test Statistic                                29.257      24.701
##   Degrees of freedom                                12          12
##   P-value (Chi-square)                           0.004       0.016
##   Scaling correction factor                                  1.184
##     Yuan-Bentler correction (Mplus variant)
##
## Model Test Baseline Model:
##
##   Test statistic                              1475.374    1019.581
##   Degrees of freedom                                15          15
##   P-value                                        0.000       0.000
##   Scaling correction factor                                  1.447
##
## User Model versus Baseline Model:
##
##   Comparative Fit Index (CFI)                    0.988       0.987
##   Tucker-Lewis Index (TLI)                       0.985       0.984
##
##   Robust Comparative Fit Index (CFI)                         0.988
##   Robust Tucker-Lewis Index (TLI)                            0.985
##
```

```
## Loglikelihood and Information Criteria:
##
##   Loglikelihood user model (H0)             -31248.033  -31248.033
##   Scaling correction factor                                  1.616
##       for the MLR correction
##   Loglikelihood unrestricted model (H1)     -31233.404  -31233.404
##   Scaling correction factor                                  1.424
##       for the MLR correction
##
##   Akaike (AIC)                               62526.065   62526.065
##   Bayesian (BIC)                             62615.652   62615.652
##   Sample-size adjusted Bayesian (SABIC)      62567.992   62567.992
##
## Root Mean Square Error of Approximation:
##
##   RMSEA                                          0.022       0.019
##   90 Percent confidence interval - lower         0.012       0.009
##   90 Percent confidence interval - upper         0.033       0.029
##   P-value H_0: RMSEA <= 0.050                    1.000       1.000
##   P-value H_0: RMSEA >= 0.080                    0.000       0.000
##
##   Robust RMSEA                                              0.042
##   90 Percent confidence interval - lower                    0.012
##   90 Percent confidence interval - upper                    0.069
##   P-value H_0: Robust RMSEA <= 0.050                        0.653
##   P-value H_0: Robust RMSEA >= 0.080                        0.007
##
## Standardized Root Mean Square Residual:
##
##   SRMR                                           0.029       0.029
##
## Parameter Estimates:
##
##   Standard errors                             Sandwich
##   Information bread                           Observed
##   Observed information based on                Hessian
##
## Latent Variables:
##                    Estimate  Std.Err  z-value  P(>|z|)   Std.lv  Std.all
##   int =~
##     is_16             1.000                               11.289    0.718
##     is_17             1.000                               11.289    0.731
##     is_18             1.000                               11.289    0.720
##     is_19             1.000                               11.289    0.685
##     is_20             1.000                               11.289    0.689
##     is_21             1.000                               11.289    0.654
##   basis_slope =~
##     is_16             0.000                                0.000    0.000
##     is_17             0.207    0.162    1.280    0.200    2.196    0.142
##     is_18             0.516    0.155    3.327    0.001    5.471    0.349
##     is_19             0.750    0.155    4.842    0.000    7.942    0.482
```

```
##     is_20             0.970    0.148    6.559    0.000   10.276    0.627
##     is_21             1.000                              10.595    0.614
##
## Covariances:
##                    Estimate  Std.Err  z-value  P(>|z|)   Std.lv  Std.all
##   int ~~
##     basis_slope     -43.428   15.009   -2.893    0.004   -0.363   -0.363
##
## Intercepts:
##                    Estimate  Std.Err  z-value  P(>|z|)   Std.lv  Std.all
##     int              28.982    0.444   65.242    0.000    2.567    2.567
##     basis_slope       2.576    0.618    4.167    0.000    0.243    0.243
##
## Variances:
##                    Estimate  Std.Err  z-value  P(>|z|)   Std.lv  Std.all
##    .is_16           119.442   17.648    6.768    0.000  119.442    0.484
##    .is_17           124.282   11.171   11.125    0.000  124.282    0.521
##    .is_18           133.263    8.788   15.164    0.000  133.263    0.542
##    .is_19           146.300   11.112   13.166    0.000  146.300    0.538
##    .is_20           119.994   11.221   10.694    0.000  119.994    0.446
##    .is_21           144.896   15.285    9.480    0.000  144.896    0.487
##     int             127.443   12.033   10.591    0.000    1.000    1.000
##     basis_slope     112.263   25.342    4.430    0.000    1.000    1.000
##
## R-Square:
##                    Estimate
##     is_16             0.516
##     is_17             0.479
##     is_18             0.458
##     is_19             0.462
##     is_20             0.554
##     is_21             0.513
```

```
#fit quadratic
lgm_quad_1621<-'

#intercept
int   =~  1*is_16 + 1*is_17 + 1*is_18 + 1*is_19 + 1*is_20 + 1*is_21

#slopes
linear_slope =~  0*is_16 + 1*is_17 + 2*is_18 + 3*is_19 + 4*is_20 + 5*is_21
quad_slope =~ 0*is_16 + 1*is_17 + 4*is_18 + 9*is_19 + 16*is_20 + 25*is_21

#covariances
int ~~ linear_slope
int ~~ quad_slope

'

#fit model
quadLGM_older1621 <-growth(lgm_quad_1621,
                           data=olderyouth_exp,
                           estimator='mlr',
                           missing='fiml.x',
                           fixed.x = F)
```

```
## Warning: lavaan->lav_data_full():
##    some cases are empty and will be ignored: 250 908 1785 1923 2250.
```

```
#view fit
fitMeasures(quadLGM_older1621, fit.indices)
```

```
##          chisq             df         pvalue            cfi          rmsea
##         11.755         12.000          0.466          1.000          0.000
## rmsea.ci.lower rmsea.ci.upper           srmr
##          0.000          0.019          0.017
```

```
#compare models
lavTestLRT(linearLGM_older1621, basisLGM_older1621)
```

```
##
## Scaled Chi-Squared Difference Test (method = "satorra.bentler.2001")
##
## lavaan->lavTestLRT():
##    lavaan NOTE: The "Chisq" column contains standard test statistics, not the
##    robust test that should be reported per model. A robust difference test is
##    a function of two standard (not robust) statistics.
##                     Df   AIC   BIC  Chisq Chisq diff Df diff Pr(>Chisq)
## basisLGM_older1621  12 62526 62616 29.257
## linearLGM_older1621 16 62522 62588 33.445       2.51       4     0.6428
```

```
lavTestLRT(linearLGM_older1621, quadLGM_older1621) #quad fit better than linear
```

```
##
## Scaled Chi-Squared Difference Test (method = "satorra.bentler.2001")
##
## lavaan->lavTestLRT():
##    lavaan NOTE: The "Chisq" column contains standard test statistics, not the
##    robust test that should be reported per model. A robust difference test is
##    a function of two standard (not robust) statistics.
##                     Df   AIC   BIC  Chisq Chisq diff Df diff Pr(>Chisq)
## quadLGM_older1621   12 62509 62598 11.755
## linearLGM_older1621 16 62522 62588 33.445     16.249       4   0.002702 **
## ---
## Signif. codes:  0 '***' 0.001 '**' 0.01 '*' 0.05 '.' 0.1 ' ' 1
```

Plot predicted linear + quadratic model

```
#save parameters
params1621 <- parameterEstimates(quadLGM_older1621)

# pull out intercept and slope
intercept_mean1621 <- params1621$est[params1621$lhs == "int" & params1621$op == "~
1"]
slope1_mean1621 <- params1621$est[params1621$lhs == "linear_slope" & params$op == "
~1"]  # Linear
slope2_mean1621 <- params1621$est[params1621$lhs == "quad_slope" & params$op == "~
1"]  # Quadratic

# define time points
time <- 0:5

# compute predicted
predicted_values1621 <- intercept_mean1621 + slope1_mean1621 * time + slope2_mean16
21 * time^2

# plot predicted trajectory
plot(time, predicted_values1621, type = "b", pch = 19, col = "#4357AD",
     xlab = "Time", ylab = "Predicted Value",
     main = "Predicted LGM Trajectory (Linear + Quadratic)")
```

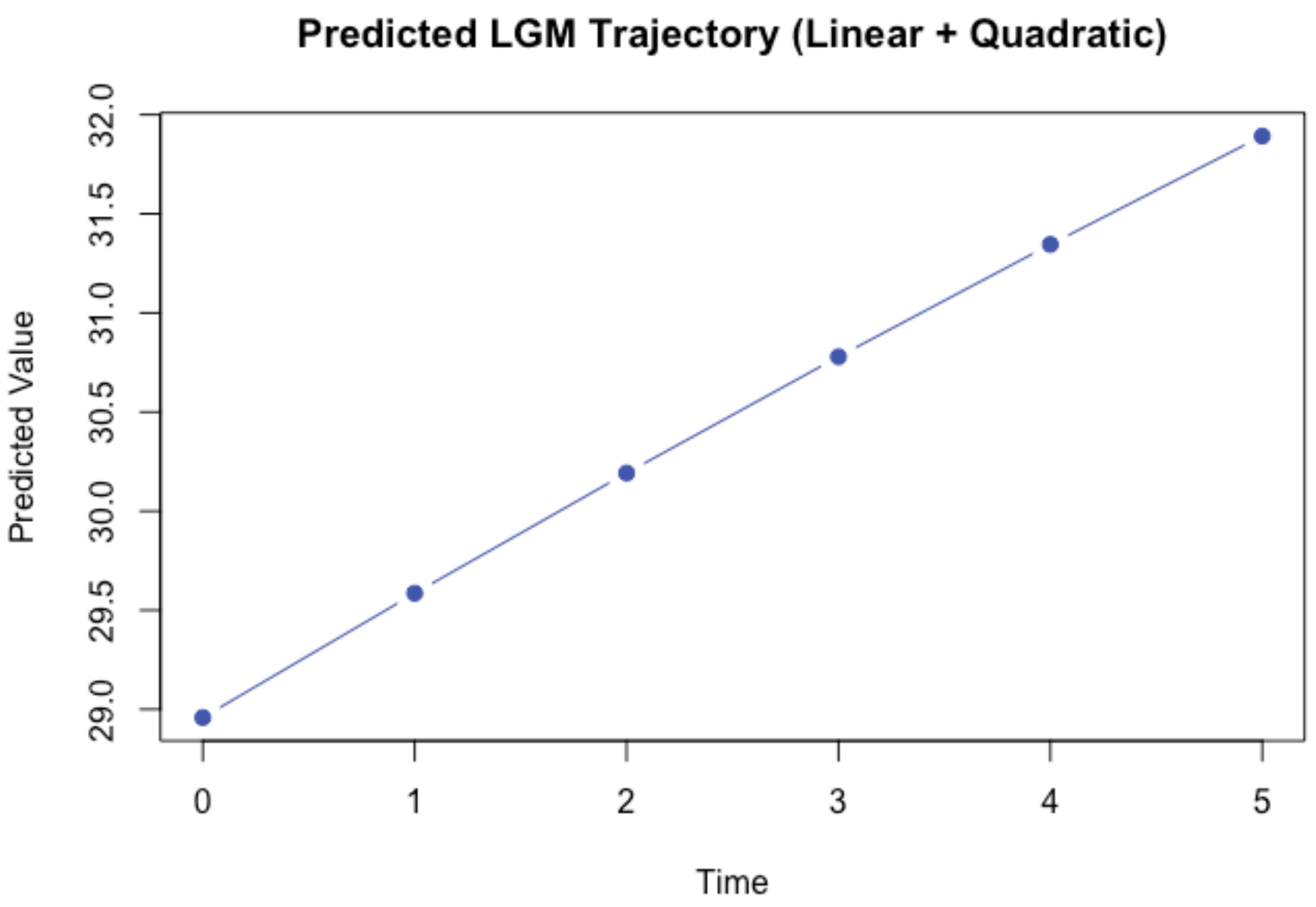

Fit stable sensitivity model to older youth 16-21

Note that due to limited repeated measures social exclusion (se) could only be included for age 18, 19, 20 and 21 years. Internalising symptoms (is) were modeled for age 16-21 years at yearly intervals.

```
#define model
lgm_stable_1621 <-'

int   =~  1*is_16 + 1*is_17 + 1*is_18 + 1*is_19 + 1*is_20 + 1*is_21

#slopes
linear_slope =~  0*is_16 + 1*is_17 + 2*is_18 + 3*is_19 + 4*is_20 + 5*is_21
quad_slope =~ 0*is_16 + 1*is_17 + 4*is_18 + 9*is_19 + 16*is_20 + 25*is_21

#covariance between latent variables
int ~~ linear_slope
int ~~ quad_slope

#regression parameters
is_18 ~ stablesens*se_18
is_19 ~ stablesens*se_19
is_20 ~ stablesens*se_20
is_21 ~ stablesens*se_21

#social exclusion intercepts
se_18 ~ 1
se_19 ~ 1
se_20 ~ 1
se_21 ~ 1

#social exclusion covariances
se_18 ~~ se_19
se_18 ~~ se_20
se_18 ~~ se_21

se_19 ~~ se_20
se_19 ~~ se_21

se_20 ~~ se_21

'

#fit model
growth_model_stable1621 <- growth(lgm_stable_1621,
                                  data=olderyouth_exp,
                                  estimator='mlr',
                                  missing='fiml.x',
                                  fixed.x=FALSE)
```

```
## Warning: lavaan->lav_data_full():
##    some cases are empty and will be ignored: 250 908 1785 1923.
```

```
## Warning: lavaan->lav_data_full():
##    due to missing values, some pairwise combinations have less than 10%
##    coverage; use lavInspect(fit, "coverage") to investigate.
```

```
## Warning: lavaan->lav_mvnorm_missing_h1_estimate_moments():
##    Maximum number of iterations reached when computing the sample moments
##    using EM; use the em.h1.iter.max= argument to increase the number of
##    iterations
```

```
#view fit
fitMeasures(growth_model_stable1621, fit.indices)
```

```
##          chisq             df         pvalue            cfi          rmsea
##        282.742         35.000          0.000          0.870          0.049
## rmsea.ci.lower rmsea.ci.upper           srmr
##          0.044          0.055          0.184
```

Fit developmental sensitivity model for older youth age 16-21 years

Note that due to limited repeated measures social exclusion (se) could only be included for age 18, 19, 20 and 21 years. Internalising symptoms (is) were modeled for age 16-21 years at yearly intervals.

```
#define model
lgm_devel_1621 <-'

int   =~  1*is_16 + 1*is_17 + 1*is_18 + 1*is_19 + 1*is_20 + 1*is_21

#slopes
linear_slope =~  0*is_16 + 1*is_17 + 2*is_18 + 3*is_19 + 4*is_20 + 5*is_21
quad_slope =~ 0*is_16 + 1*is_17 + 4*is_18 + 9*is_19 + 16*is_20 + 25*is_21

#covariance between latent variables
int ~~ linear_slope
int ~~ quad_slope

#regression parameters
is_18 ~ devsens_18*se_18
is_19 ~ devsens_19*se_19
is_20 ~ devsens_20*se_20
is_21 ~ devsens_21*se_21

#social exclusion intercepts
se_18 ~ 1
se_19 ~ 1
se_20 ~ 1
se_21 ~ 1

#social exclusion covariances
se_18 ~~ se_19
se_18 ~~ se_20
se_18 ~~ se_21

se_19 ~~ se_20
se_19 ~~ se_21

se_20 ~~ se_21

'

#fit model
growth_model_devel1621 <- growth(lgm_devel_1621,
                                 data=olderyouth_exp,
                                 estimator='mlr',
                                 missing='fiml.x',
                                 fixed.x=FALSE)
```

```
## Warning: lavaan->lav_data_full():
##    some cases are empty and will be ignored: 250 908 1785 1923.
```

```
## Warning: lavaan->lav_data_full():
##    due to missing values, some pairwise combinations have less than 10%
##    coverage; use lavInspect(fit, "coverage") to investigate.
```

```
## Warning: lavaan->lav_mvnorm_missing_h1_estimate_moments():
##    Maximum number of iterations reached when computing the sample moments
##    using EM; use the em.h1.iter.max= argument to increase the number of
##    iterations
```

```
#view fit
fitMeasures(growth_model_devel1621, fit.indices)
```

```
##          chisq             df         pvalue            cfi          rmsea
##        199.517         32.000          0.000          0.912          0.042
## rmsea.ci.lower rmsea.ci.upper           srmr
##          0.037          0.048          0.153
```

Compare stable and developmental sensitivity models older youth (16-21 years)

```
#compare models
lavTestLRT(growth_model_stable1621, growth_model_devel1621) # devel model fits bett
er
```

```
##
## Scaled Chi-Squared Difference Test (method = "satorra.bentler.2001")
##
## lavaan->lavTestLRT():
##    lavaan NOTE: The "Chisq" column contains standard test statistics, not the
##    robust test that should be reported per model. A robust difference test is
##    a function of two standard (not robust) statistics.
##                         Df   AIC   BIC  Chisq Chisq diff Df diff Pr(>Chisq)
## growth_model_devel1621  32 73172 73369 199.52
## growth_model_stable1621 35 73249 73429 282.74     63.668       3  9.668e-14 ***
## ---
## Signif. codes:  0 '***' 0.001 '**' 0.01 '*' 0.05 '.' 0.1 ' ' 1
```

```
#view devel model
summary(growth_model_devel1621,fit.measures=T,standardized=T,rsquare=T)
```

```
## lavaan 0.6-20.2283 ended normally after 266 iterations
##
##   Estimator                                         ML
##   Optimization method                           NLMINB
```

```
##   Number of model parameters                        33
##
##                                                   Used       Total
##   Number of observations                          2901        2905
##   Number of missing patterns                       206
##
## Model Test User Model:
##                                               Standard      Scaled
##   Test Statistic                               199.517     172.015
##   Degrees of freedom                                32          32
##   P-value (Chi-square)                           0.000       0.000
##   Scaling correction factor                                  1.160
##     Yuan-Bentler correction (Mplus variant)
##
## Model Test Baseline Model:
##
##   Test statistic                              1947.034    1547.521
##   Degrees of freedom                                45          45
##   P-value                                        0.000       0.000
##   Scaling correction factor                                  1.258
##
## User Model versus Baseline Model:
##
##   Comparative Fit Index (CFI)                    0.912       0.907
##   Tucker-Lewis Index (TLI)                       0.876       0.869
##
##   Robust Comparative Fit Index (CFI)                         0.882
##   Robust Tucker-Lewis Index (TLI)                            0.834
##
## Loglikelihood and Information Criteria:
##
##   Loglikelihood user model (H0)             -36553.109  -36553.109
##   Scaling correction factor                                  1.303
##       for the MLR correction
##   Loglikelihood unrestricted model (H1)     -36453.351  -36453.351
##   Scaling correction factor                                  1.232
##       for the MLR correction
##
##   Akaike (AIC)                               73172.218   73172.218
##   Bayesian (BIC)                             73369.321   73369.321
##   Sample-size adjusted Bayesian (SABIC)      73264.468   73264.468
##
## Root Mean Square Error of Approximation:
##
##   RMSEA                                          0.042       0.039
##   90 Percent confidence interval - lower         0.037       0.034
##   90 Percent confidence interval - upper         0.048       0.044
##   P-value H_0: RMSEA <= 0.050                    0.985       1.000
##   P-value H_0: RMSEA >= 0.080                    0.000       0.000
##
##   Robust RMSEA                                               0.110
```

```
##   90 Percent confidence interval - lower                        0.087
##   90 Percent confidence interval - upper                        0.133
##   P-value H_0: Robust RMSEA <= 0.050                            0.000
##   P-value H_0: Robust RMSEA >= 0.080                            0.985
##
## Standardized Root Mean Square Residual:
##
##   SRMR                                           0.153       0.153
##
## Parameter Estimates:
##
##   Standard errors                             Sandwich
##   Information bread                           Observed
##   Observed information based on                Hessian
##
## Latent Variables:
##                    Estimate  Std.Err  z-value  P(>|z|)   Std.lv  Std.all
##   int =~
##     is_16             1.000                               11.967    0.772
##     is_17             1.000                               11.967    0.764
##     is_18             1.000                               11.967    0.761
##     is_19             1.000                               11.967    0.734
##     is_20             1.000                               11.967    0.743
##     is_21             1.000                               11.967    0.716
##   linear_slope =~
##     is_16             0.000                                0.000    0.000
##     is_17             1.000                                6.273    0.401
##     is_18             2.000                               12.547    0.798
##     is_19             3.000                               18.820    1.154
##     is_20             4.000                               25.093    1.557
##     is_21             5.000                               31.367    1.877
##   quad_slope =~
##     is_16             0.000                                0.000    0.000
##     is_17             1.000                                1.125    0.072
##     is_18             4.000                                4.498    0.286
##     is_19             9.000                               10.121    0.620
##     is_20            16.000                               17.992    1.117
##     is_21            25.000                               28.113    1.682
##
## Regressions:
##                    Estimate  Std.Err  z-value  P(>|z|)   Std.lv  Std.all
##   is_18 ~
##     se_18   (d_18)    0.054    0.012    4.669    0.000    0.054    0.077
##   is_19 ~
##     se_19   (d_19)    0.117    0.016    7.287    0.000    0.117    0.159
##   is_20 ~
##     se_20   (d_20)    0.212    0.024    8.947    0.000    0.212    0.301
##   is_21 ~
##     se_21   (d_21)    0.330    0.036    9.254    0.000    0.330    0.443
##
## Covariances:
```

```
##                    Estimate  Std.Err  z-value  P(>|z|)   Std.lv  Std.all
##   int ~~
##     linear_slope    -28.081   12.117   -2.317    0.020   -0.374   -0.374
##     quad_slope        2.754    2.128    1.294    0.196    0.205    0.205
##   se_18 ~~
##     se_19           227.752   34.595    6.583    0.000  227.752    0.459
##     se_20           221.058   45.990    4.807    0.000  221.058    0.433
##     se_21           240.906   56.156    4.290    0.000  240.906    0.482
##   se_19 ~~
##     se_20           209.790   41.884    5.009    0.000  209.790    0.410
##     se_21           231.201   42.544    5.434    0.000  231.201    0.462
##   se_20 ~~
##     se_21           292.527   35.139    8.325    0.000  292.527    0.569
##   linear_slope ~~
##     quad_slope       -6.587    2.047   -3.218    0.001   -0.934   -0.934
##
## Intercepts:
##                    Estimate  Std.Err  z-value  P(>|z|)   Std.lv  Std.all
##     se_18            52.116    1.128   46.191    0.000   52.116    2.342
##     se_19            52.941    1.166   45.409    0.000   52.941    2.373
##     se_20            51.603    1.188   43.424    0.000   51.603    2.251
##     se_21            51.636    1.142   45.216    0.000   51.636    2.301
##     int              29.149    0.408   71.458    0.000    2.436    2.436
##     linear_slope      0.633    0.552    1.148    0.251    0.101    0.101
##     quad_slope       -0.702    0.135   -5.187    0.000   -0.624   -0.624
##
## Variances:
##                    Estimate  Std.Err  z-value  P(>|z|)   Std.lv  Std.all
##    .is_16            97.318   16.412    5.929    0.000   97.318    0.405
##    .is_17           125.334    9.077   13.808    0.000  125.334    0.511
##    .is_18           120.728    9.171   13.164    0.000  120.728    0.488
##    .is_19           134.202   10.239   13.107    0.000  134.202    0.504
##    .is_20           119.220    9.335   12.772    0.000  119.220    0.459
##    .is_21            96.918   18.836    5.145    0.000   96.918    0.347
##     se_18           495.296   31.099   15.926    0.000  495.296    1.000
##     se_19           497.559   32.151   15.476    0.000  497.559    1.000
##     se_20           525.603   34.065   15.429    0.000  525.603    1.000
##     se_21           503.570   32.351   15.566    0.000  503.570    1.000
##     int             143.197   15.809    9.058    0.000    1.000    1.000
##     linear_slope     39.355   11.093    3.548    0.000    1.000    1.000
##     quad_slope        1.265    0.404    3.127    0.002    1.000    1.000
##
## R-Square:
##                    Estimate
##     is_16             0.595
##     is_17             0.489
##     is_18             0.512
##     is_19             0.496
##     is_20             0.541
##     is_21             0.653
```

Plot regression coefficients for developmental sensitivity model age 16-21

```
#save parameter estimates
parEst_1621 = parameterEstimates(growth_model_devel1621)

#create table
table_2 <- parameterEstimates(growth_model_devel1621, standardized = TRUE) %>%
  filter(startsWith(label, c("devsens_"))) %>%
  mutate(Age = str_extract(rhs, "\\d+"),  # extract digits from rhs as Age
         label = "Dev") %>%
  select(Age, label, est, se, std.all, pvalue, ci.lower, ci.upper)

#view table
table_2
```

```
##   Age label   est    se std.all pvalue ci.lower ci.upper
## 1  18   Dev 0.054 0.012   0.077      0    0.031    0.077
## 2  19   Dev 0.117 0.016   0.159      0    0.085    0.148
## 3  20   Dev 0.212 0.024   0.301      0    0.165    0.258
## 4  21   Dev 0.330 0.036   0.443      0    0.260    0.400
```

```
#age as factor for plot
table_2$Age = as.factor(table_2$Age)
#plot
ci_plot_1621 = ggplot(data = table_2, aes(x = Age, y = est, group = 1)) +
  geom_line(aes(group=1), colour = "#4357AD", size = 1) +
  geom_ribbon(aes(ymin = ci.lower, ymax = ci.upper), alpha = 0.2) +
  labs(x = "Age (years)", y = "Regression coefficient and confidence intervals") +
  ggtitle("16-21 years sample") +
  theme_bw(base_size = 11)
#view plot
ci_plot_1621
```

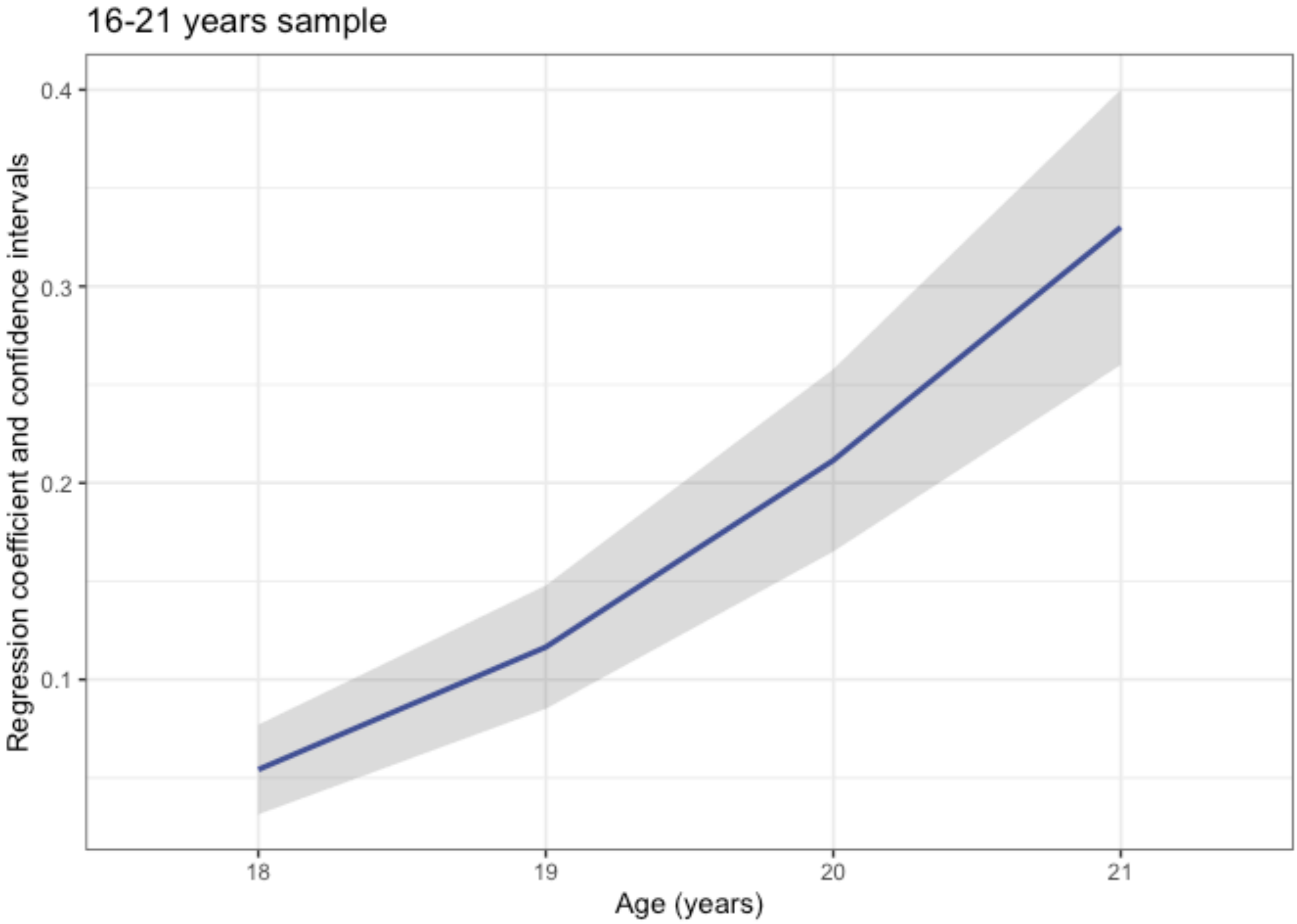
16-21 years sample
Regression coefficient and confidence intervals
0.4
0.3
0.2
0.1
18
19
20
21
Age (years)
